# Asymmetric Mannich reaction of aromatic imines with malonates in the presence of multifunctional catalysts

**DOI:** 10.3762/bjoc.22.8

**Published:** 2026-01-16

**Authors:** Kadri Kriis, Harry Martõnov, Annette Miller, Mia Peterson, Ivar Järving, Tõnis Kanger

**Affiliations:** 1 Department of Chemistry and Biotechnology, Tallinn University of Technology, Akadeemia tee 15, 12618 Tallinn, Estonia

**Keywords:** aromatic imine, asymmetric catalysis, Mannich reaction, noncovalent interactions, organocatalysis

## Abstract

Various multifunctional enantiomerically pure organocatalysts were synthesized and screened in asymmetric Mannich reaction. The reaction of aromatic imines with malonates in the presence of amino acid-derived catalysts gave Mannich adducts in very high enantiomeric purities (up to 98% ee). It is proposed that a network of hydrogen and halogen bonds with Lewis bases, together with the steric effect of the *tert*-butyl group of the catalyst, is responsible for the high stereoselectivity of the reaction.

## Introduction

The Mannich reaction, i.e., the addition of an enolized carbonyl compound to an imine derived from an aldehyde or ketone and an amine, has been known for more than hundred years [1] and it has become an important method for creating C–C bonds [2–4]. The obtained Mannich bases exhibit a broad spectrum of biological activities [5–6] and have also been used in the synthesis of numerous pharmaceuticals and natural products [7]. The application of asymmetric synthesis enables access to enantiomerically pure targets. Earlier, metal catalysis was used for Mannich reactions [8–9], but in recent years, methods of asymmetric organocatalysis have been widely used to achieve these valuable compounds [10–12]. Enamine activation was one of the first organocatalytic methods applied in Mannich reactions [13–14]. Concurrently, hydrogen-bond catalysis has emerged as a significant strategy for promoting asymmetric Mannich reactions [15–16]. This has led to the use of multifunctional catalysts of “homocooperativity,” where multiple units of the same catalyst perform different but complementary roles [17]. These catalysts employing noncovalent interactions via hydrogen bonds and also possess Lewis basic and π–π-interaction sites have been highly efficient in Mannich reactions, as demonstrated by our studies [18] and those of others [19–21]. Halogen bonding has also been introduced to further broaden the field of noncovalent interactions. Despite the limited number of asymmetric transformations driven solely by halogen-bond activation [22], halogen bonding is more frequently employed as a component of multifunctional catalytic systems [23–25]. Multifunctionality of the catalyst is essential to intensify weak noncovalent interactions.

## Results and Discussion

**Scheme 1:**
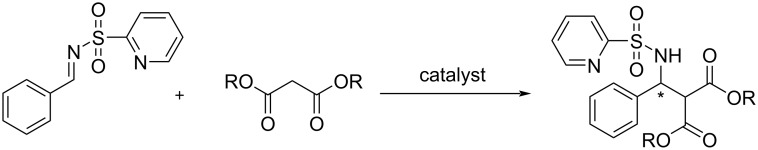
The catalytic Mannich reaction under study.

As a continuation of our previous studies [18,24], we now report an asymmetric Mannich reaction between a 2-sulfonylpyridine protected imine and a malonate (Scheme 1). The pyridine-containing protecting group was chosen considering our catalyst design. We envisioned that halogen bond (XB) will complement hydrogen bonds to activate an imine towards the nucleophilic attack of the malonate. It is known that N-atoms are the strongest XB acceptors in electroneutral compounds [26]. It has also been shown that imines protected with heteroarylsulfonyl groups provide higher enantioselectivity than those protected with the more commonly used phenylsulfonyl or tosyl groups in addition reactions to imines [27].

**Figure 1:**
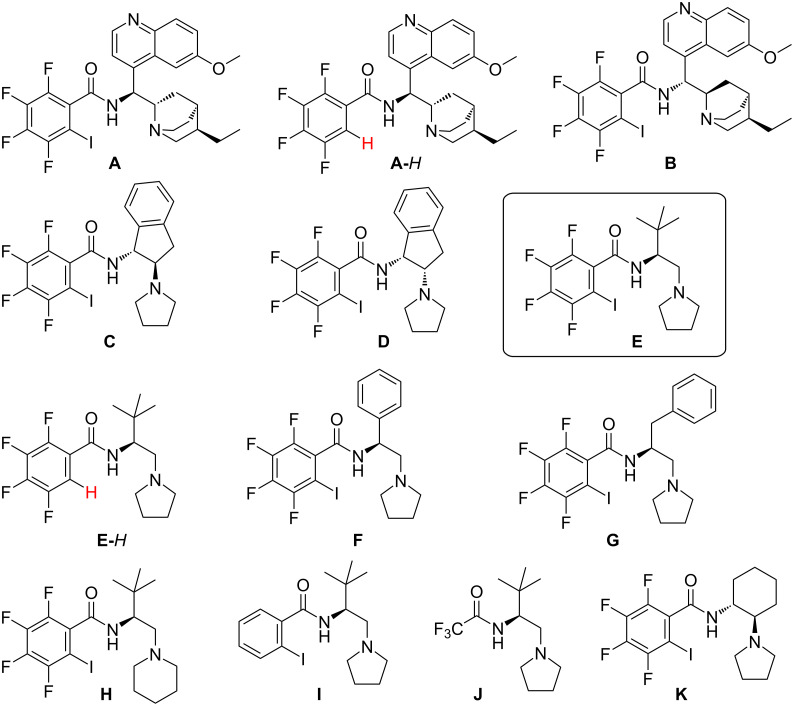
Screened catalysts.

The model reaction was performed in the presence of various types of catalysts (Figure 1).

The syntheses of the catalysts are described in Supporting Information File 1. All catalysts used are amides containing tertiary amino groups, which are needed for the activation of the malonate. We have previously shown the importance of an acidic amide proton in asymmetric Mannich reactions [18]. The majority of the catalysts are also potential halogen-bond donors containing tetrafluoroiodophenyl or iodophenyl moieties, which enable the formation of additional noncovalent interactions between the catalyst and the reagents. The chirality of the catalysts is derived from either amino acid or amino alcohols (including cinchona alkaloid derivatives). There are three exceptional structures: catalysts **A**-*H* and **E**-*H*, which are the hydrogen analogues of the corresponding iodine-containing compounds, and catalyst **J** as a trifluoroacetylated chiral tertiary amine, which lacks iodine.

The reaction of imine **1** with dimethyl malonate was selected for the investigation as a model reaction. Based on our previous experience the catalyst screening was carried out in toluene in the presence of 10 mol % of the catalyst and it was started with Arai-type catalyst **A** [19]. The features of the catalyst are the quinine moiety with reduced double bond and enhanced halogen-bonding ability due to the highly electronegative substituents on the phenyl ring. The reaction was relatively fast, achieving full conversion of the starting imine within 3 hours at rt and in 5 hours at −20 °C. Decrease of the temperature increases the enantiomeric purity of the product (from 27% ee up to 52% ee) (Table 1, entries 1–3). The corresponding *H*-analogue of catalyst **A** was less selective (18% ee) (Table 1, entries 4 and 5). This suggests that possibly the halogen bond plays a role in the emergence of the stereoselectivity of the reaction. The reaction with the similar quinidine derivative with reduced double bond (catalyst **B**) was slower and less selective affording the opposite enantiomer in low enantiomeric purity (14% ee, Table 1, entry 6).

**Table 1:** Catalyst screening.

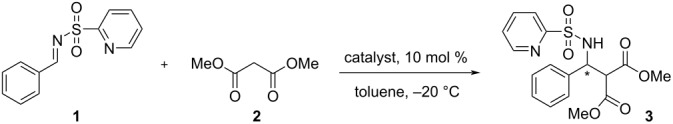			

Entry	Catalyst	NMR conv % (time, h)	ee (%)

1	**A**	100 (3)	27^a^
2	**A**	100 (3)	38^b^
3	**A**	100 (5)	52
4	**A-** *H*	99 (3)	18^b^
5	**A-** *H*	93 (5)	18
6	**B**	85 (5)	−14^c^
7	**C**	24 (48)	ND
8	**D**	10 (48)	ND
9	**E**	89 (5)	83
10	**E**-*H*	93 (5)	71
11	**F**	93 (48)	28
12	**G**	99 (48)	38
13	**H**	98 (22)	78
14	**I**	100 (5)	78
15	**J**	100 (24)	74
16 17^d^	**K** **E**	97 (5) 96 (5)	62 44

^a^Reaction at rt; ^b^reaction at 0 °C; ^c^an opposite enantiomer was obtained; ^d^*para*-toluenesulfonyl protected imine was used instead of imine **1**.

Aminoindane-based catalysts **C** and **D** were inefficient and stereomeric purity of the products were not determined (Table 1, entries 7 and 8). The next group of catalysts consists of amino acid derivatives. The most selective was *tert*-leucine-based catalyst **E** affording the Mannich adduct in 83% ee (Table 1, entry 9). Replacing the five-membered pyrrolidine ring in the catalyst structure with the more flexible six-membered piperidine ring (catalyst **H**, Table 1, entry 13) reduced its selectivity slightly (78% ee). However, the catalyst derived from phenylglycine (catalyst **F**) or phenylalanine (catalyst **G**) were much less selective (Table 1, entries 11 and 12, respectively). To evaluate the role of potential halogen bonds, the corresponding hydrogen analogue of catalyst **E** was synthesized and used in the model reaction (Table 1, entry 10). The catalyst was still efficient but afforded the product in a slightly less enantioselective way (71% ee). When comparing the halogen bond donor properties of tetrafluoroiodophenyl and iodophenyl moieties, the latter is weaker as it is less electron-withdrawing. However, there is a very small difference between reactivity and selectivity for catalysts **E** and **I**. Catalyst **J** lacking the iodophenyl core was much less reactive and 24 h were needed to reach full conversion of the starting imine. However, the stereoselectivity remains high and is comparable to that in the previous examples. Our results demonstrate that with *tert-*leucine-derived catalyst **E** the halogen bond is not essential for the stereoselectivity of the reaction, but the halogen atom has a beneficial effect on both reactivity and stereoselectivity. A comparative experiment using an imine with a *para*-toluenesulfonyl protecting group afforded the product in lower selectivity (Table 1, entry 17), further highlighting the importance of the pyridine ring containing protecting group. The interaction between catalyst **E** and the imine was further investigated by ^19^F NMR studies (see Supporting Information File 1).

To further elucidate the role of noncovalent interactions, we conducted additional experiments using catalyst **K** and imine **1f** (Table 2). Catalyst **K** derived from 1,2-diaminocyclohexane contains similarly to the others a tetrafluoroiodophenyl moiety and gave product **3f** with moderate selectivity (**3f**, 63% ee) (Table 2, entry 1). Its hydrogen derivative **K**-*H* was slightly less selective (Table 2, entry 2). Substituting a sterically demanding iodine atom in the phenyl ring with the methyl group (catalyst **K**-*Me*) increased the stereoselectivity (Table 2, entry 3, 56% ee). However, the *N*-methylated catalyst led to a diminished reaction rate and exhibited complete loss of selectivity, resulting in the formation of a racemic product (Table 2, entry 4).

**Table 2:** Reaction in the presence of catalyst **K** and its derivatives.

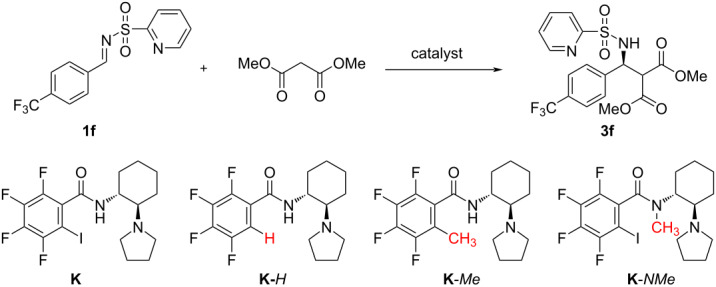			

Entry	Catalyst	NMR conv % (time, h)	ee (%)

1.	**K**	97 (5)	63
2.	**K**-*H*	95 (5)	49
3.	**K**-*Me*	98 (5)	56
4.	**K**-*NMe*	41 (24)	0

Similar trends with catalyst **E** were observed: the amidic proton is essential to achieve high reactivity and selectivity and changing the halogen atom with other groups slightly decreases the stereoselectivity of the reaction. Steric effects of the iodine atom cannot be excluded in the emergence of the stereoselectivity.

To determine the absolute configuration of product **3** the nitrogen atom was additionally protected with Boc_2_O followed by the removal of the pyridinesulfonyl group with Mg in MeOH (see Supporting Information File 1) [28]. Although basic conditions caused partial racemization during the protection or deprotection step, the Boc-protected amine was obtained in low enantiomeric purity. Chiral HPLC analysis and comparison with an authentic sample revealed the *S*-configuration of the Mannich adduct [29]. Absolute configurations of other products were assigned by analogy.

**Figure 2:**
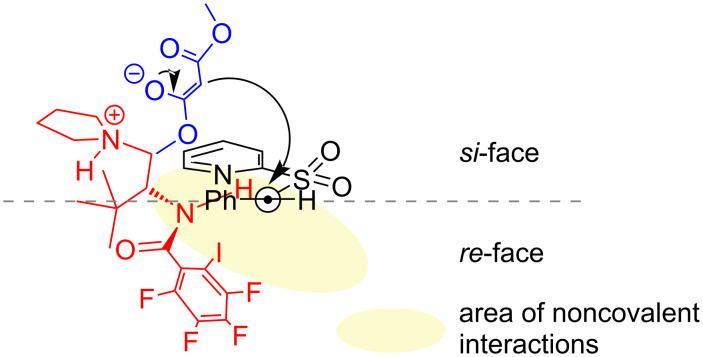
Model for the interaction of the catalyst with the imine.

Based on the results obtained in catalyst screening, it is assumed that the catalyst and the imine are connected through a network of hydrogen or halogen bonds. Iodine may form halogen bonds with either of the nitrogen atoms of the imine. Halogen bonds can be strengthened via hydrogen-bond-assisted halogen bonding [26,30]. Similarly, hydrogen bonds with the amidic proton are also possible. The direct effect of a specific interaction could not be determined based on the obtained data. The network of noncovalent interactions formed stabilizes the complex between the catalyst and the imine and malonate attacks from *si*-face affording the product in *S*-configuration (Figure 2).

**Figure 3:**
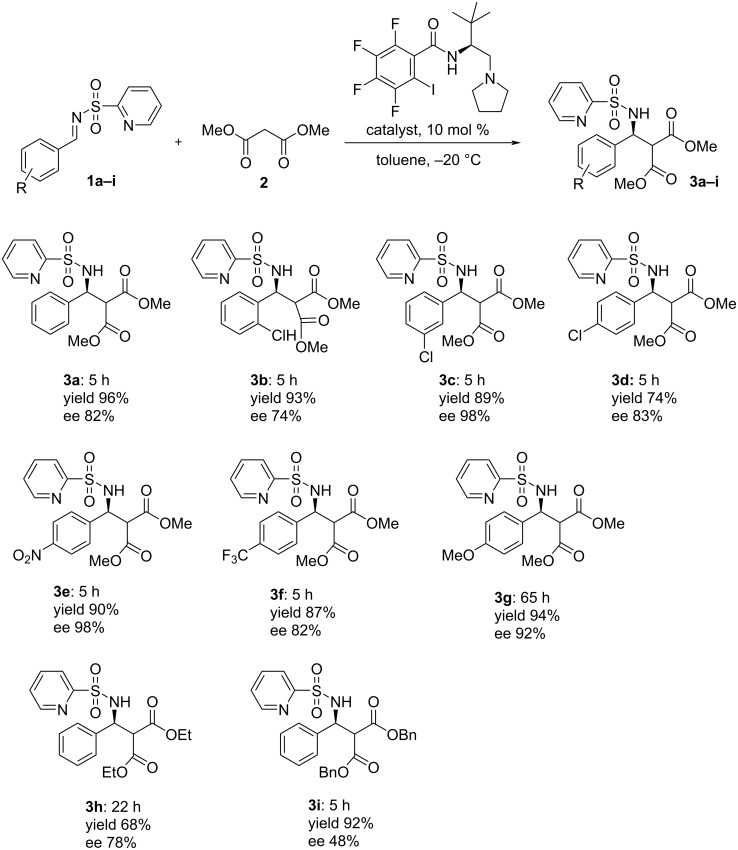
Substrate scope of the asymmetric Mannich reaction.

To demonstrate the utility of the elaborated catalytic system, the Mannich reaction between aromatic imines, both with electron-withdrawing and electron-donating substituents in the phenyl ring, and malonate were carried out in the presence of 10 mol % of catalyst **E** in toluene at −20 °C (Figure 3). The experimental procedure was simple, enantiomerically enriched products **3a**–**i** were isolated by direct precipitation from the crude reaction mixture in good to high yields by adding a mixture of petroleum ether/Et_2_O (see the Experimental section).

Generally, the reactions are fast and afford products in high to excellent enantiomeric purities. The substitution pattern in the phenyl ring has some influence on the results (compare compounds **3a**–**d**). The *ortho*-Cl substituent in the aromatic ring of **1b** slightly decreases while the *meta*-position of Cl atom in **1c** increases the enantioselectivity of reaction (compare **3b** and **3c**). An electron-donating substituent decreases the rate substantially but the stereoselectivity of the reaction remains still high (compound **3g**). Diethyl malonate proved to be less reactive under standard conditions than dimethyl malonate and dibenzyl malonate gave the product with high yield, but the selectivity dropped significantly (compound **3i**).

## Conclusion

In conclusion, we have designed a new catalyst enabling highly enantioselective Mannich reactions of aromatic imines. Although the activation mechanism was not proved unambiguously, it is assumed that steric effects of the chiral fragment together with a network of noncovalent interactions, including halogen and hydrogen bonds, are responsible for the high enantioselectivity of the reaction. Further developments of the method and applications of obtained products are under study.

## Experimental

### General procedure for the catalytic asymmetric Mannich reaction

Catalyst **E** (2.7 mg, 0.0057 mmol, 0.01 equiv) was weighed into a reaction vessel, imine (0.057 mmol, 1.0 equiv) and toluene (285 µL) were added. The mixture was stirred at room temperature until a suspension was formed (ca 5 min). After that, the reaction mixture was cooled to −20 °C. The malonic ester (0.171 mmol, 3.0 equiv) was added to the reaction vessel via syringe. The reaction was stirred at −20 °C for an appropriate time. The progress of the reaction was monitored by ^1^H NMR analysis. After completion of the reaction, the product was isolated by direct precipitation from the crude reaction mixture by adding a mixture of petroleum ether/Et_2_O (4:1; 2 mL). The product was collected by filtration and washed with a mixture of petroleum ether/Et_2_O (4:1; 4 × 2 mL).

## Supporting Information

File 1 Experimental procedures, synthetic details, NMR spectra, chiral HPLC chromatograms.

## Data Availability

All data that supports the findings of this study is available in the published article and/or the supporting information of this article.
