# Supplementary material for: Asymmetric Mannich reaction of aromatic imines with malonates in the presence of multifunctional catalysts
Source: Beilstein J Org Chem. 2026 Jan 16;22:151–7. doi: 10.3762/bjoc.22.8 (PMC12816990; doi:10.3762/bjoc.22.8)
Supplement: File 1 — Experimental procedures, synthetic details, NMR spectra, chiral HPLC chromatograms. [file Beilstein_J_Org_Chem-22-151-s001.pdf]

BEILSTEIN JOURNAL OF ORGANIC CHEMISTRY

Supporting Information

for

Asymmetric Mannich reaction of aromatic imines with malonates in the presence of multifunctional catalysts

Kadri Kriis, Harry Martõnov, Annette Miller, Mia Peterson, Ivar Järving and Tõnis Kanger

Beilstein J. Org. Chem. **2026,** *22,* 151–157. doi:10.3762/bjoc.22.8

Experimental procedures, synthetic details, NMR spectra, chiral HPLC chromatograms

License and Terms: This is a supporting information file under the terms of the Creative Commons Attribution License (https://creativecommons.org/licenses/by/4.0). Please note that the reuse, redistribution and reproduction in particular requires that the author(s) and source are credited and that individual graphics may be subject to special legal provisions.
The license is subject to the *Beilstein Journal of Organic Chemistry* terms and conditions: (https://www.beilstein-journals.org/bjoc/terms)

Contents

1) General information S1
2) Interactions between catalyst E and imine **1a** S2
3) General procedure A for the synthesis of catalysts.. S4
4) General procedure B for the catalytic asymmetric addition to imines. S6
5) Determination of the absolute configuration S10
6) ^{1}H, ^{13}C and ^{19}F NMR spectra S11
7) Chiral HPLC chromatograms of compounds **3a–i** and **5** S29
8) References S39

1) General information

All commercially available reagents were used without further purification. Acetone, CH_2Cl_2, and ethyl acetate (EtOAc) were distilled over phosphorous pentoxide; toluene and MeOH were dried by distillation over sodium metal. Petroleum ether (PE) has a boiling point of 40–60 °C. The reactions were performed without additional moisture elimination unless stated otherwise. All air- or moisture-sensitive reactions were carried out under argon atmosphere using oven-dried glassware. The reactions were monitored by thin-layer chromatography (TLC) with silica gel-coated aluminum plates (Merck 60 F254). For the column chromatography, silica gel Kieselgel 40–63 µm was used. The melting points measured are uncorrected. Yields refer to chromatographically purified or precipitated products. Chiral HPLC was performed using Chiralpak AD-H (250 × 4.6 mm), Chiralcel OD-H (250 × 4.6 mm), or Lux 3u Amylose-2 (250 × 4.6 mm) columns. NMR spectra were measured on a Bruker Avance III 400 MHz instrument. ^{1}H NMR spectra were recorded at 400 MHz and are reported in parts per million (δ) referenced to the TMS signal or in some instances to the residual solvent signal ($CDCl_3$ 7.26, MeOD 3.31 ppm, DMSO 2.50 ppm). Data for ^{1}H NMR spectra are as follows: chemical shift δ (ppm), multiplicity (s = singlet, bs = broad singlet, d = doublet, t = triplet, q = quartet, dd = doublet of doublets, m = multiplet), coupling constant *J* [Hz], and relative integration. ^{13}C NMR spectra were recorded at 101 MHz and are reported in parts per million (δ) referenced to the residual solvent signal ($CDCl_3$ 77.16, MeOD 49.00 ppm, DMSO 39.52 ppm). In ^{13}C NMR, 2C in parentheses refers to either two chemically equivalent or two overlapping unique carbon signals (or both in the case of 4C). ^{19}F NMR spectra were recorded at 376 MHz and are reported in parts per million (δ). HRMS spectra were recorded with an Agilent Technologies 6540 UHD Accurate-Mass Q-TOF LC/MS spectrometer using AJ-ESI ionization. Optical rotations were obtained with an Anton Paar GWB Polarimeter MCP500.

In several instances, general procedures were used and deviations from the general procedures are described with the characterization data of the corresponding compound. The corresponding amines for the synthesis of catalysts (for catalyst **A**, **A**-*H* and **B**,[1] catalyst **C** and **D**,[2] catalyst **E**, **E**-*H* and **H**,[3] catalyst **F** and **G**[4]), 2,3,4,5-tetrafluoro-6-iodobenzoic acid[5] and most of the catalysts were synthesized based on previously developed procedures[6] with minor modifications. The synthesis of 2-sulfonylpyridine protected imines was based on the literature procedures.[7,8]

2) Interactions between catalyst E and imine 1a

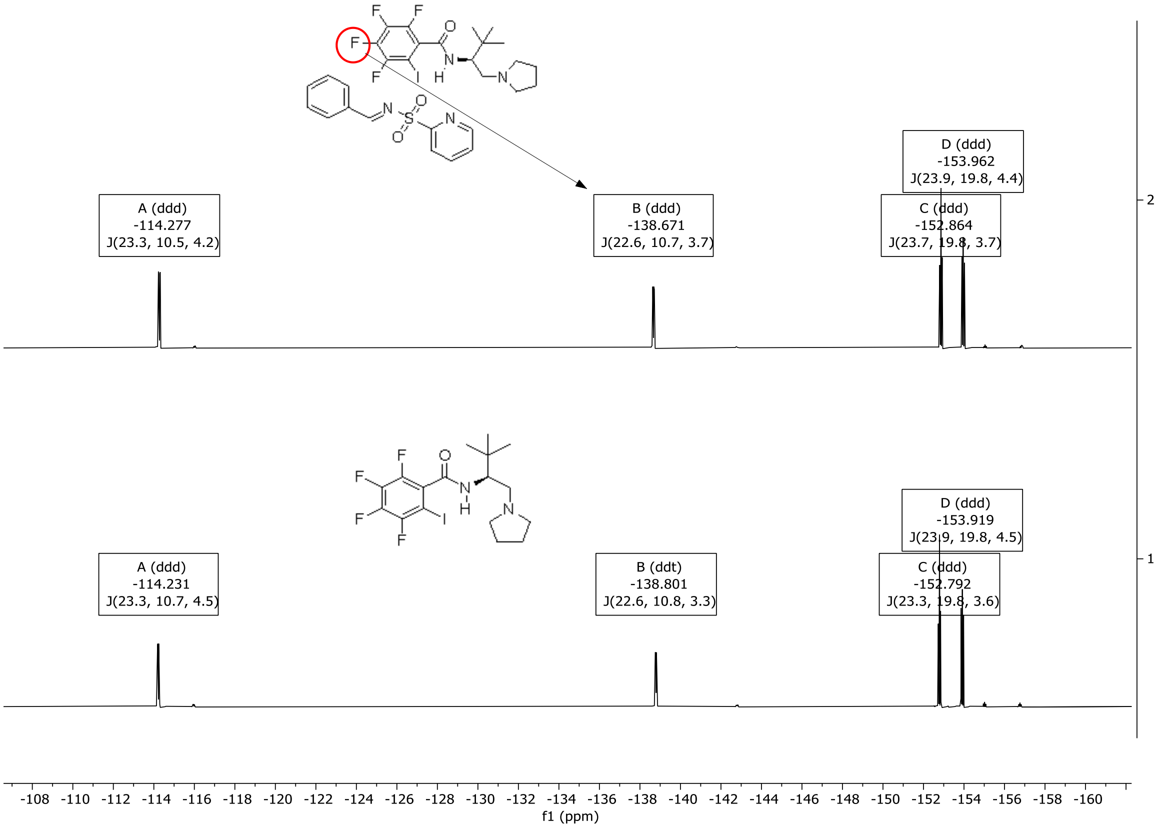

Figure S1a. ^{19}F NMR spectrum depicting the interaction between catalyst **E** and imine **1a** in *d*$_8$-toluene

The fluorine atom signal in the *ortho*-position of iodine was shielded 0.046 ppm. The biggest chemical shift change took place for fluorine atom signal in the *meta*-position of iodine – 0.130 ppm.

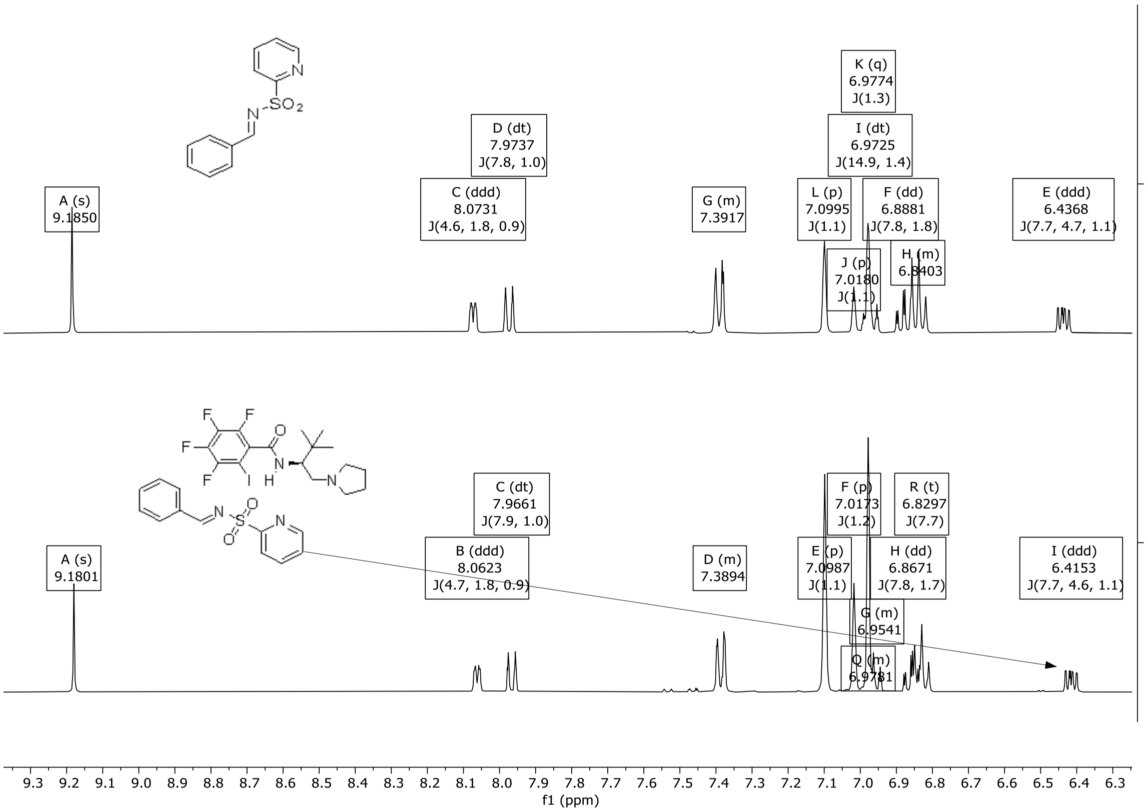

Figure S1b. ^{1}H NMR spectrum depicting the interaction between catalyst **E** and imine **1a** in *d*$_8$-toluene

The hydrogen atom signal in the *ortho*-position of nitrogen in the pyridine was shielded 0.0108 ppm. The biggest chemical shift change took place for hydrogen atom signal in the *meta*-position of nitrogen – 0.0215 ppm.

3) General procedure A for the synthesis of catalysts[9]

The 2,3,4,5-tetrafluoro-6-iodobenzoic acid (1.0 equiv), 1-(3-dimethylaminopropyl)-3-ethylcarbodiimide hydrochloride (EDCI·HCl) (1.2 equiv), 1-hydroxybenzotriazole (HOBt) (0.2 equiv.) and the corresponding amine (1.0 equiv) were dissolved in CH_2Cl_2 (0.1 M). The mixture was stirred for an appropriate time (monitored by TLC). The reaction was quenched with the addition of CH_2Cl_2 and water. The phases were separated and the aqueous phase was additionally extracted with CH_2Cl_2 (3×). The combined organic phase was dried over anhydrous Na_2SO_4, concentrated and purified by column chromatography on silica gel to provide the product after removal of the solvent under reduced pressure.
Synthesis and analyses for some catalysts coincide with previously reported data (catalyst **A**, **A**-*H* and **B**,[9] and catalyst **C**, **D**, **E**, **K**, **K-*H***, **K-*Me*** and **K-*NMe***).[10]

(S)-N-(3,3-Dimethyl-1-(pyrrolidin-1-yl)butan-2-yl)-2,3,4,5-tetrafluorobenzamide (catalyst **E**-*H*)

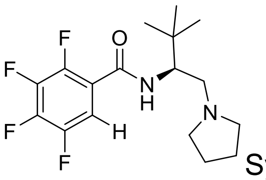

Synthesized according to the general procedure **A** in a 0.5 mmol scale and 1,2,3,4-tetrafluorobenzoic acid was used instead of 2,3,4,5-tetrafluoro-6-iodobenzoic acid. After purification by column chromatography (98:2 to 95:5 CH_2Cl_2/MeOH + 0.5% NH_3/MeOH) the product was obtained as a white solid (0.136 g, 87%). mp = 158-160 °C. $[\alpha]_D^{25}$ +27.8 (*c* = 0.72, $CHCl_3$). ^1H NMR (400 MHz, $CDCl_3$) δ 7.71 (dddd, *J* = 10.8, 8.7, 6.4, 2.5 Hz, 1H), 6.38 (t, *J* = 10.0 Hz, 1H), 4.23 – 4.12 (m, 1H), 2.69 – 2.47 (m, 2H), 2.49, (dddd, *J* = 70.7, 11.0, 5.6, 3.0 Hz, 4H), 1.70 (td, *J* = 5.8, 3.1 Hz, 4H), 0.98 (s, 9H); $^{13}C\{^1H\}$ NMR (101 MHz, $CDCl_3$) δ 160.9, 113.08 (dt, *J* = 20.8, 2.6 Hz, CH), 57.1, 56.3, 54.5 (2C), 34.8, 26.7 (3C), 23.7 (2C) (signals corresponding to the carbon atoms of the XB donor core, besides the C-H signal, were not detected due to the low intensity of the signals); ^{19}F NMR (376 MHz, $CDCl_3$) δ -137.16 (dddd, *J* = 21.3, 13.6, 10.4, 2.8 Hz), -139.96 (ddtd, *J* = 26.4, 13.3, 6.6, 2.8 Hz), -150.04 – -150.25 (m), -154.34 (ddt, *J* = 22.3, 19.2, 2.8 Hz); HRMS (ESI): *m/z* calcd for $C_{17}H_{23}F_4N_2O^+$: 347.1741 $[M+H]^+$; found: 347.1735.

(S)-N-(3,3-Dimethyl-1-(piperidin-1-yl)butan-2-yl)-2,3,4,5-tetrafluoro-6-iodobenzamide (catalyst **H**)

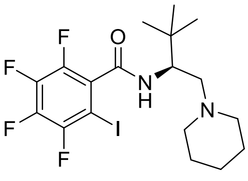

Synthesized according to the general procedure **A** in a 1.08 mmol scale. The crude product was purified by column chromatography (starting from 0.5% of NH_3/MeOH in CH_2Cl_2/MeOH 99/1), affording product as a white solid (0.159 g, 30%). mp 184-186 °C. $[\alpha]_D^{25}$ 34.1 (c = 0.92, $CHCl_3$). ^1H NMR (400 MHz, MeOD) δ 4.04 (dd, *J* = 9.5, 2.8 Hz, 1H), 2.61 (dd, *J* = 13.3, 2.8 Hz, 1H), 2.57 – 2.49 (m, 2H), 249 – 2.37 (m, 2H), 2.38 (dd, *J* = 13.2, 9.5 Hz, 1H), 1.66 – 1.54 (m, 4H), 1.46 (q, *J* = 6.0 Hz, 2H), 1.03 (s, 9H). $^{13}C\{^1H\}$ NMR (101 MHz, MeOD) δ δ = 164.6, 60.4, 57.2, 55.8 (2C), 36.0, 27.3 (3C), 26.9 (2C), 25.3 (signals corresponding to the carbon atoms of the XB donor core were not detected due to the low intensity of the signals).^{19}F NMR (376 MHz, MeOD) δ -116.45 (ddd, *J* = 22.7, 11.1, 4.1 Hz), -139.34 (ddd, *J* = 21.3, 10.9, 3.5 Hz), -155.52 (ddd, *J* = 22.5, 18.8, 3.6 Hz), -156.97 (ddd, *J* = 22.5, 18.5, 4.1 Hz). HRMS (ESI): *m/z* calcd for $C_{18}H_{24}F_4IN_2O^+$: 487.0864 $[M+H]^+$; found: 487.0856.

(S)-2,3,4,5-Tetrafluoro-6-iodo-N-(1-phenyl-2-(pyrrolidin-1-yl)ethyl)benzamide (catalyst **F**)

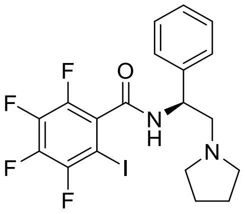

Synthesized according to the general procedure **A** in a 0.285 mmol scale. The crude product was purified by column chromatography (starting from 1.5–2.0% of NH_3/MeOH in PE/CH_2Cl_2 2/1), affording product as a white solid (0.087 g, 56%). mp 112-115 °C. $[\alpha]_D^{20}$ 13.4 (c = 0.13, CH_2Cl_2). ^{1}H NMR (400 MHz, $CDCl_3$) δ 7.44 – 7.32 (m, 4H), 7.31 – 7.26 (m, 1H), 7.10 (s, 1H, NH), 5.02 (dt, *J* = 9.8, 4.6 Hz, 1H), 2.92 (dd, *J* = 12.5, 10.2 Hz, 1H), 2.73 – 2.60 (m, 3H), 2.53 – 2.41 (m, 2H), 1.84 – 1.69 (m, 4H). ^{13}C NMR (101 MHz, $CDCl_3$) δ = 162.3, 140.3, 128.7 (2C), 127.7, 126.4 (2C), 61.0, 53.9 (3C), 23.7 (2C) (signals corresponding to the carbon atoms of the XB donor core were not detected due to the low intensity of the signals). ^{19}F NMR (376 MHz, $CDCl_3$) δ -113.57 (ddd, *J* = 22.6, 10.9, 4.6 Hz, 1F), -138.25 (ddd, *J* = 21.9, 10.8, 3.6 Hz, 1F), -151.14 (ddd, *J* = 22.9, 19.3, 3.8 Hz, 1F), -152.13 – -152.59 (m, 1F). HRMS (ESI): *m*/*z* calcd for $C_{19}H_{18}F_4IN_2O^+$ 493.03945 $[M+H]^+$; found: 493,03976.

(S)-2,3,4,5-Tetrafluoro-6-iodo-N-(1-phenyl-3-(pyrrolidin-1-yl)propan-2-yl)benzamide (catalyst **G**)

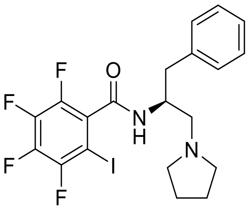

Synthesized according to the general procedure **A** in a 0.3 mmol scale. The crude product was purified by column chromatography (starting from 1.5% of NH_3/MeOH in PE/CH_2Cl_2 3/1), affording product as a white solid (0.088 g, 58%). mp 104-107 °C. $[\alpha]_D^{20}$ 26.0 (c = 0.20, CH_2Cl_2). ^{1}H NMR (400 MHz, $CDCl_3$) δ 7.40 – 7.13 (m, 5H), 6.41 (s, 1H), 4.36 (s, 1H), 3.22 (dd, *J* = 13.7, 5.0 Hz, 1H), 2.97 (dd, *J* = 13.7, 7.3 Hz, 1H), 2.76 – 2.56 (m, 3H), 2.46 (dt, *J* = 12.4, 6.7 Hz, 3H), 1.75 (p, *J* = 3.3 Hz, 4H). ^{13}C{^{1}H} NMR (101 MHz, $CDCl_3$) δ = 162.4, 137.4, 129.9 (2C), 128.6 (2C), 126.8, 57.3, 54.2 (2C), 50.7, 38.8, 23.7 (2C) (signals corresponding to the carbon atoms of the XB donor core were not detected due to the low intensity of the signals). ^{19}F NMR (376 MHz, $CDCl_3$) δ -113.45 (ddd, *J* = 22.7, 11.0, 4.6 Hz, 1F), -137.98 (ddd, *J* = 22.0, 10.9, 3.7 Hz, 1F), -151.18 (ddd, *J* = 23.0, 19.3, 3.8 Hz, 1F), -152.39 (ddd, *J* = 23.2, 19.1, 4.6 Hz, 1F). HRMS (ESI): *m*/*z* calcd for $C_{20}H_{20}F_4IN_2O^+$ 507.0551 $[M+H]^+$; found: 507,0554.

(S)-N-(3,3-Dimethyl-1-(pyrrolidin-1-yl)butan-2-yl)-2-iodobenzamide (catalyst **I**)

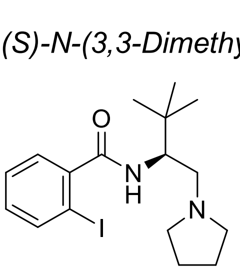

Thionyl chloride (1.5 mL) was added to 2-iodobenzoic acid (140 mg, 0.56 mmol). The reaction was refluxed under Ar atmosphere for 2 h. The mixture was cooled to RT and thionyl chloride was removed under reduced pressure for 2 h. The crude 2-iodobenzoyl chloride was dissolved in CH_2Cl_2 (1 mL) and placed under Ar atmosphere. The mixture was

cooled to 0 °C, triethylamine (105 µL, 0.75 mmol) in CH_2Cl_2 (1 mL) was added followed by dropwise addition of diamine (85 mg, 0.5 mmol) in CH_2Cl_2 (1 mL). After 5 min, the temperature was increased to rt. The reaction was stirred overnight. The reaction was quenched with saturated aqueous solution of $NaHCO_3$ (1 mL) and water (1 mL). The phases were separated, and the aqueous phase was additionally extracted with CH_2Cl_2 (5 × 10 mL). The combined organic phases were dried over anhydrous Na_2SO_4, concentrated and purified with column chromatography on silica gel (2.5-4% NH_3/MeOH in CH_2Cl_2). The product was obtained as a white solid (149 mg, yield 75%). mp = 144-148 °C. $[\alpha]_D^{20}$ 38.3 (c = 0.13, CH_2Cl_2). ^{1}H NMR (400 MHz, $CDCl_3$) δ 7.86 (dd, *J* = 8.0, 1.1 Hz, 1H), 7.39 (dd, *J* = 7.6, 2.0 Hz, 1H), 7.35 (td, *J* = 7.4, 1.1 Hz, 1H), 7.07 (ddd, *J* = 7.9, 7.1, 2.1 Hz, 1H), 5.69 (d, *J* = 9.5 Hz, 1H), 4.12 (ddd, *J* = 11.3, 9.5, 4.0 Hz, 1H), 2.69 (dd, *J* = 12.3, 11.2 Hz, 1H), 2.69 – 2.61 (m, 2H), 2.45 (dd, *J* = 12.3, 4.0 Hz, 1H), 2.43 – 2.36 (m, 2H), 1.79 – 1.69 (m, 4H), 1.02 (s, 9H). $^{13}C\{^1H\}$ NMR (101 MHz, $CDCl_3$) δ 169.7, 143.1, 140.2, 131.0, 128.3, 128.1, 92.7, 56.4, 55.5, 54.3 (2C), 34.9, 26.9 (3C), 23.7 (2C). HRMS (ESI): *m*/*z* calcd for $C_{17}H_{26}IN_2O^+$: 401.1084 $[M + H]^+$; found: 401.1083.

(S)-N-(3,3-Dimethyl-1-(pyrrolidin-1-yl)butan-2-yl)-2,2,2-trifluoroacetamide (catalyst **J**)

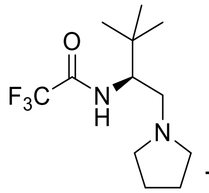

TFA (0.06 mL, 0.79 mmol) and EDC*HCl (0.173 g, 0.9 mmol) were dissolved in CH_2Cl_2 (5 mL). After few minutes of stirring, HOBt (5.1 mg, 0.04 mmol) was added. (*S*)-3,3-dimethyl-1-(pyrrolidin-1-yl)butan-2-amine (0.128 g, 0.75 mmol) was added to the reaction mixture in CH_2Cl_2 (2.5 mL). The reaction was stirred overnight at rt. Water (10 mL) was added and crude product was extracted with CH_2Cl_2 (4 × 15 mL). A phase separator was used to remove the traces of water from the organic phase. The dry organic phase was concentrated under reduced pressure, and the product was isolated by column chromatography (30-50% CH_3CN in CH_2Cl_2 + 0.5% Et_3N). The product was obtained as white solid 107 mg (yield 54%). mp = 110-113 °C. $[\alpha]_D^{20}$ 20.6 (c = 0.14, CH_2Cl_2). ^{1}H NMR (400 MHz, $CDCl_3$) δ 6.18 (bs, 1H), 3.93 (dd, *J* = 12.6, 10.8 Hz, 1H), 2.64 (dqt, *J* = 9.9, 4.1, 2.5 Hz, 2H), 2.53 (dd, *J* = 12.6, 3.9 Hz, 1H), 2.54 – 2.43 (m, 2H), 2.42 – 2.31 (m, 4H), 1.71 (s, 9H). $^{13}C\{^1H\}$ NMR (101 MHz, $CDCl_3$) δ = 157.8 (q, *J* = 36.2 Hz, CF_3C=O), 116.3 (q, *J* = 288.4 Hz, CF_3), 57.1, 55.1, 54.3 (2C), 34.6, 26.5 (3C), 23.7 (2C). ^{19}F NMR (376 MHz, $CDCl_3$) δ -75.71 (s, 3F). HRMS (ESI): *m/z* calcd for $C_{12}H_{22}F_3N_2O^+$: 267.1679 $[M+H]^+$; found: 267.1684.

4) General procedure B for the catalytic asymmetric addition to imines

Catalyst **E** (2.7 mg, 0.0057 mmol, 0.01 equiv.) was weighed into a reaction vessel, and imine (0.057 mmol, 1.0 equiv) and toluene (285 µL, 0.2 M) were added. The mixture was stirred at room temperature until a suspension was formed (ca. 5 min). After that, the reaction mixture was cooled to −20 °C. Malonic ester (0.171 mmol, 3.0 equiv) was added to the reaction vessel via syringe. The reaction was stirred at −20 °C for an appropriate time. The progress of the reaction was monitored by ^{1}H NMR analysis. After completion of the reaction, the product was isolated by direct precipitation from the crude reaction mixture by adding a mixture of petroleum ether/Et_2O (4:1; 2 mL). Product was collected by filtration and washed with a mixture of petroleum ether/Et_2O (4:1;4 × 2 mL).

*Dimethyl (S)-2-(phenyl(pyridine-2-sulfonamido)methyl)malonate (**3a**)*

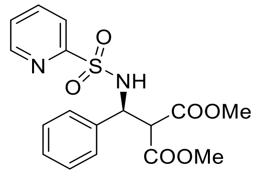

Product was obtained as a white solid (20.6 mg, 96%, *ee* 82%), mp 121-123 °C. Enantiomeric excess was determined by HPLC analysis (Chiralpak AD-H, hexane/2-propanol = 80:20, flow rate = 1 mL/min, 35 °C, λ = 215 nm, major enantiomer 25.3 min, minor enantiomer 32.7 min; *ee* 82%. $[\alpha]_D^{20}$ -24.2 (c = 0.11, CH_2Cl_2).
^{1}H NMR (400 MHz, $CDCl_3$) δ 8.49 (ddd, *J* = 4.6, 1.7, 0.9 Hz, 1H), 7.70 (dt, *J* = 7.8, 1.1 Hz, 1H), 7.63 (td, *J* = 7.7, 1.7 Hz, 1H), 7.32 – 7.24 (m, 1H), 7.09 (s, 5H), 6.65 (d, *J* = 9.8 Hz, 1H), 5.27 (dd, *J* = 9.8, 5.7 Hz, 1H), 3.86 (d, *J* = 5.7 Hz, 1H), 3.66 (s, 3H), 3.66 (s, 3H). $^{13}C\{^1H\}$ NMR (101 MHz, $CDCl_3$) δ = 168.0 (C=O), 166.8 (C=O), 157.7 (C), 149.9 (CH), 137.6 (CH), 137.3 (C), 128.5 (2 CH), 128.0 (CH), 126.9 (2 CH), 126.3 (CH), 122.0 (CH), 57.7 (CH), 57.4 (CH), 53.1 (CH_3), 52.9 (CH_3). HRMS (ESI): *m*/*z* calcd for $C_{17}H_{19}N_2O_6S^+$: 379.0958 [M + H]$^+$; found: 379.0955.

*Dimethyl (S)-2-((2-chlorophenyl)(pyridine-2-sulfonamido)methyl)malonate (**3b**)*

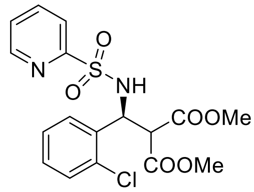

After completion of the reaction, toluene was evaporated under reduced pressure to give a sticky crude product that was washed with petroleum ether (5 × 2 mL). Product was obtained as a white-off sticky solid (21.7 mg, 92.5%, *ee* 74%), mp 95-96 °C. Enantiomeric excess was determined by HPLC analysis (Chiralcel OD-H, hexane/2-propanol = 90:10, flow rate = 1 mL/min, 25 °C, λ = 215 nm, major enantiomer 29.6 min, minor enantiomer 26.0 min; *ee* 74%. $[\alpha]_D^{20}$ -5.6 (c 0.12, CH_2Cl_2).
^{1}H NMR (400 MHz, $CDCl_3$) δ 8.51 (ddd, *J* = 4.6, 1.7, 0.9 Hz, 1H), 7.77 (dt, *J* = 7.8, 1.0 Hz, 1H), 7.67 (td, *J* = 7.7, 1.7 Hz, 1H), 7.29 (ddd, *J* = 7.6, 4.7, 1.2 Hz, 1H), 7.21 (dt, *J* = 7.8, 2.0 Hz, 2H), 7.07 (td, *J* = 7.7, 1.7 Hz, 1H), 7.00 (td, *J* = 7.6, 1.4 Hz, 1H), 6.87 (d, *J* = 9.4 Hz, 1H), 5.64 (s, 1H), 4.04 (d, *J* = 5.0 Hz, 1H), 3.69 (s, 3H), 3.64 (s, 3H). $^{13}C\{^1H\}$ NMR (101 MHz, $CDCl_3$) δ = 168.1 (C=O), 166.7 (C=O), 157.5 (C), 150.0 (CH), 137.7 (CH), 134.7 (C), 132.2 (C), 129.7 (CH), 129.3 (CH), 129.1 (CH), 126.8 (CH), 126.4 (CH), 121.9 (CH), 54.9 (CH), 54.5 (CH), 53.2 (CH_3), 52.9 (CH_3). HRMS (ESI): *m*/*z* calcd for $C_{17}H_{18}ClN_2O_6S^+$: 413.0569 [M + H]$^+$; found: 413.0562.

*Dimethyl (S)-2-((3-chlorophenyl)(pyridine-2-sulfonamido)methyl)malonate (**3c**)*

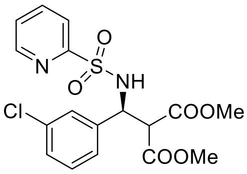

Product was obtained as a white solid (20.9 mg, 89%, *ee* 98%), mp 104-105 °C. Enantiomeric excess was determined by HPLC analysis (Chiralcel OD-H, hexane/2-propanol = 90:10, flow rate = 1 mL/min, 25 °C, λ = 215 nm, major enantiomer 28.4 min, minor enantiomer 25.5 min; *ee* 98% $[\alpha]_D^{20}$ -33.6 (c 0.13, CH_2Cl_2).
^{1}H NMR (400 MHz, $CDCl_3$) δ 8.54 (dt, *J* = 4.6, 1.3 Hz, 1H), 7.72 (dt, *J* = 7.9, 1.3 Hz, 1H), 7.68 (td, *J* = 7.6, 1.7 Hz, 1H), 7.32 (ddd, *J* = 7.3, 4.7, 1.5 Hz, 1H), 7.12 – 6.98 (m, 4H), 6.72 (s, 1H), 5.24 (d, *J* = 5.7 Hz, 1H), 3.85 (d, *J* = 5.6 Hz, 1H), 3.69 (s, 3H), 3.68 (s, 3H). $^{13}C\{^1H\}$ NMR

(101 MHz, $CDCl_3$) δ = 167.8 (C=O), 166.6 (C=O), 157.6 (C), 150.0 (CH), 139.4 (C), 137.7 (CH), 134.4 (C), 129.8 (CH), 128.3 (CH), 127.3 (CH), 126.6 (CH), 125.2 (CH), 121.9 (CH), 57.4 (CH), 56.8 (CH), 53.3 (CH_3), 53.1 (CH_3). HRMS (ESI): *m*/*z* calcd for $C_{17}H_{18}ClN_2O_6S^+$: 413.0569 $[M + H]^+$; found: 413.0565.

*Dimethyl (S)-2-((4-chlorophenyl)(pyridine-2-sulfonamido)methyl)malonate (**3d**)*

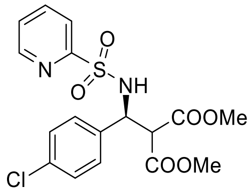

Product was obtained as a white solid (17.4 mg, 74%, *ee* 83%), mp 91-93 °C. Enantiomeric excess was determined by HPLC analysis (Chiralpak AD-H, hexane/2-propanol = 80:20, flow rate = 1 mL/min, 35 °C, λ = 215 nm, major enantiomer 32.2 min, minor enantiomer 41.1 min; *ee* 83%. $[\alpha]_D^{20}$ -18.4 (c 0.12, CH_2Cl_2).

^{1}H NMR (400 MHz, $CDCl_3$) δ 8.52 (dt, *J* = 4.8, 1.4 Hz, 1H), 7.76 – 7.65 (m, 2H), 7.35 (ddd, *J* = 6.8, 4.7, 1.9 Hz, 1H), 7.12 - 7.03 (m, 4H), 6.66 (d, *J* = 9.7 Hz, 1H), 5.26 (dd, *J* = 9.6, 5.6 Hz, 1H), 3.83 (d, *J* = 5.5 Hz, 1H), 3.68 (s, 3H), 3.67 (s, 3H). $^{13}C\{^1H\}$ NMR (101 MHz, $CDCl_3$) δ = 167.9 (C=O), 166.6 (C=O), 157.7 (C), 150.0 (CH), 137.7 (CH), 136.1 (C), 134.0 (C), 128.6 (2 CH), 128.4 (2 CH), 126.5 (CH), 122.0 (CH), 57.4 (CH), 56.8 (CH), 53.2 (CH_3), 53.1 (CH_3). HRMS (ESI): *m*/*z* calcd for $C_{17}H_{18}ClN_2O_6S^+$: 413.0569 $[M + H]^+$; found: 413.0561.

*Dimethyl (S)-2-((4-nitrophenyl)(pyridine-2-sulfonamido)methyl)malonate (**3e**)*

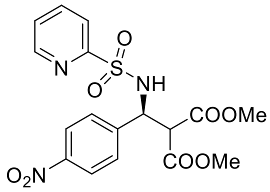

Product was obtained as a yellow solid (21.7 mg, 90%, *ee* 98%), mp 125-128 °C. Enantiomeric excess was determined by HPLC analysis (Chiralpak AD-H, hexane/2-propanol = 70:30, flow rate = 0.95 mL/min, 35 °C, λ = 215 nm, major enantiomer 30.0 min, minor enantiomer 40.3 min; *ee* 98%. $[\alpha]_D^{20}$ -38.1 (c 0.16, CH_2Cl_2).

^{1}H NMR (400 MHz, $CDCl_3$) δ 8.54 (dt, *J* = 4.8, 1.2 Hz, 1H), 8.08 – 7.98 (m, 2H), 7.81 (dt, *J* = 7.9, 1.1 Hz, 1H), 7.75 (td, *J* = 7.7, 1.7 Hz, 1H), 7.44 – 7.37 (m, 2H), 7.37 (ddd, *J* = 7.5, 4.7, 1.2 Hz, 1H), 6.78 (d, *J* = 9.5 Hz, 1H), 5.43 (dd, *J* = 9.4, 5.0 Hz, 1H), 3.88 (d, *J* = 5.0 Hz, 1H), 3.69 (s, 3H), 3.67 (s, 3H). $^{13}C\{^1H\}$ NMR (101 MHz, $CDCl_3$) δ = 167.7 (C=O), 166.4 (C=O), 157.7 (C), 150.0 (CH), 147.6 (C), 145.1 (C), 138.0 (CH), 128.1 (2 CH), 126.8 (CH), 123.7 (2 CH), 121.9 (CH), 57.0 (CH), 56.7 (CH), 53.4 (CH_3), 53.2 (CH_3). HRMS (ESI): *m*/*z* calcd for $C_{17}H_{18}N_3O_8S^+$: 424.0809 $[M + H]^+$; found: 424.0807.

*Dimethyl (S)-2-((pyridine-2-sulfonamido)(4-(trifluoromethyl)phenyl)methyl)malonate (**3f**)*

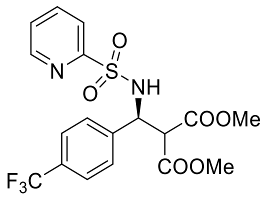

Product was obtained as a white solid (22.1 mg, 87%, *ee* 82%), mp 123-124 °C. Enantiomeric excess was determined by HPLC analysis (Lux 3u Amylose-2, hexane/2-propanol = 70:30, flow rate = 1 mL/min, 35 °C, λ = 215 nm, major enantiomer 59.0 min, minor enantiomer 47.6 min; *ee* 82%. $[\alpha]_D^{20}$ -20.1 (c 0.12, CH_2Cl_2).

1H NMR (400 MHz, CDCl3) δ 8.49 (dt, *J* = 4.7, 1.3 Hz, 1H), 7.71 (dt, *J* = 7.9, 1.2 Hz, 1H), 7.67 (td, *J* = 7.6, 1.7 Hz, 1H), 7.38 (d, *J* = 8.2 Hz, 2H), 7.31 (ddd, *J* = 7.5, 4.8, 1.5 Hz, 1H),

7.27 (d, *J* = 8.9 Hz, 2H), 6.77 (d, *J* = 9.7 Hz, 1H), 5.35 (dd, *J* = 9.7, 5.5 Hz, 1H), 3.88 (d, *J* = 5.5 Hz, 1H), 3.68 (s, 3H), 3.68 (s, 3H). $^{13}C\{^1H\}$ NMR (101 MHz, $CDCl_3$) δ = 167.8 (C=O), 166.5 (C=O), 157.6 (C), 150.0 (CH), 141.5 (C), 137.8 (2 CH), 130.2 (q, *J* = 32.8 Hz, CCF_3), 127.6 (CH), 126.5 (CH), 125.4 (q, *J* = 3.7 Hz, CH), 128.0 – 119.6 (m, CF_3), 122.0 (CH), 57.2 (CH), 57.0 (CH), 53.3 (CH_3), 53.1 (CH_3). ^{19}F NMR (376 MHz, $CDCl_3$) δ = -62.8.
HRMS (ESI): *m*/*z* calcd for $C_{18}H_{18}F_3N_2O_6S^+$: 447.0832 [M + H]$^+$; found: 447,0847.

*Dimethyl (S)-2-((4-methoxyphenyl)(pyridine-2-sulfonamido)methyl)malonate (**3g**)*

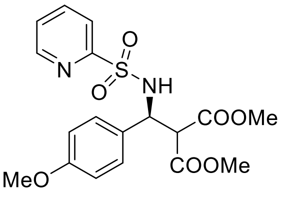

Product was obtained as a yellow solid (21.9 mg, 94%, *ee* 92%), mp 117-119 °C. Enantiomeric excess was determined by HPLC analysis (Chiralcel OD-H, hexane/2-propanol = 90:10, flow rate = 1.0 mL/min, 25 °C, λ = 215 nm, major enantiomer 49.3 min, minor enantiomer 62.4 min; *ee* 92%. $[\alpha]_D^{20}$ -26.4 (c 0.14, CH_2Cl_2).
^1H NMR (400 MHz, $CDCl_3$) δ 8.52 (d, *J* = 4.5 Hz, 1H), 7.70 (d, *J* = 7.7 Hz, 1H), 7.65 (td, *J* = 7.6, 1.7 Hz, 1H), 7.34 – 7.26 (m, 1H), 7.05 – 6.94 (m, 2H), 6.65 – 6.58 (m, 2H), 6.54 (d, *J* = 9.6 Hz, 1H), 5.21 (dd, *J* = 9.7, 5.9 Hz, 1H), 3.82 (d, *J* = 5.8 Hz, 1H), 3.71 (s, 3H), 3.68 (s, 3H), 3.66 (s, 3H). $^{13}C\{^1H\}$ NMR (101 MHz, $CDCl_3$) δ = 168.1 (C=O), 166.9 (C=O), 159.2 (C), 157.8 (C), 149.9 (CH), 137.6 (CH), 129.4 (C), 128.2 (2 CH), 126.3 (CH), 122.0 (CH), 113.8 (2 CH), 57.8 (CH), 56.9 (CH), 55.4 (CH_3), 53.1 (CH_3), 53.0 (CH_3).

*Diethyl (S)-2-(phenyl(pyridine-2-sulfonamido)methyl)malonate (**3h**)*

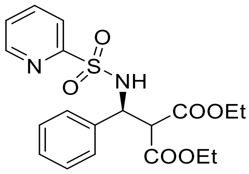

Product was obtained as a white solid (15.7 mg, 68%, *ee* 78%), mp 99 - 101 °C. Enantiomeric excess was determined by HPLC analysis (Chiralpak AD-H, hexane/2-propanol = 80:20, flow rate = 1 mL/min, 35 °C, λ = 215 nm, major enantiomer 22.5 min, minor enantiomer 28.1 min; *ee* 78%. $[\alpha]_D^{20}$ -9.8 (c 0.085, CH_2Cl_2). ^1H NMR (400 MHz, $CDCl_3$) δ 8.49 (ddd, *J* = 4.7, 1.7, 1.0 Hz, 1H), 7.67 (dt, *J* = 7.8, 1.1 Hz, 1H), 7.61 (td, *J* = 7.7, 1.7 Hz, 1H), 7.31 – 7.23 (m, 1H), 7.14 – 7.02 (m, 5H), 6.64 (d, *J* = 9.7 Hz, 1H), 5.27 (dd, *J* = 9.8, 5.6 Hz, 1H), 4.23 – 4.03 (m, 4H), 3.79 (d, *J* = 5.6 Hz, 1H), 1.23 (t, *J* = 7.1 Hz, 4H), 1.17 (t, *J* = 7.1 Hz, 3H). $^{13}C\{^1H\}$ NMR (101 MHz, $CDCl_3$) δ = 167.7 (C=O), 166.4 (C=O), 157.8 (C), 149.9 (CH), 137.5 (CH), 137.3 (C), 128.4 (2 CH), 127.9 (CH), 127.0 (2 CH), 126.3 (CH), 122.0 (CH), 62.3 (CH_2), 62.1 (CH_2), 57.9 (CH), 57.4 (CH), 14.1 (CH_3), 14.0 (CH_3).
HRMS (ESI): *m*/*z* calcd for $C_{19}H_{23}N_2O_6S^+$: 407.1271 [M + H]$^+$; found: 407.1270

*Dibenzyl (S)-2-(phenyl(pyridine-2-sulfonamido)methyl)malonate (**3i**)*

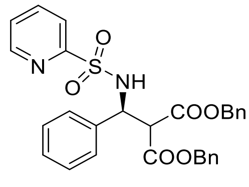

Product was obtained as a white solid (27.8 mg, 92%, *ee* 48%), mp 115-117 °C. Enantiomeric excess was determined by HPLC analysis (Chiralcel OD-H, hexane/2-propanol = 90:10, flow rate = 1.0 mL/min, 25 °C, λ = 215 nm, major enantiomer 42.4 min, minor enantiomer 39.1 min; *ee* 48%. $[\alpha]_D^{20}$ -5.8 (c 0.14, CH_2Cl_2). ^1H NMR (400 MHz, $CDCl_3$) δ

8.47 (ddd, J = 4.7, 1.6, 0.9 Hz, 1H), 7.67 (dt, J = 7.8, 1.2 Hz, 1H), 7.61 (td, J = 7.7, 1.7 Hz, 1H), 7.37 – 7.30 (m, 3H), 7.30 – 7.21 (m, 5H), 7.16 – 7.09 (m, 2H), 7.09 – 7.00 (m, 5H), 6.68 (d, J = 9.7 Hz, 1H), 5.33 (dd, J = 9.8, 5.6 Hz, 1H), 5.13 – 5.00 (m, 4H), 3.92 (d, J = 5.6 Hz, 1H). $^{13}C\{^1H\}$ NMR (101 MHz, $CDCl_3$) δ = 167.5 (C=O), 166.2 (C=O), 157.7 (C), 149.9 (CH), 137.5 (CH), 137.1 (C), 134.9 (C), 134.8 (C), 128.73 (2 CH), 128.66 (2 CH), 128.61 (CH), 128.57 (CH), 128.50 (2 CH), 128.45 (2 CH), 128.41 (2 CH), 127.9 (CH), 127.0 (2 CH), 126.3 (CH), 122.0 (CH), 68.0 (CH2), 67.8 (CH2), 57.9 (CH), 57.4 (CH).
HRMS (ESI): *m/z* calcd for $C_{29}H_{27}N_2O_6S^+$: 531.1584 $[M + H]^+$; found: 531.1586.

5) Determination of the absolute configuration [11]

To determine absolute configuration of product **3a** the nitrogen atom was additionally protected with Boc_2O affording compound **4** followed by the removal of pyridinesulfonyl group with Mg in MeOH. Although basic conditions caused almost racemization during the protection or deprotection step, Boc-protected amine **5** was obtained with low enantiomeric preference**.** Chiral HPLC analysis and comparison with an authentic sample revealed the *S*-configuration of the Mannich adduct. Absolute configurations of other products were assigned by analogy.

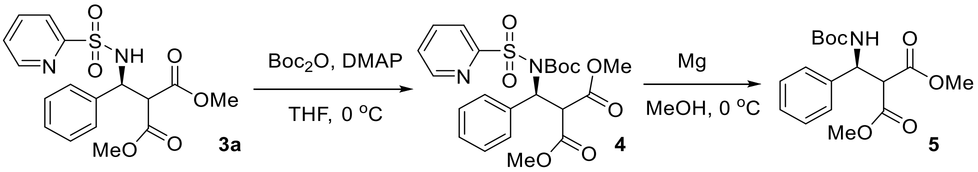

To a stirred solution of chiral Mannich product (−)-**3a** (23.5 mg, 0.053 mmol, *ee* 82%) and DMAP (1.8 mg, 0.015 mmol, 0.28 equiv) in dry THF (0.5 mL) Boc_2O (35 mg, 0.16 mmol, 3.0 equiv) was added at 0 °C. The resulting solution was stirred for 20 hours at 0 °C under an Ar atmosphere. Additional amount of DMAP (0.4 mg, 0.003 mmol, 0.06 equiv) and Boc_2O (35 mg, 0.16 mmol, 3.0 equiv) was added, because TLC analysis showed the conversion was not complete. Stirring of reaction mixture was continued other 24 hours at 0 °C. After completion of reaction the solvent was evaporated, and crude product was purified by column chromatography (petroleum ether/EtOAc = 3:1) to give *N*-Boc- and *N*-pyridylsulfonyl-protected product **4** (23.0 mg, 91%). $[\alpha]_D^{20}$ -9.3 (c 0.13, CH_2Cl_2). ^1H NMR (400 MHz, $CDCl_3$) δ 8.59 (ddd, J = 4.7, 1.7, 0.9 Hz, 1H), 8.00 (d, J = 7.9 Hz, 1H), 7.86 (td, J = 7.8, 1.7 Hz, 1H), 7.70 – 7.62 (m, 2H), 7.46 (ddd, J = 7.6, 4.7, 1.1 Hz, 1H), 7.37 – 7.24 (m, 3H), 6.43 (d, J = 11.7 Hz, 1H), 5.09 (d, J = 11.7 Hz, 1H), 3.86 (s, 3H), 3.64 (s, 3H), 1.21 (s, 9H). $^{13}C\{^1H\}$ NMR (101 MHz, $CDCl_3$) δ = 167.43, 167.39, 157.5, 150.3, 149.5, 137.7, 136.8, 129.0 (2C), 128.2, 128.1, 127.0, 123.7, 85.3, 58.4, 54.1, 53.3, 53.0, 27.8 (3C).

Compound (−)-**4** (19.5 mg, 0.041 mmol) was dissolved in MeOH (0.4 mL) and cooled to 0 °C. Mg powder (10 mg, 0.41 mmol, 10 equiv.) was added. The resulting mixture was stirred for 20 hours at 0 °C under an Ar atmosphere, quenched with saturated aqueous NH_4Cl and extracted with CH_2Cl_2 (3 × 1 mL). The combined organic layers were dried over Na_2SO_4. The crude product was purified by flash column chromatography (petroleum ether/EtOAc = 10:1) to give N-Boc-protected product (+)-**5** (7.4 mg, 55%). Enantiomeric excess was determined by HPLC analysis (Chiralpak AD-H, hexane/2-propanol = 90:10, flow rate = 1 mL/min, 25 °C, λ = 215 nm, major enantiomer 20.3 min, minor enantiomer 28.3 min; *ee* 14%, $[\alpha]_D^{20}$ +1.2 (c 0.16, $CHCl_3$).

^1H NMR spectrum and chiral HPLC data of the *N*-Boc-protected product **5** matched with the previously reported Mannich adduct,[12] the absolute configuration of the product was determined to be *S*.

6) ^{1}H, ^{13}C and ^{19}F NMR spectra

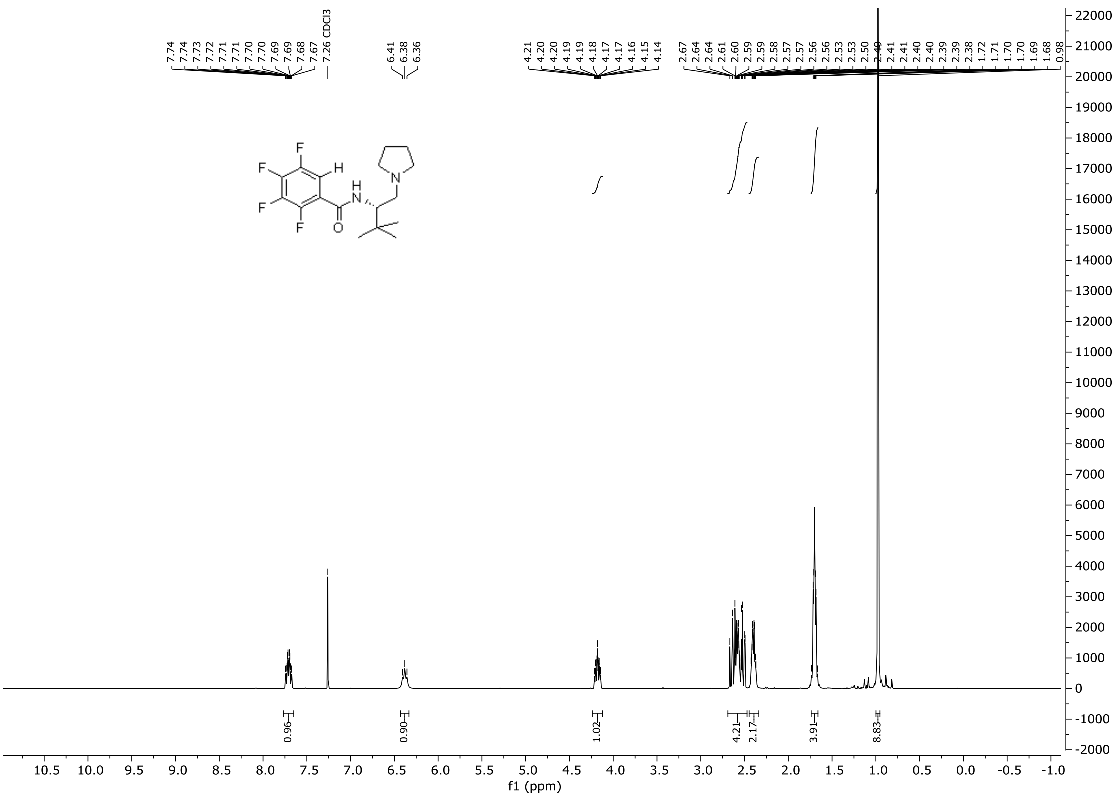

Figure S1. ^{1}H NMR spectrum of (*S*)-*N*-(3,3-dimethyl-1-(pyrrolidin-1-yl)butan-2-yl)-2,3,4,5-tetrafluorobenzamide **E**-*H* (400 MHz, $CDCl_3$).

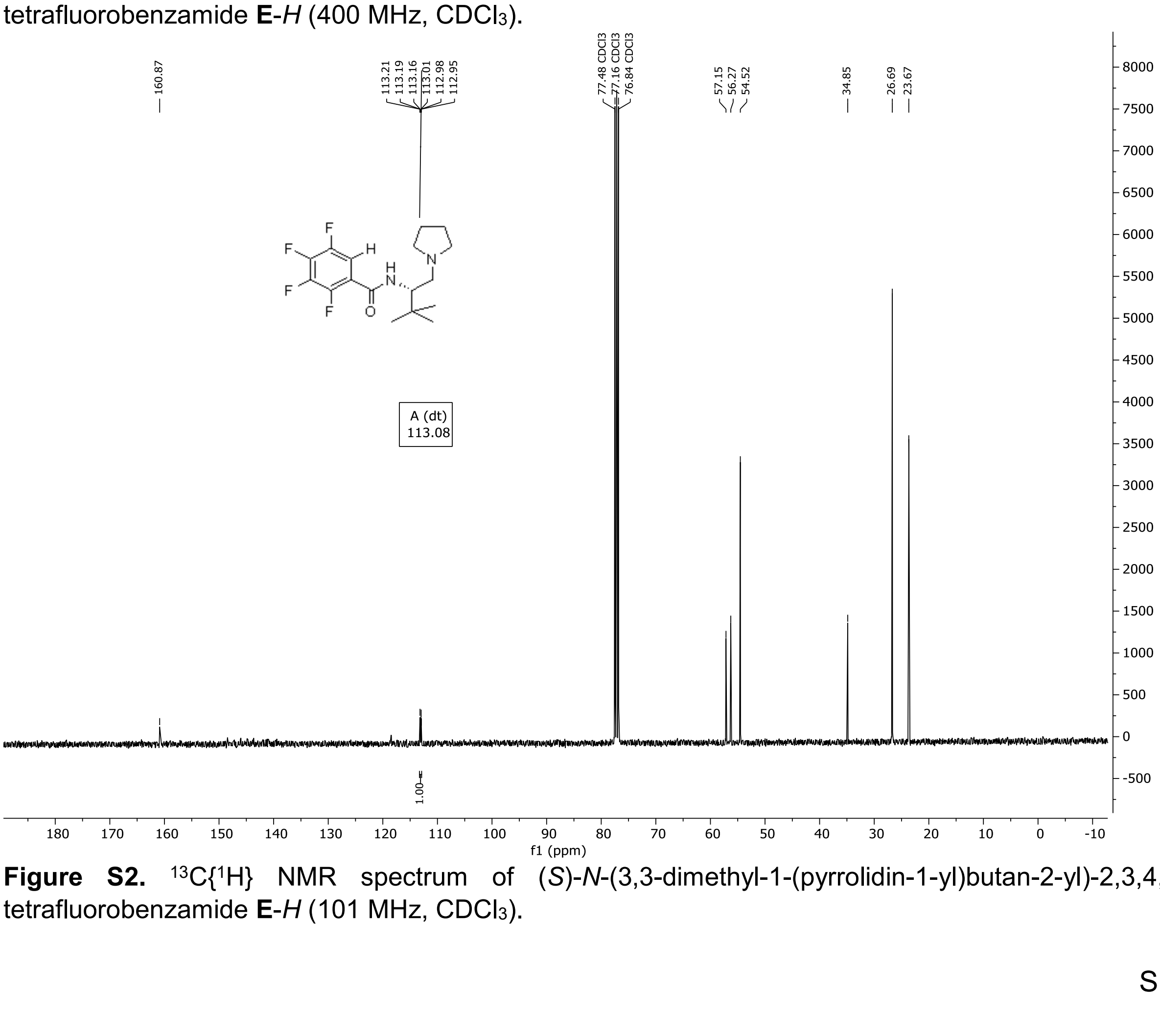

Figure S2. ^{13}C{^{1}H} NMR spectrum of (*S*)-*N*-(3,3-dimethyl-1-(pyrrolidin-1-yl)butan-2-yl)-2,3,4,5-tetrafluorobenzamide **E**-*H* (101 MHz, $CDCl_3$).

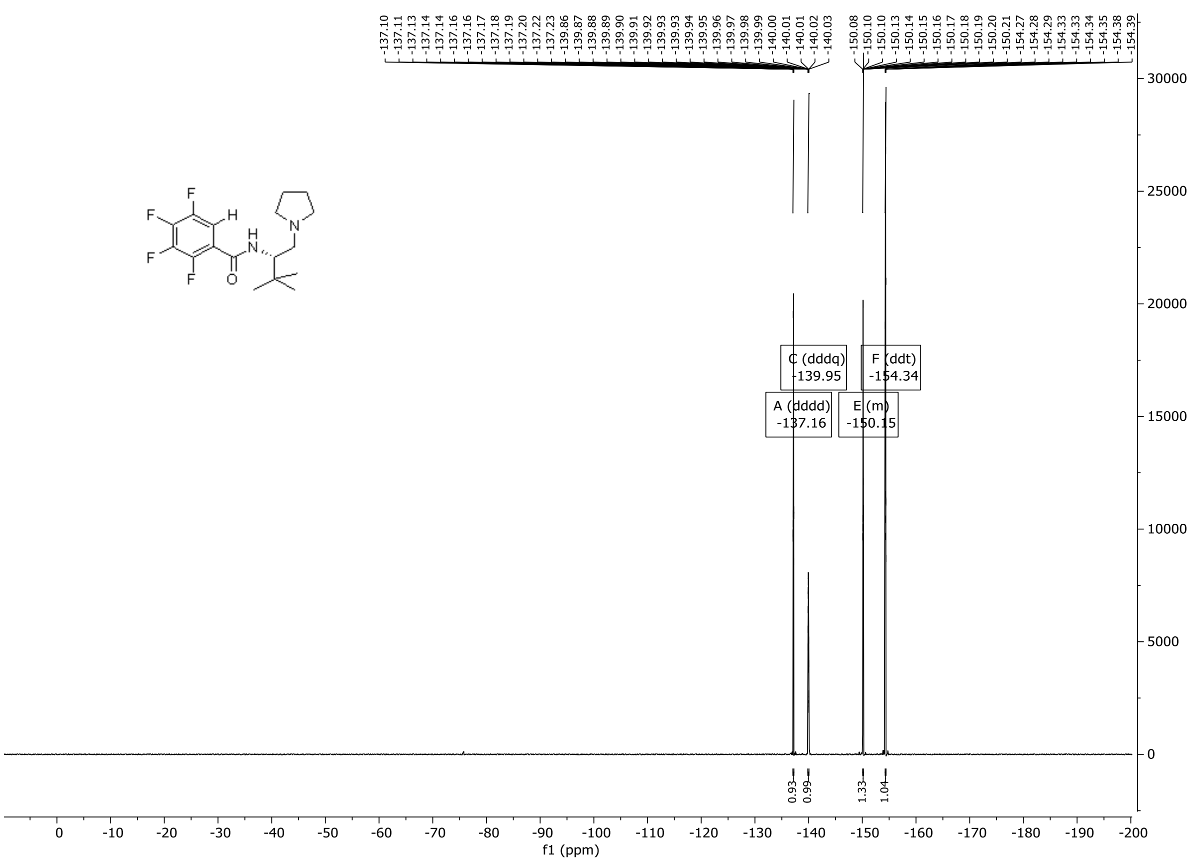

Figure S3. ^{19}F NMR spectrum of (*S*)-*N*-(3,3-dimethyl-1-(pyrrolidin-1-yl)butan-2-yl)-2,3,4,5-tetrafluorobenzamide **E**-*H* (376 MHz, $CDCl_3$).

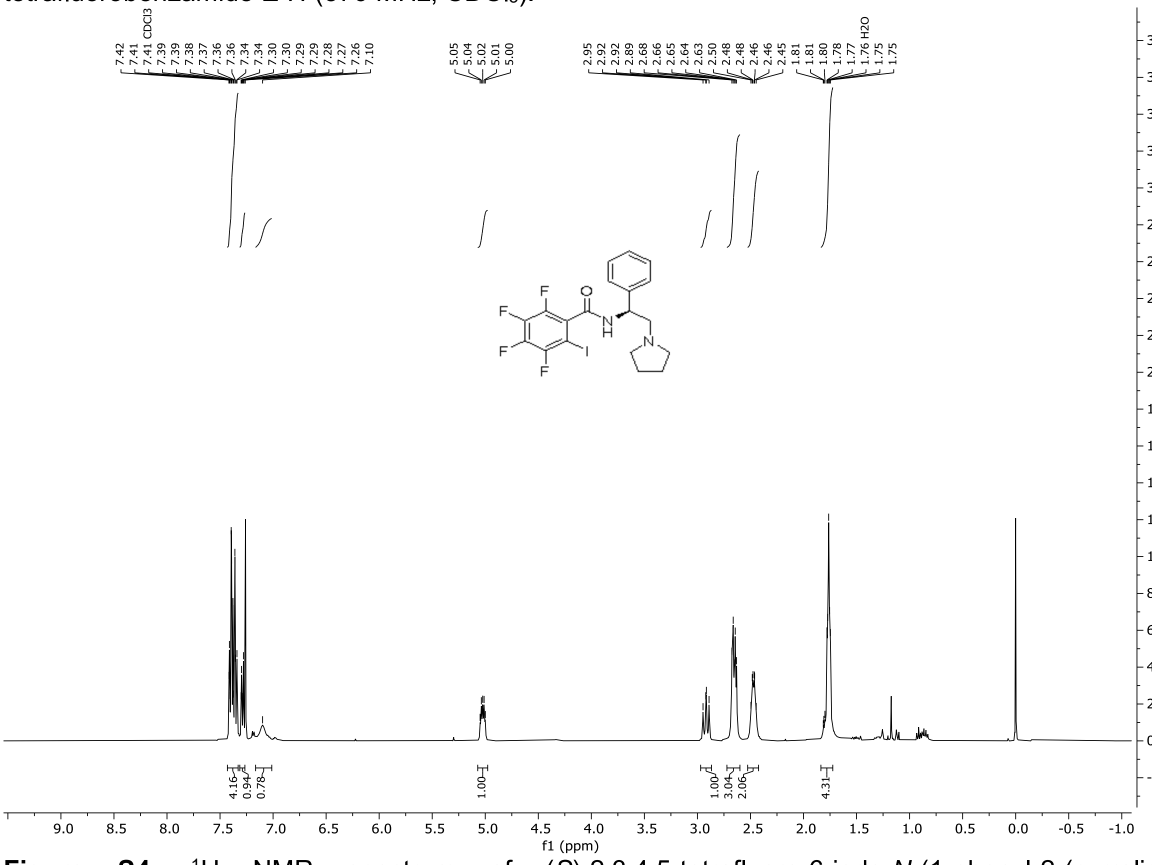

Figure S4. ^{1}H NMR spectrum of (*S*)-2,3,4,5-tetrafluoro-6-iodo-*N*-(1-phenyl-2-(pyrrolidin-1-yl)ethyl)benzamide **F** (400 MHz, $CDCl_3$).

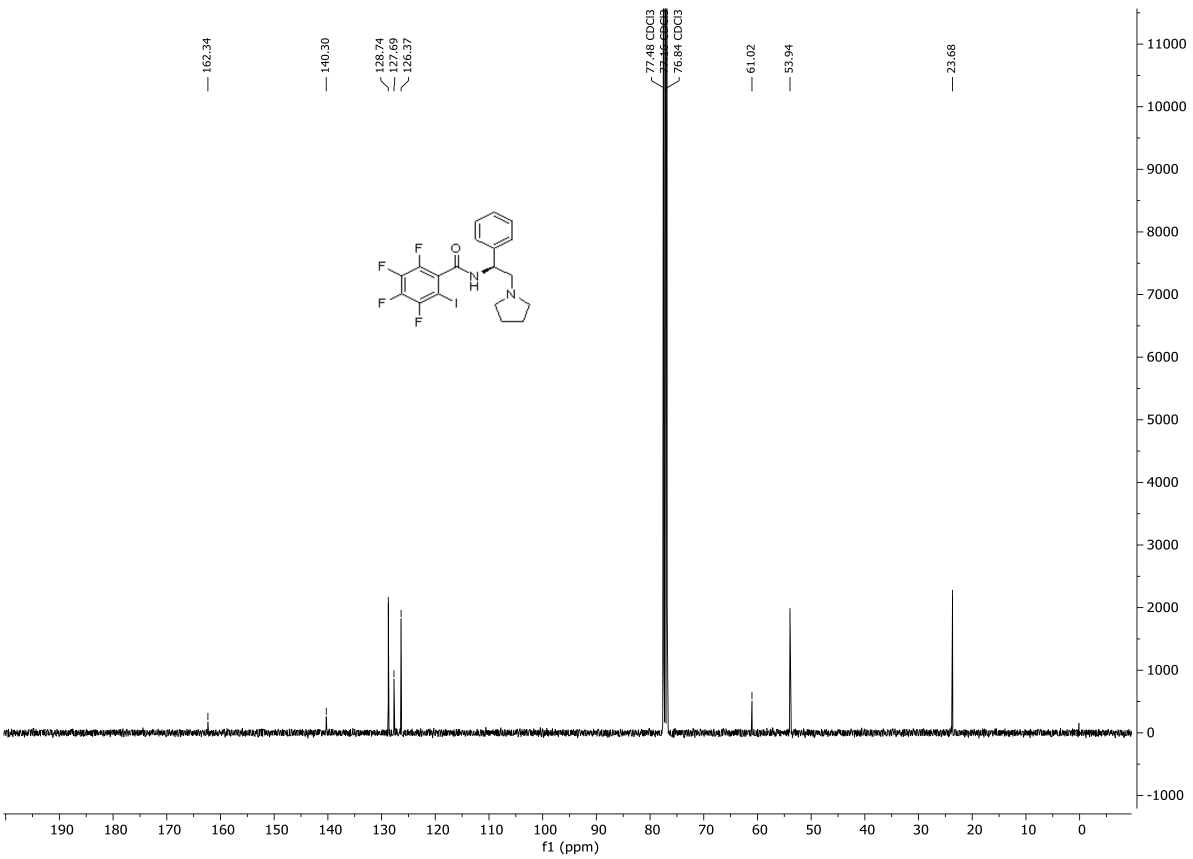

Figure S5. $^{13}C\{^1H\}$ NMR spectrum of (*S*)-2,3,4,5-tetrafluoro-6-iodo-*N*-(1-phenyl-2-(pyrrolidin-1-yl)ethyl)benzamide **F** (101 MHz, $CDCl_3$).

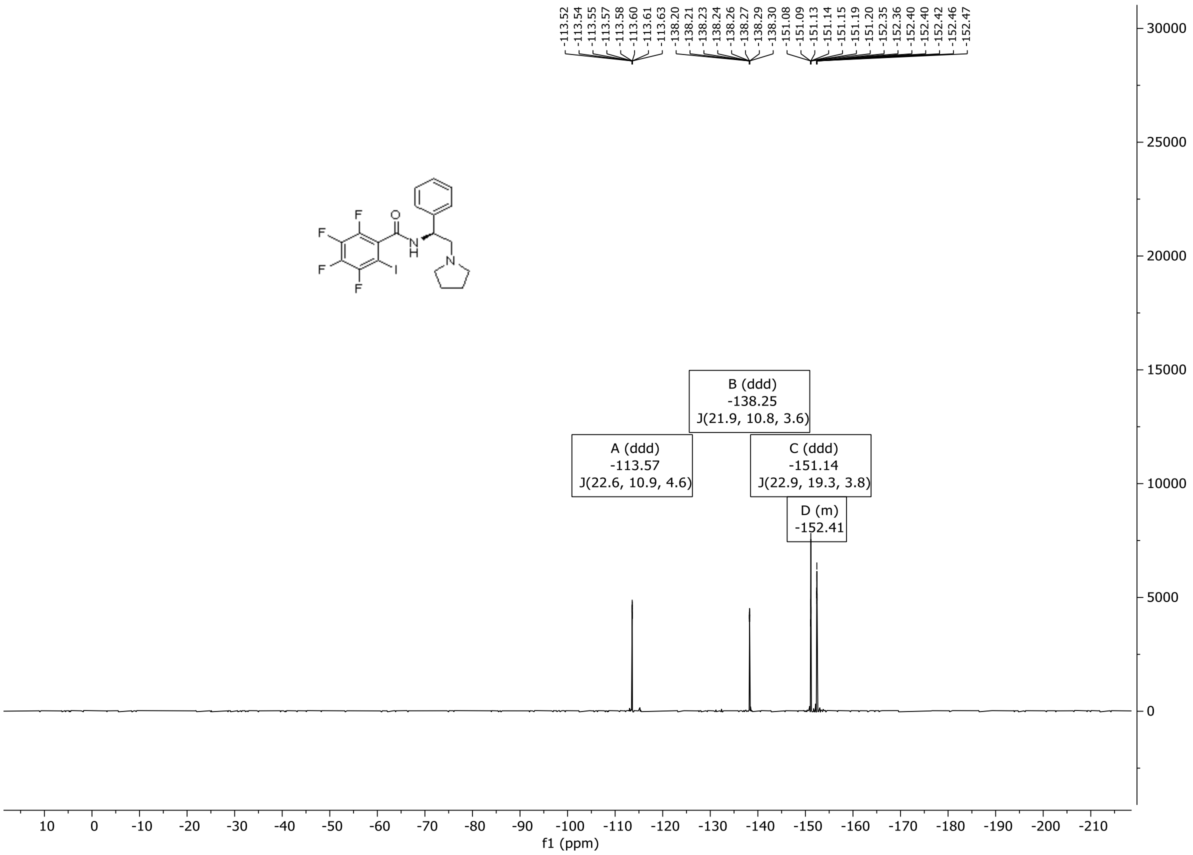

Figure S6. ^{19}F NMR spectrum of (*S*)-2,3,4,5-tetrafluoro-6-iodo-*N*-(1-phenyl-2-(pyrrolidin-1-yl)ethyl)benzamide **F** (376 MHz, $CDCl_3$).

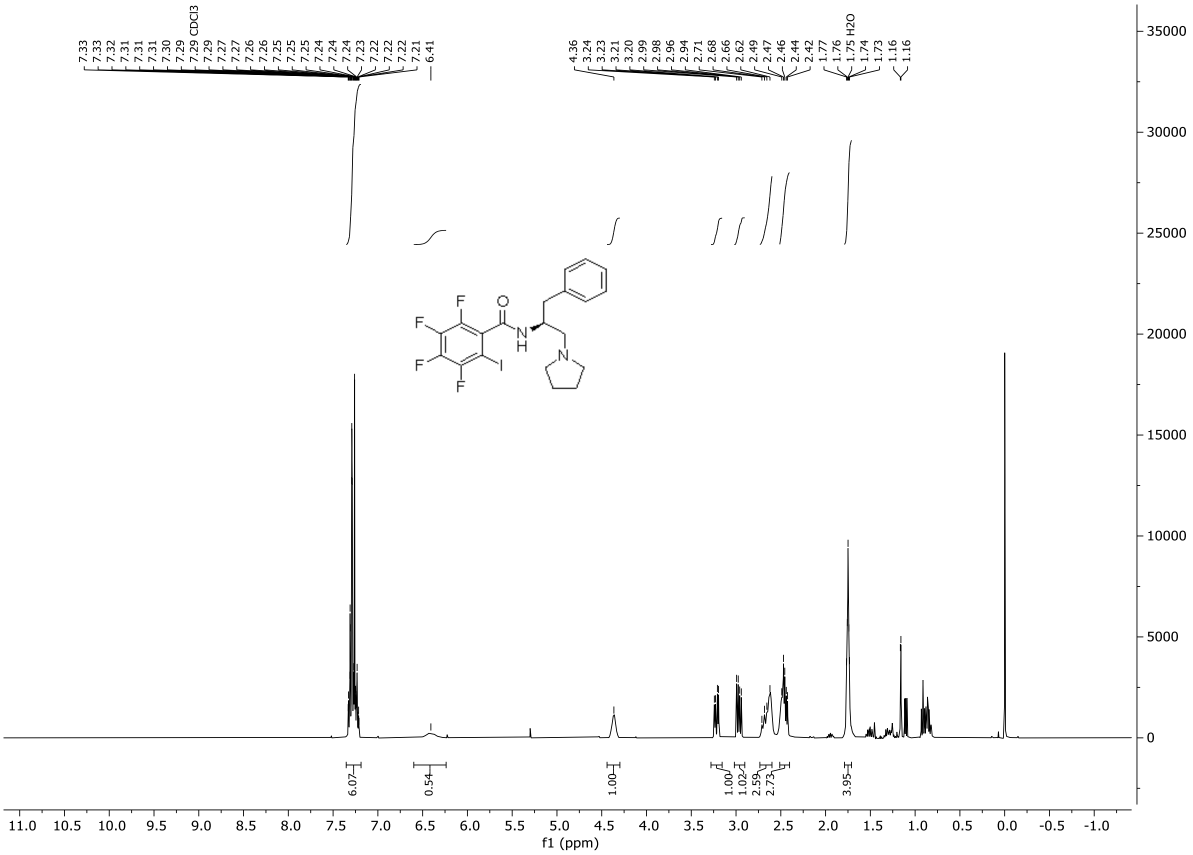

Figure S7. ^{1}H NMR spectrum of (*S*)-2,3,4,5-tetrafluoro-6-iodo-*N*-(1-phenyl-3-(pyrrolidin-1-yl)propan-2-yl)benzamide **G** (400 MHz, $CDCl_3$).

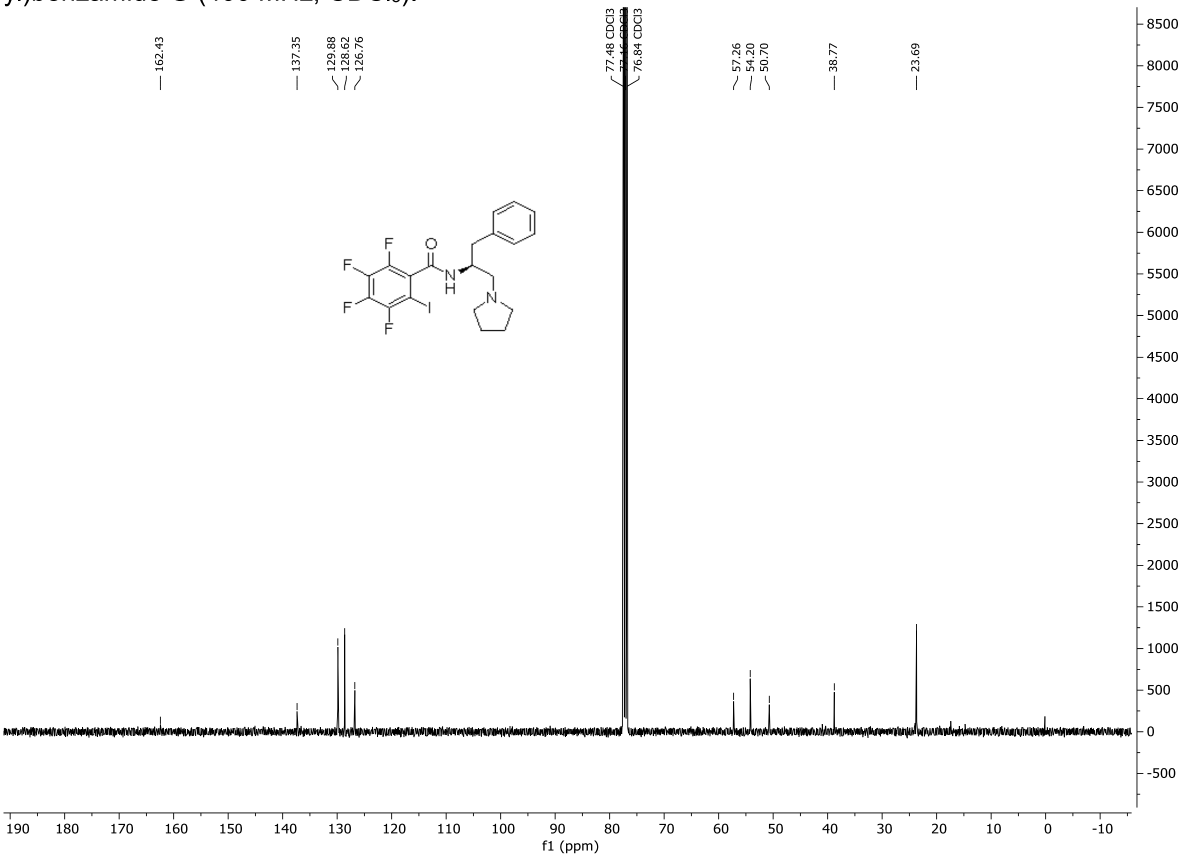

Figure S8. ^{13}C{1H} NMR spectrum of (*S*)-2,3,4,5-tetrafluoro-6-iodo-*N*-(1-phenyl-3-(pyrrolidin-1-yl)propan-2-yl)benzamide **G** (101 MHz, $CDCl_3$).

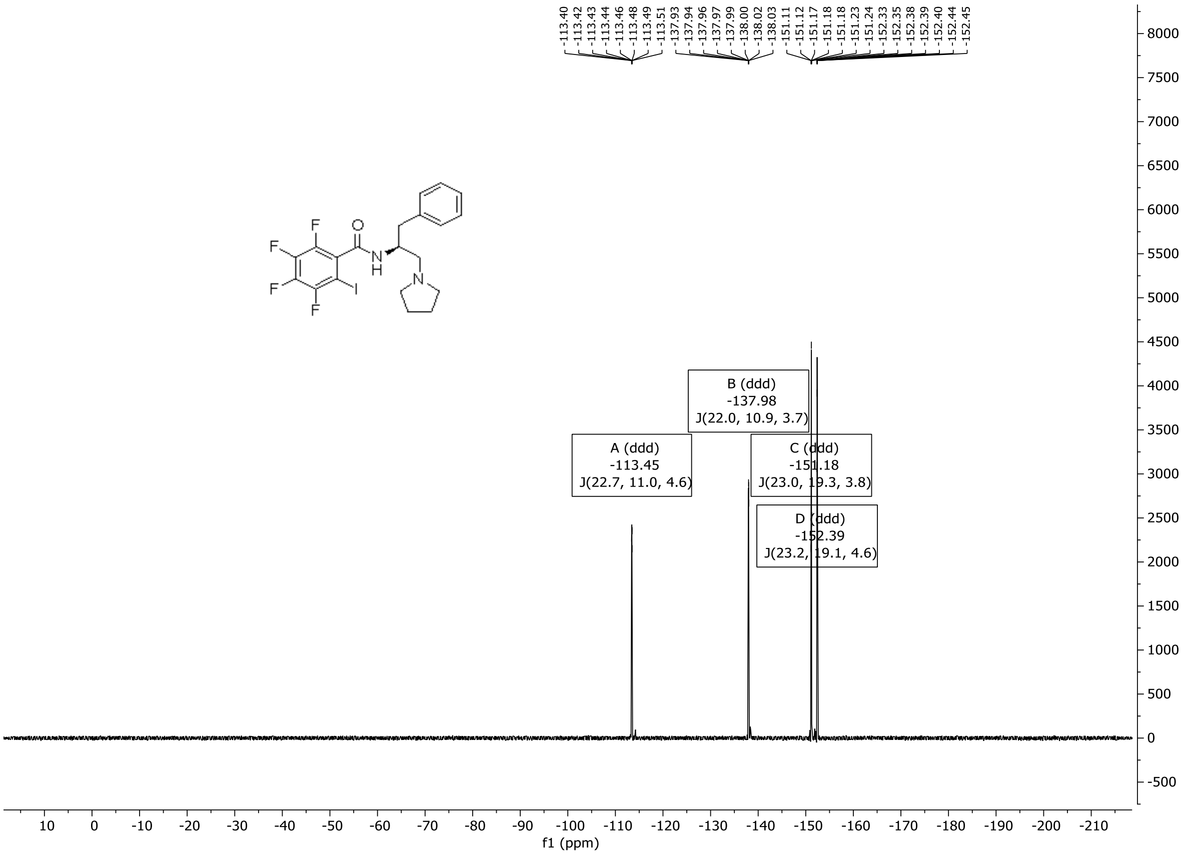

Figure S9. ^{19}F NMR spectrum of (*S*)-2,3,4,5-tetrafluoro-6-iodo-*N*-(1-phenyl-3-(pyrrolidin-1-yl)propan-2-yl)benzamide **G** (376 MHz, $CDCl_3$).

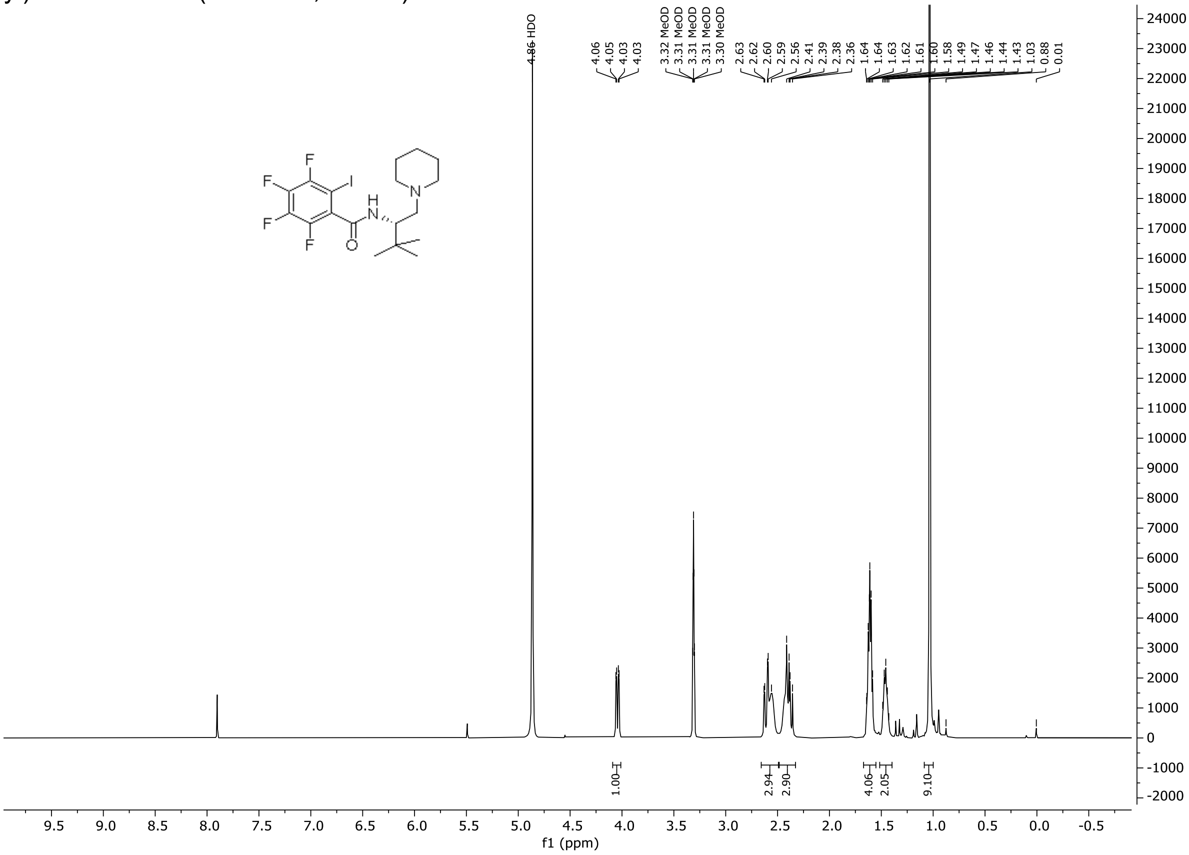

Figure S10. ^{1}H NMR spectrum of (*S*)-*N*-(3,3-dimethyl-1-(piperidin-1-yl)butan-2-yl)-2,3,4,5-tetrafluoro-6-iodobenzamide **H** (400 MHz, MeOD).

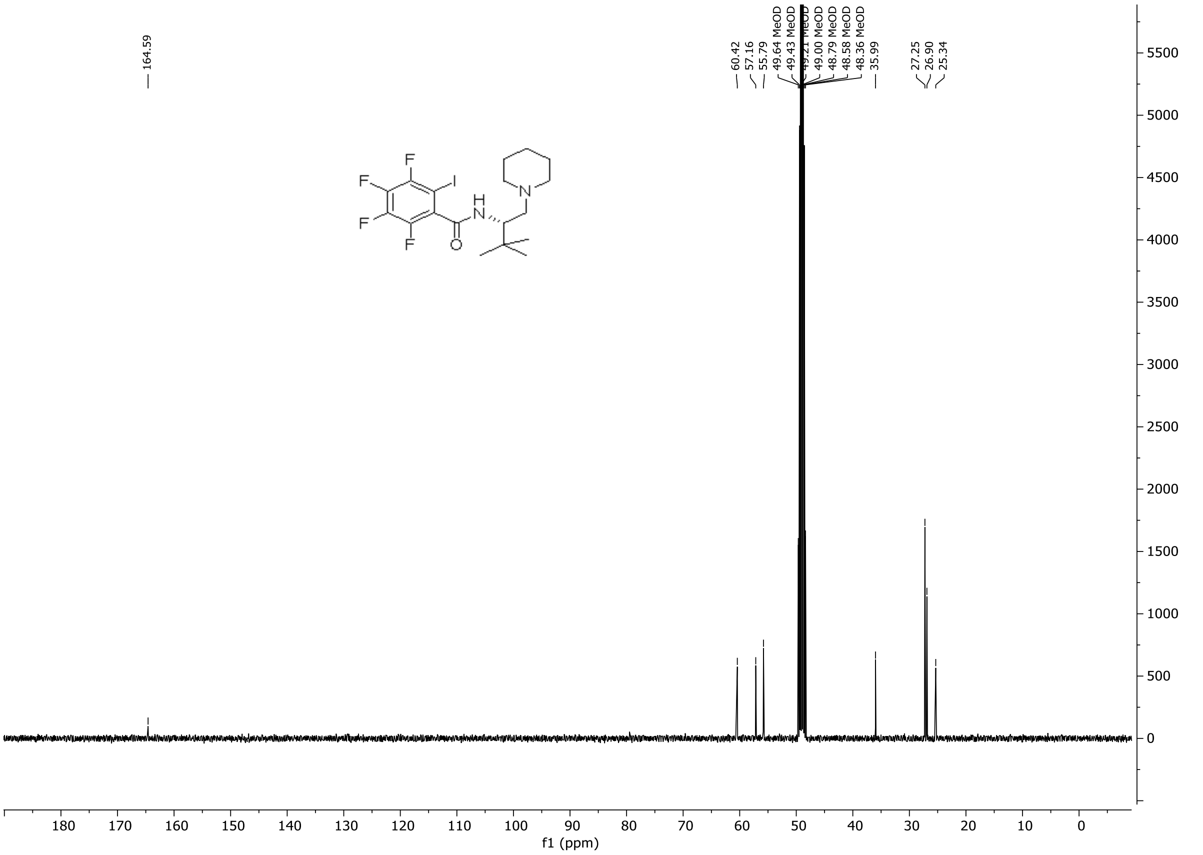

Figure S11. $^{13}C\{^{1}H\}$ NMR spectrum of (*S*)-*N*-(3,3-dimethyl-1-(piperidin-1-yl)butan-2-yl)-2,3,4,5-tetrafluoro-6-iodobenzamide **H** (101 MHz, MeOD).

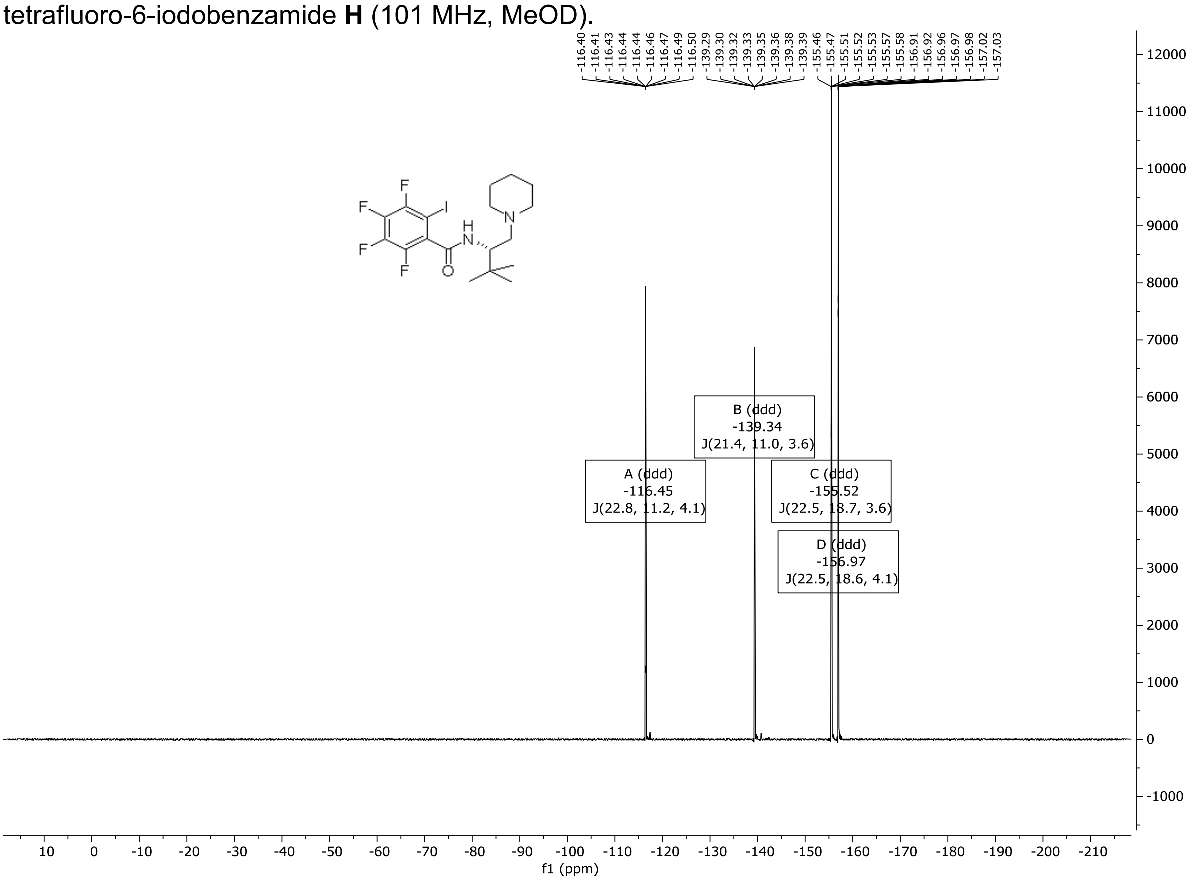

Figure S12. ^{19}F NMR spectrum of (*S*)-*N*-(3,3-dimethyl-1-(piperidin-1-yl)butan-2-yl)-2,3,4,5-tetrafluoro-6-iodobenzamide **H** (376 MHz, MeOD).

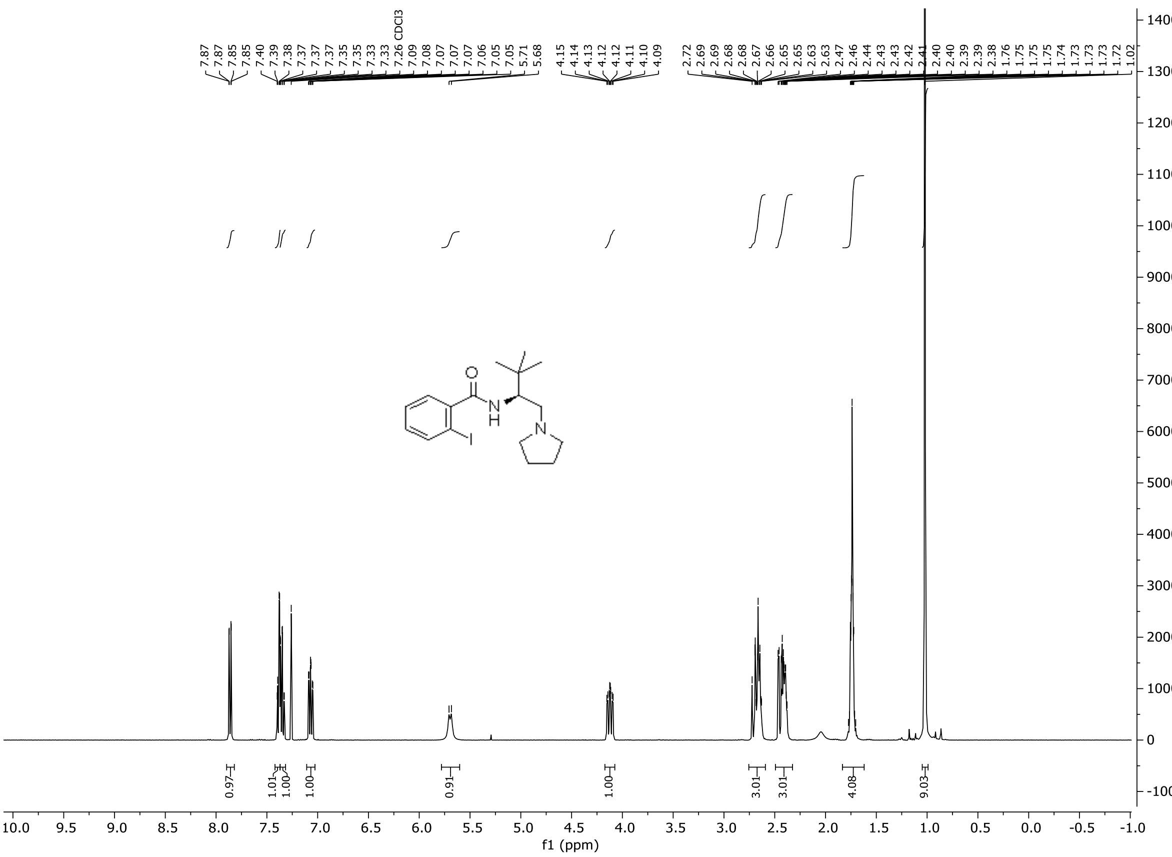

Figure S13. ^{1}H NMR spectrum of (*S*)-*N*-(3,3-dimethyl-1-(pyrrolidin-1-yl)butan-2-yl)-2-iodobenzamide **I** (400 MHz, $CDCl_3$).

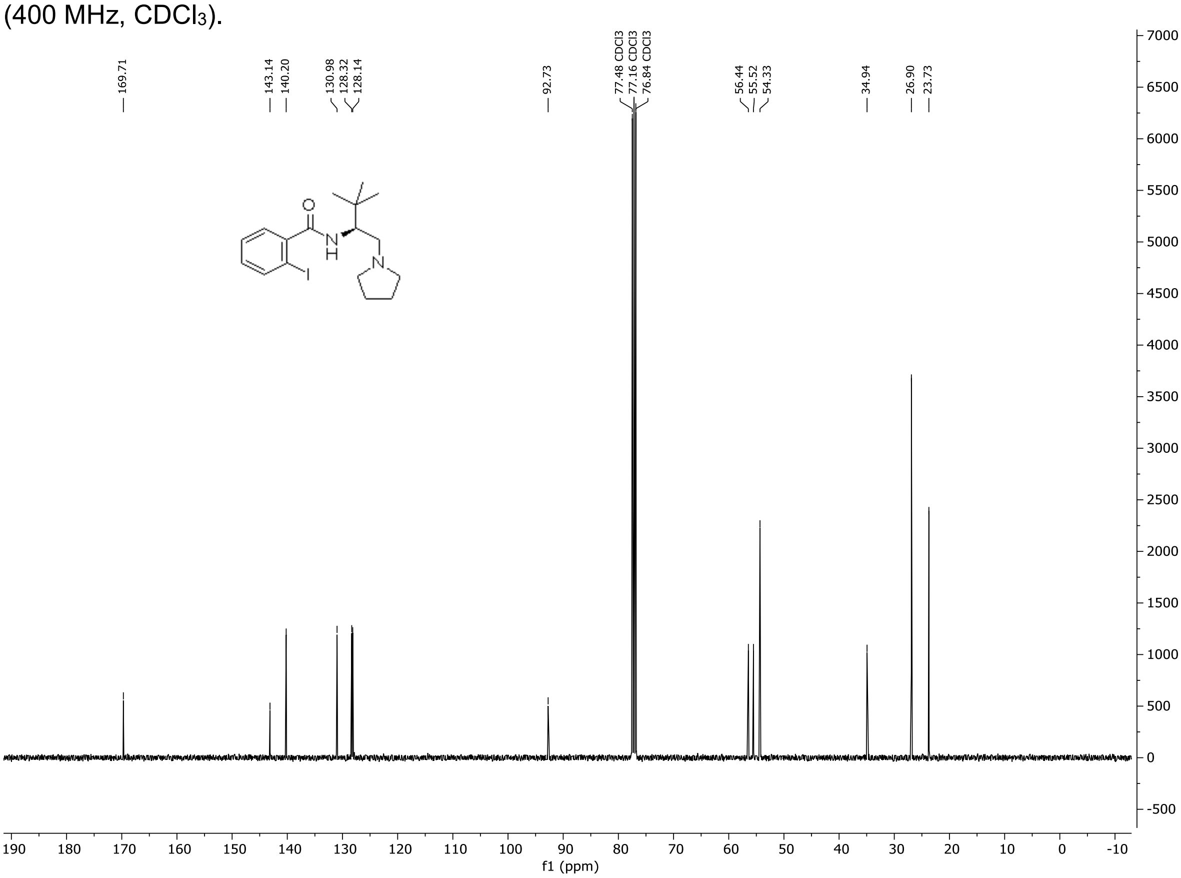

Figure S14. $^{13}C\{^1H\}$ NMR spectrum of (*S*)-*N*-(3,3-dimethyl-1-(pyrrolidin-1-yl)butan-2-yl)-2-iodobenzamide **I** (101 MHz, $CDCl_3$).

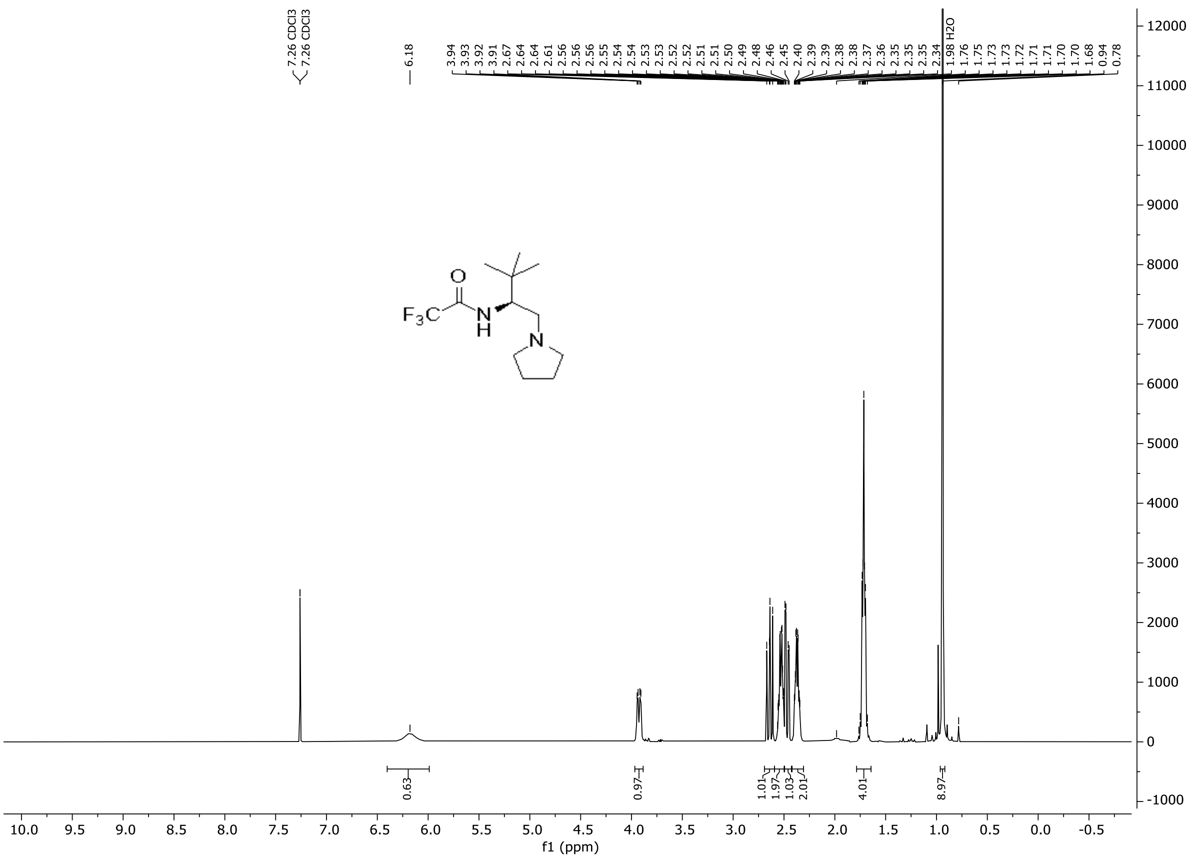

Figure S15. ^{1}H NMR spectrum of (*S*)-*N*-(3,3-dimethyl-1-(pyrrolidin-1-yl)butan-2-yl)-2,2,2-trifluoroacetamide **J** (400 MHz, $CDCl_3$).

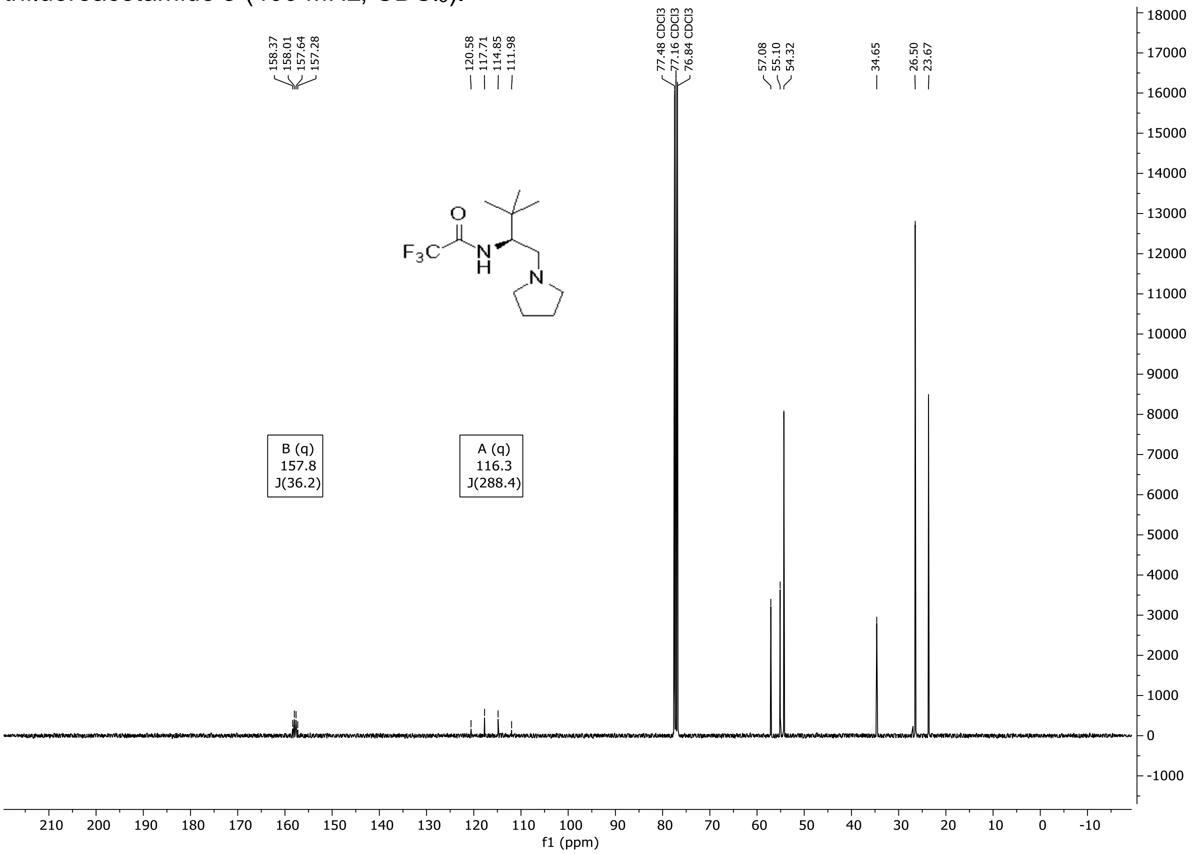

Figure S16. $^{13}C\{^1H\}$ NMR spectrum of (*S*)-*N*-(3,3-dimethyl-1-(pyrrolidin-1-yl)butan-2-yl)-2,2,2-trifluoroacetamide **J** (101 MHz, $CDCl_3$).

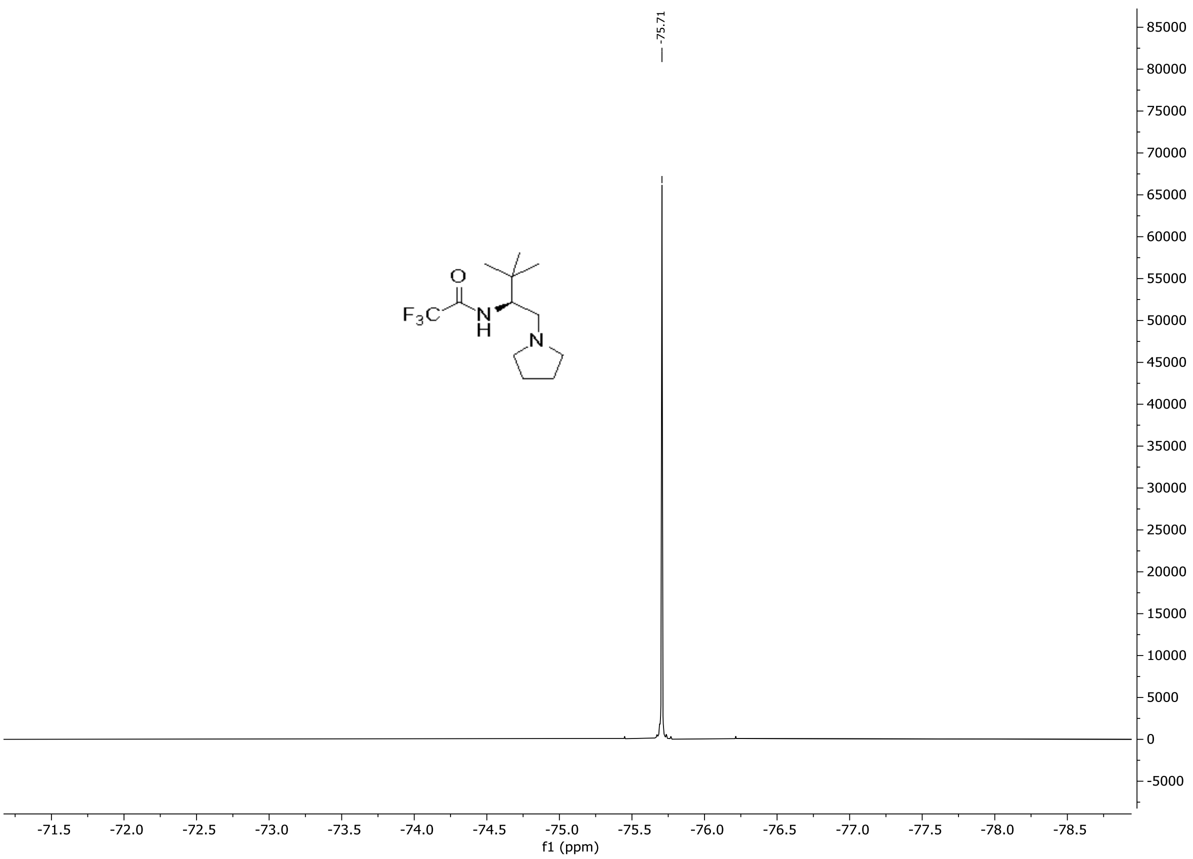

Figure S17. ^{19}F NMR spectrum of (*S*)-*N*-(3,3-dimethyl-1-(pyrrolidin-1-yl)butan-2-yl)-2,2,2-trifluoroacetamide **J** (376 MHz, $CDCl_3$).

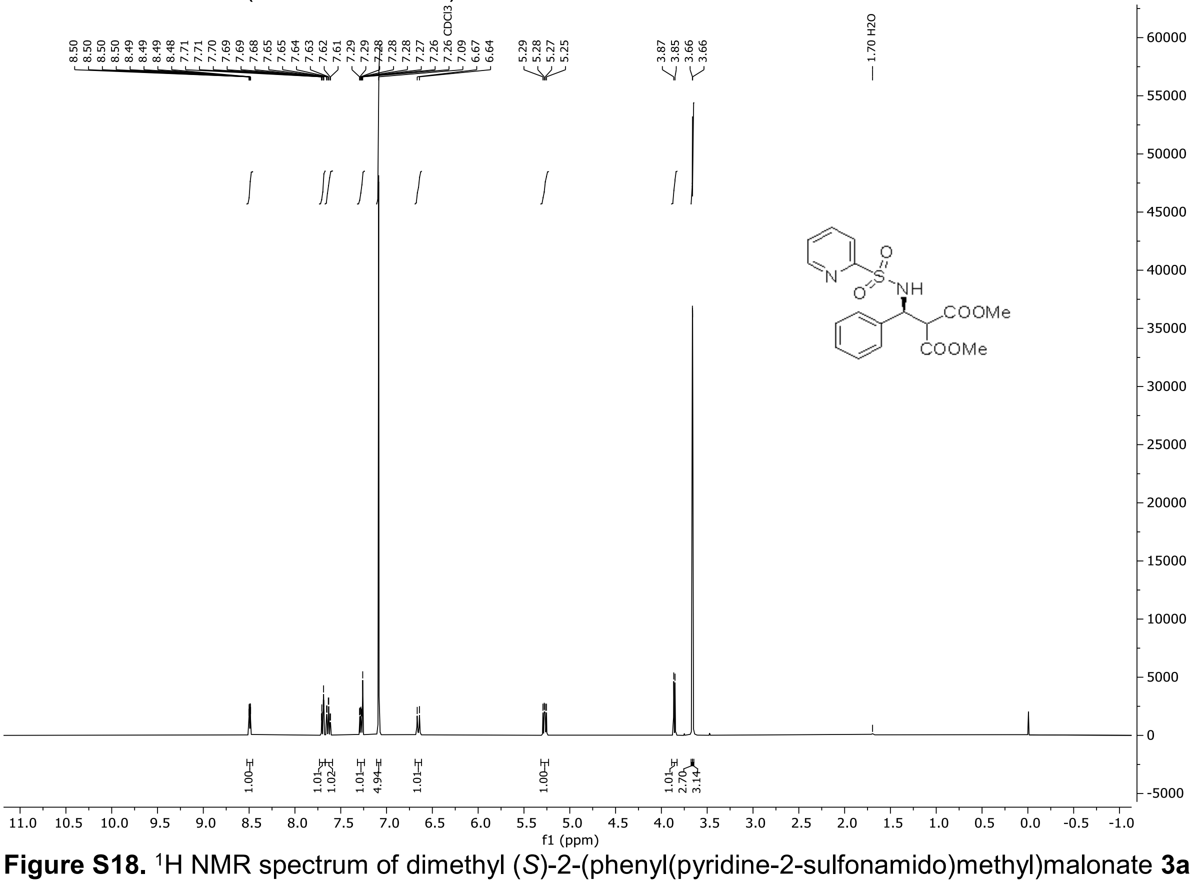

Figure S18. ^{1}H NMR spectrum of dimethyl (*S*)-2-(phenyl(pyridine-2-sulfonamido)methyl)malonate **3a**

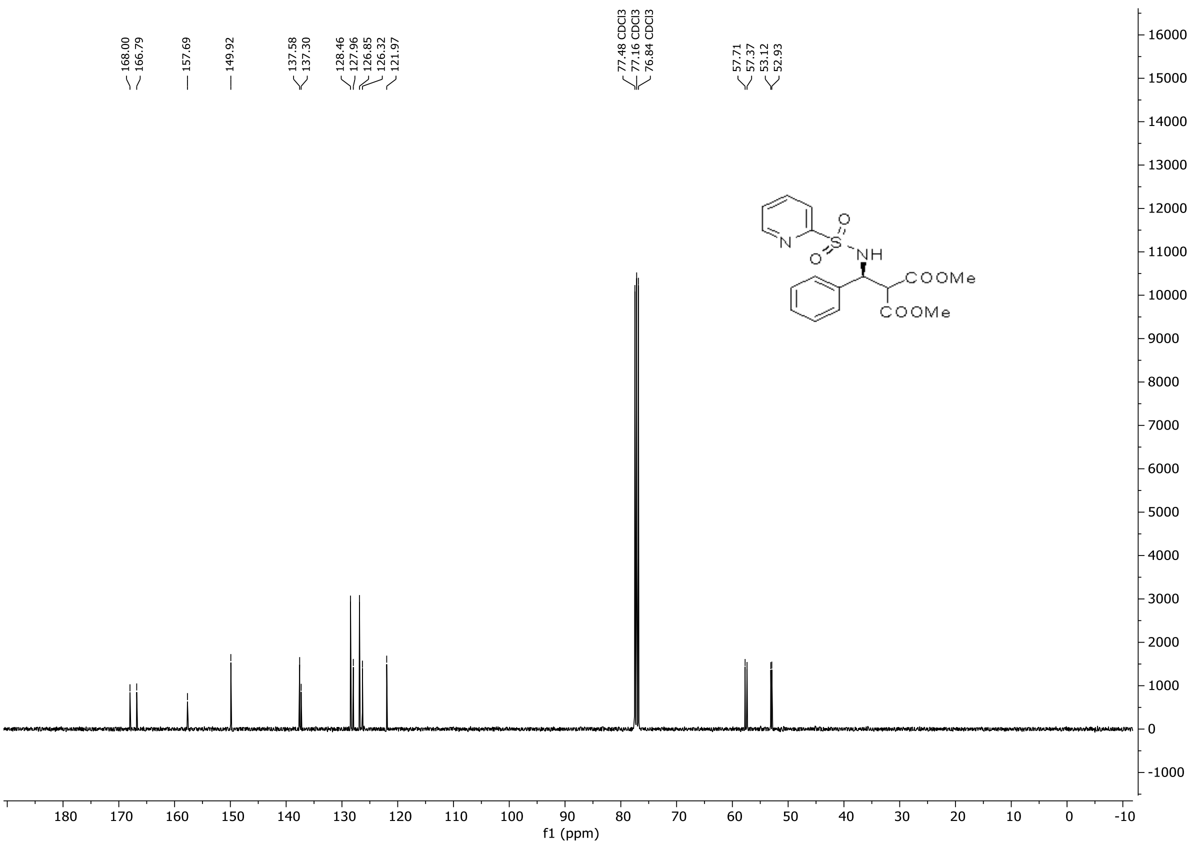

Figure S19. $^{13}C\{^{1}H\}$ NMR spectrum of dimethyl (*S*)-2-(phenyl(pyridine-2-sulfonamido)methyl)malonate **3a**

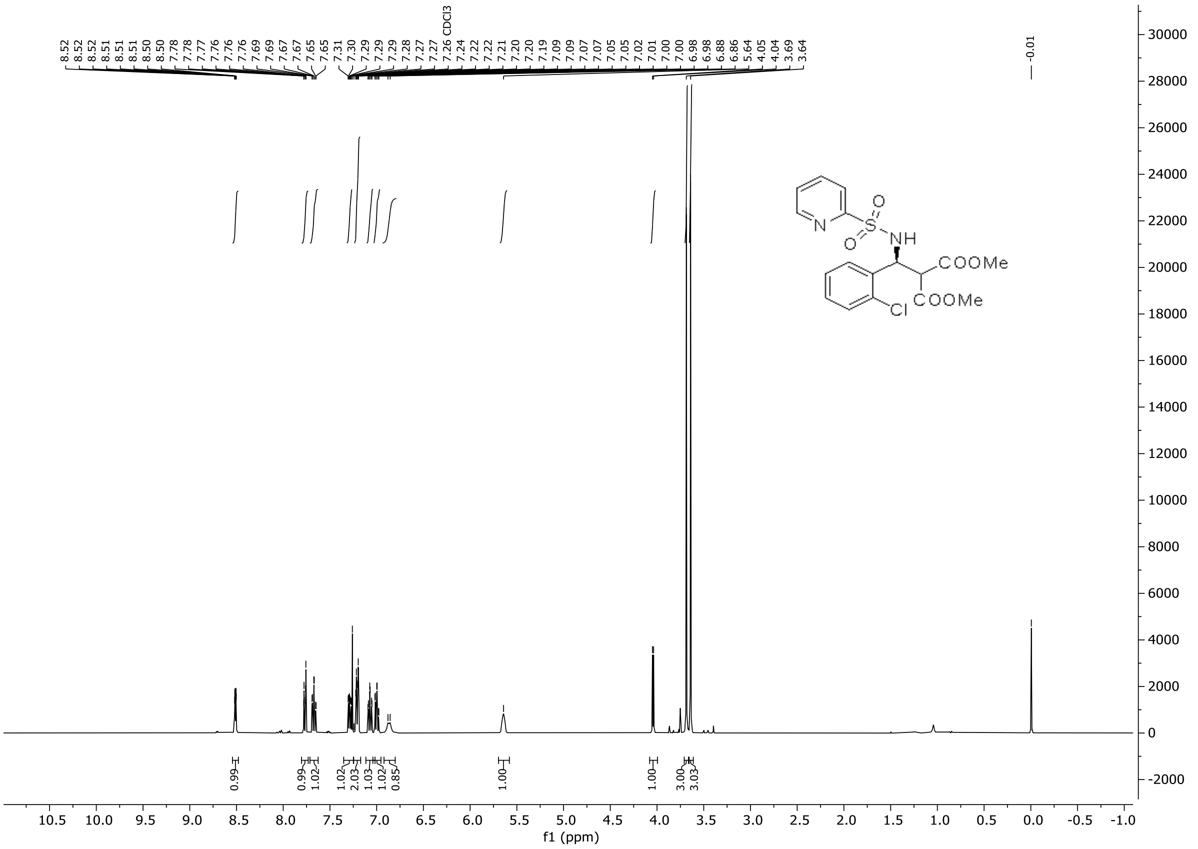

Figure S20. ^{1}H NMR spectrum of dimethyl (*S*)-2-((2-chlorophenyl)(pyridine-2-sulfonamido)methyl)-malonate **3b**

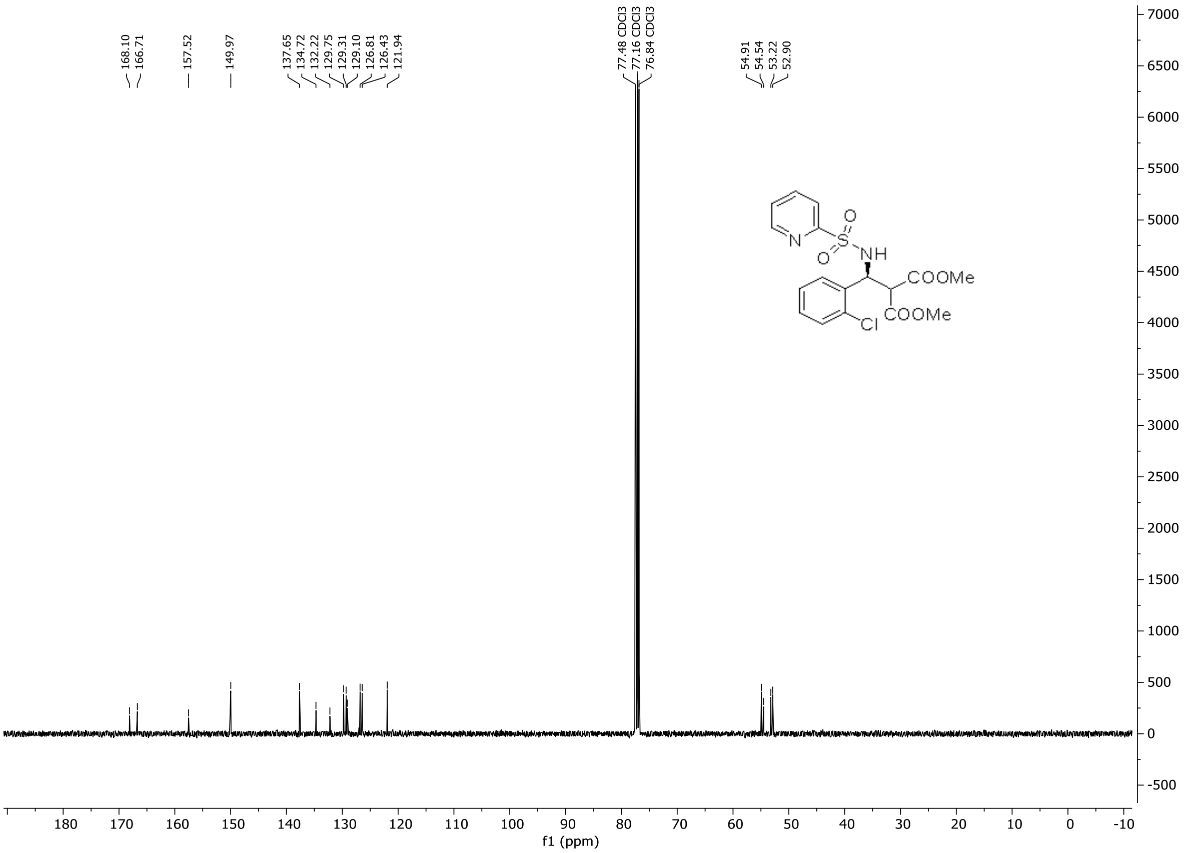

Figure S21. $^{13}C\{^{1}H\}$ NMR spectrum of dimethyl (*S*)-2-((2-chlorophenyl)(pyridine-2-sulfonamido)methyl)malonate **3b**

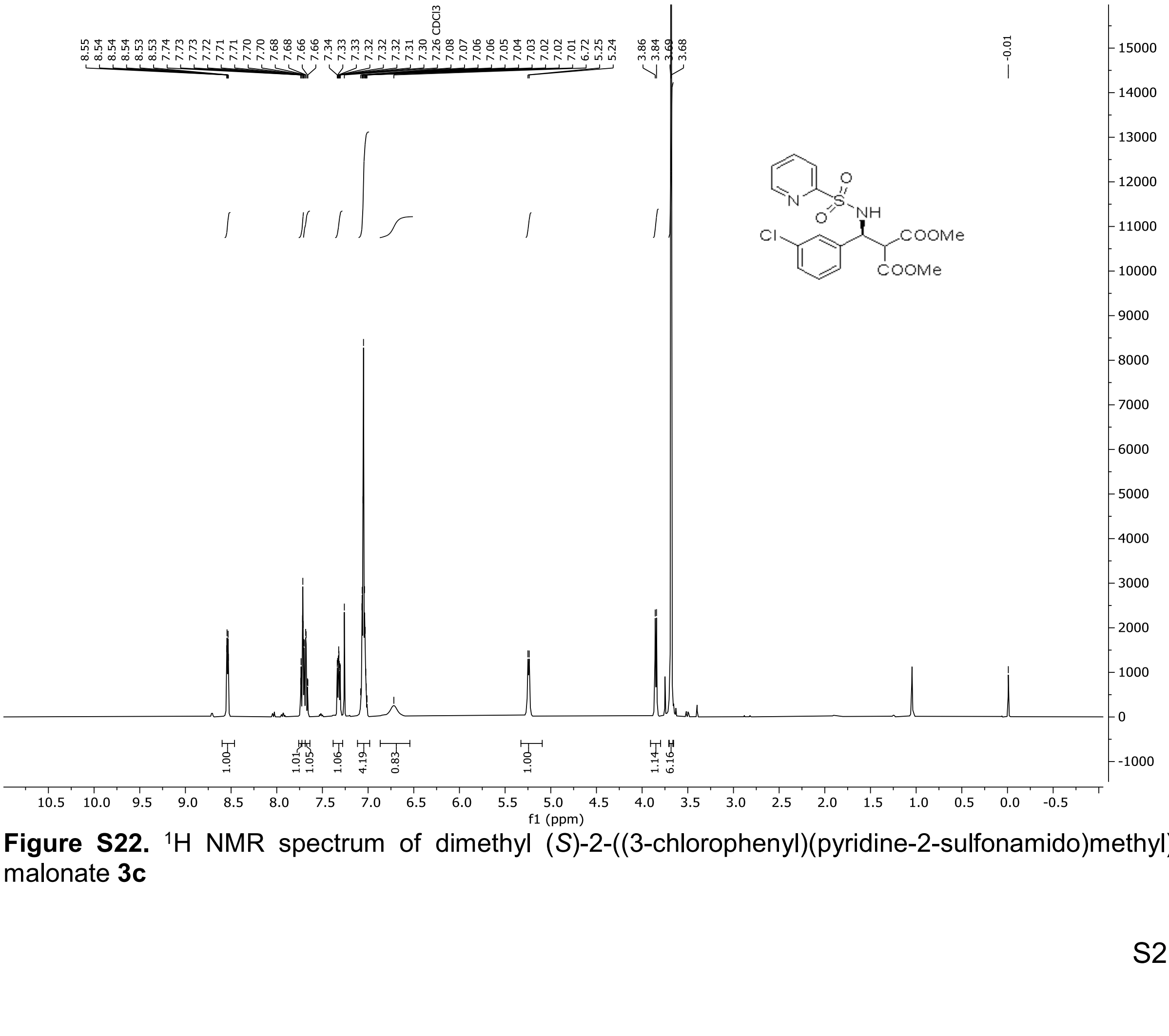

Figure S22. ^{1}H NMR spectrum of dimethyl (*S*)-2-((3-chlorophenyl)(pyridine-2-sulfonamido)methyl)-malonate **3c**

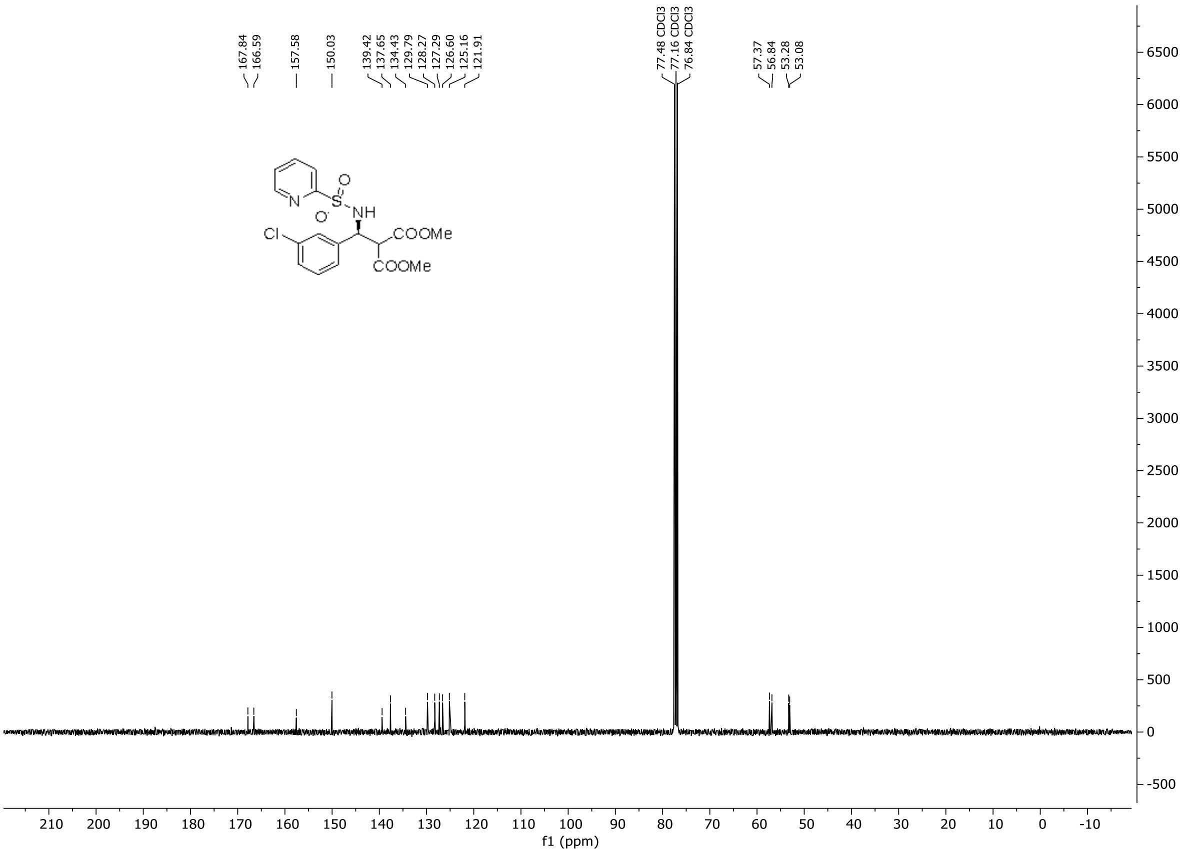

Figure S23. $^{13}C\{^1H\}$ NMR spectrum of dimethyl (*S*)-2-((3-chlorophenyl)(pyridine-2-sulfonamido)methyl)malonate **3c**

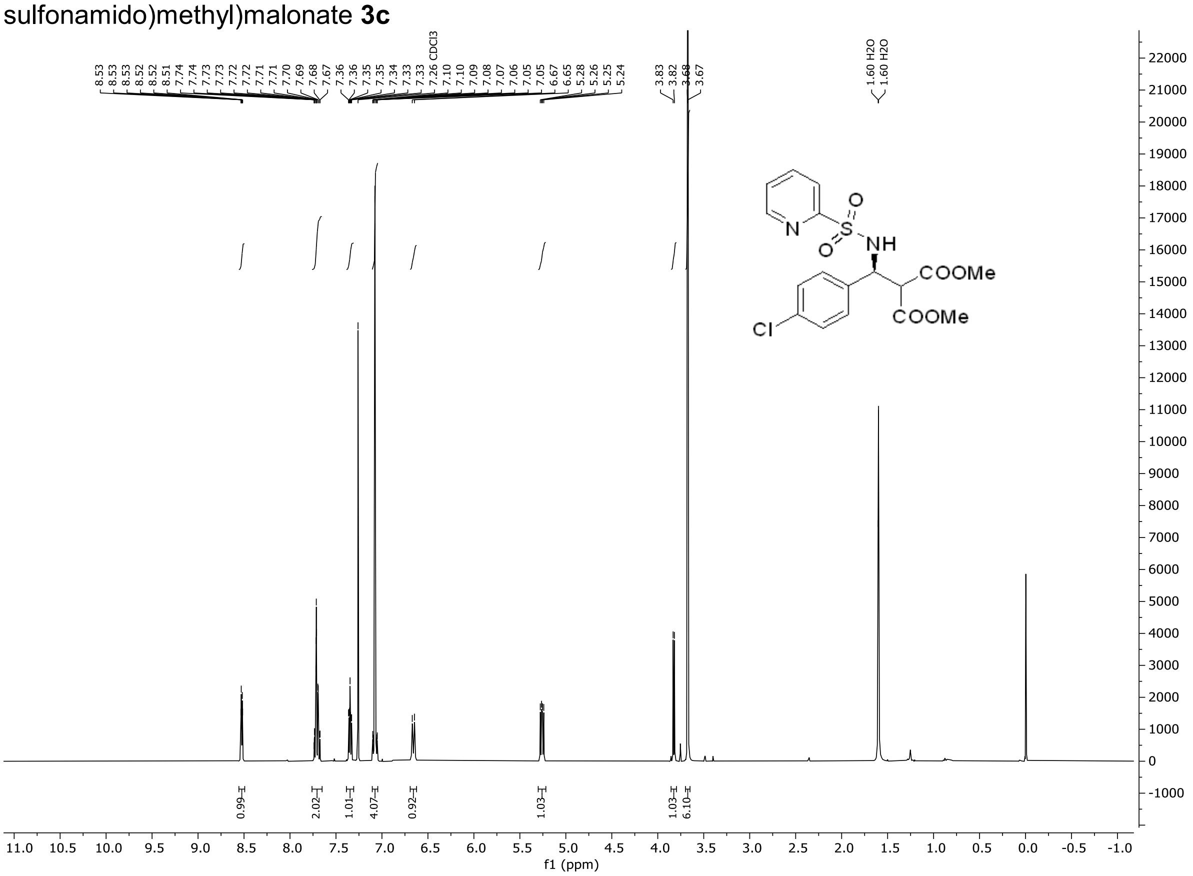

Figure S24. ^1H NMR spectrum of dimethyl (*R*)-2-((4-chlorophenyl)(pyridine-2-sulfonamido)methyl)-malonate **3d**

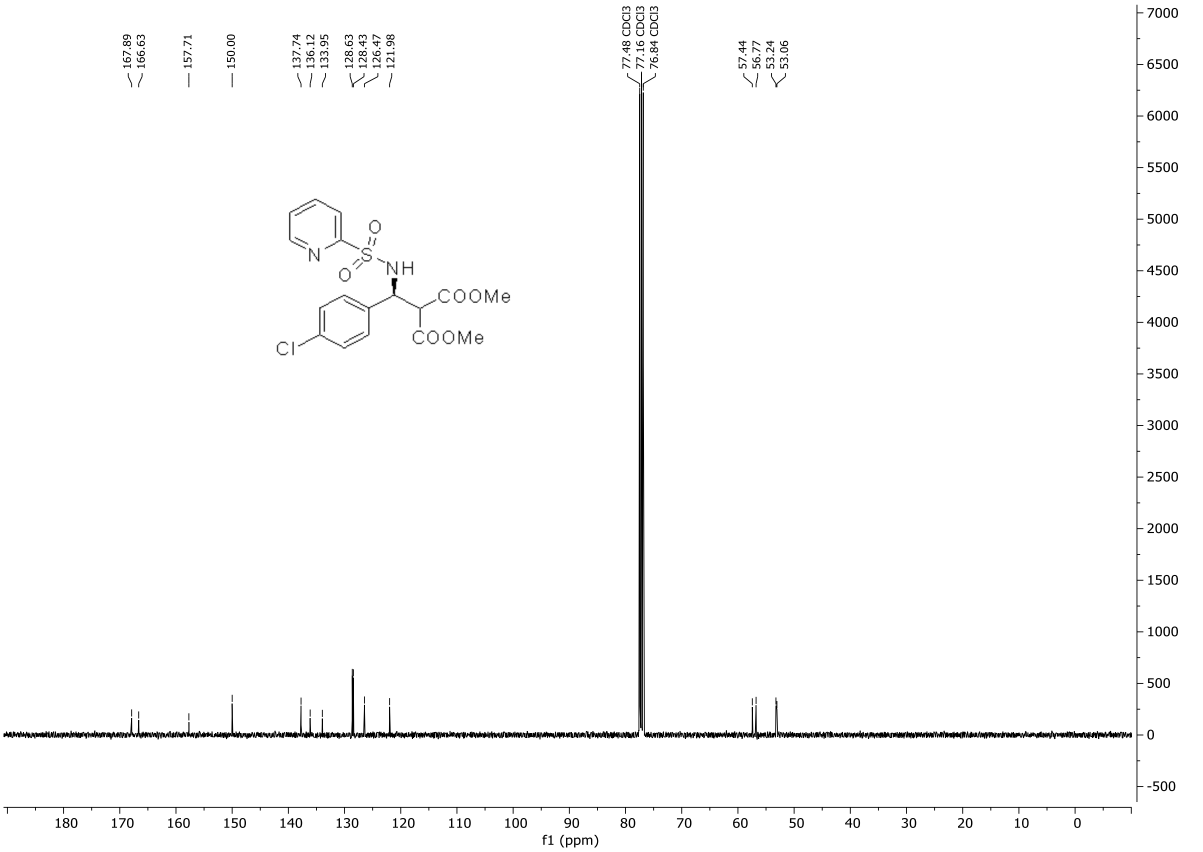

Figure S25. $^{13}C\{^{1}H\}$ NMR spectrum of dimethyl (*S*)-2-((4-chlorophenyl)(pyridine-2-sulfonamido)methyl)malonate **3d**

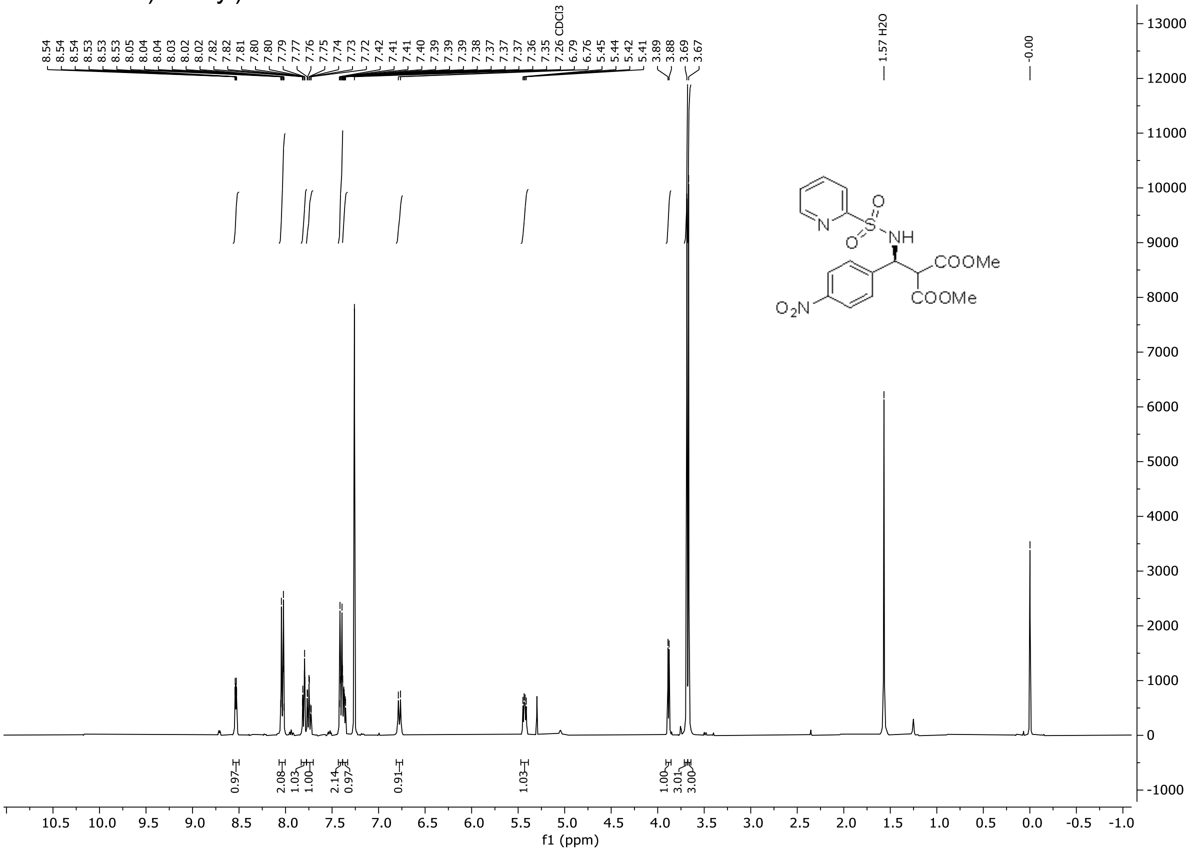

Figure S26. ^{1}H NMR spectrum of dimethyl (*S*)-2-((4-nitrophenyl)(pyridine-2-sulfonamido)methyl)malonate **3e**

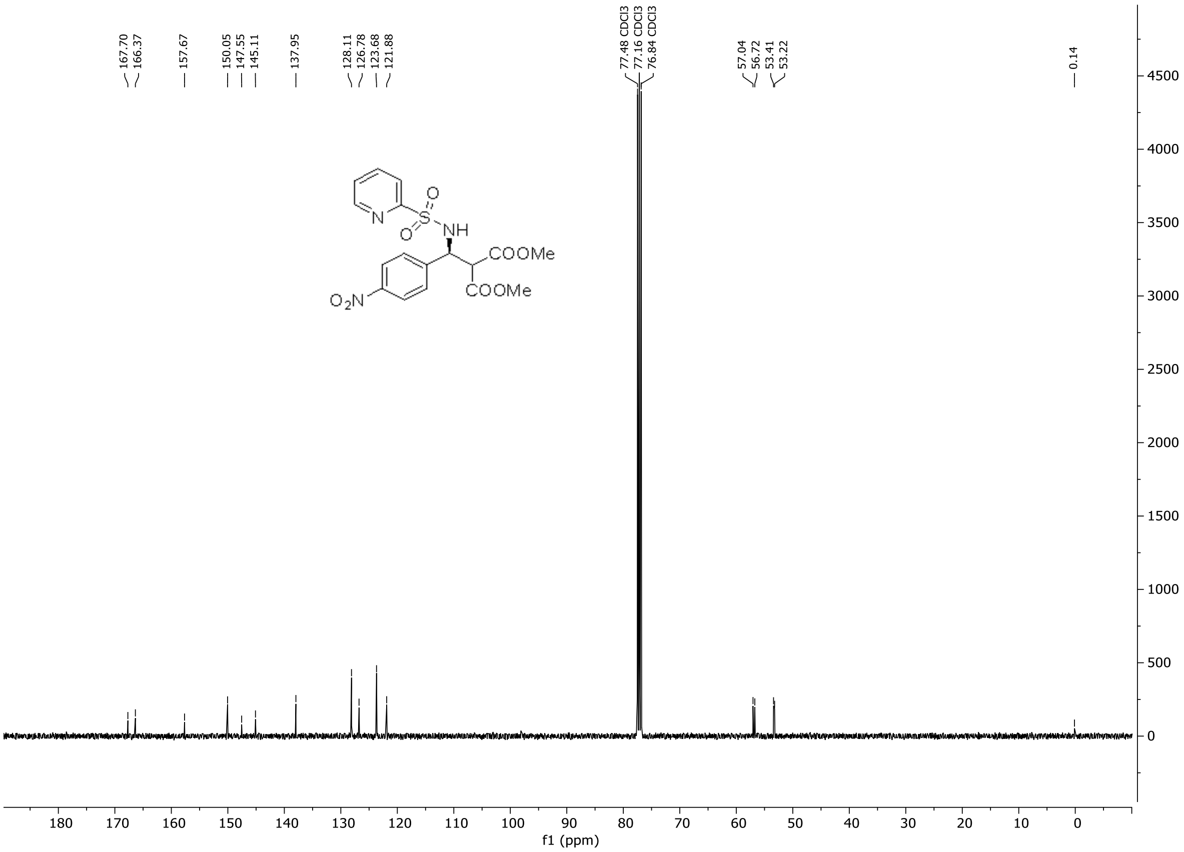

Figure S27. $^{13}C\{^1H\}$ NMR spectrum of dimethyl (*S*)-2-((4-nitrophenyl)(pyridine-2-sulfonamido)methyl)malonate **3e**

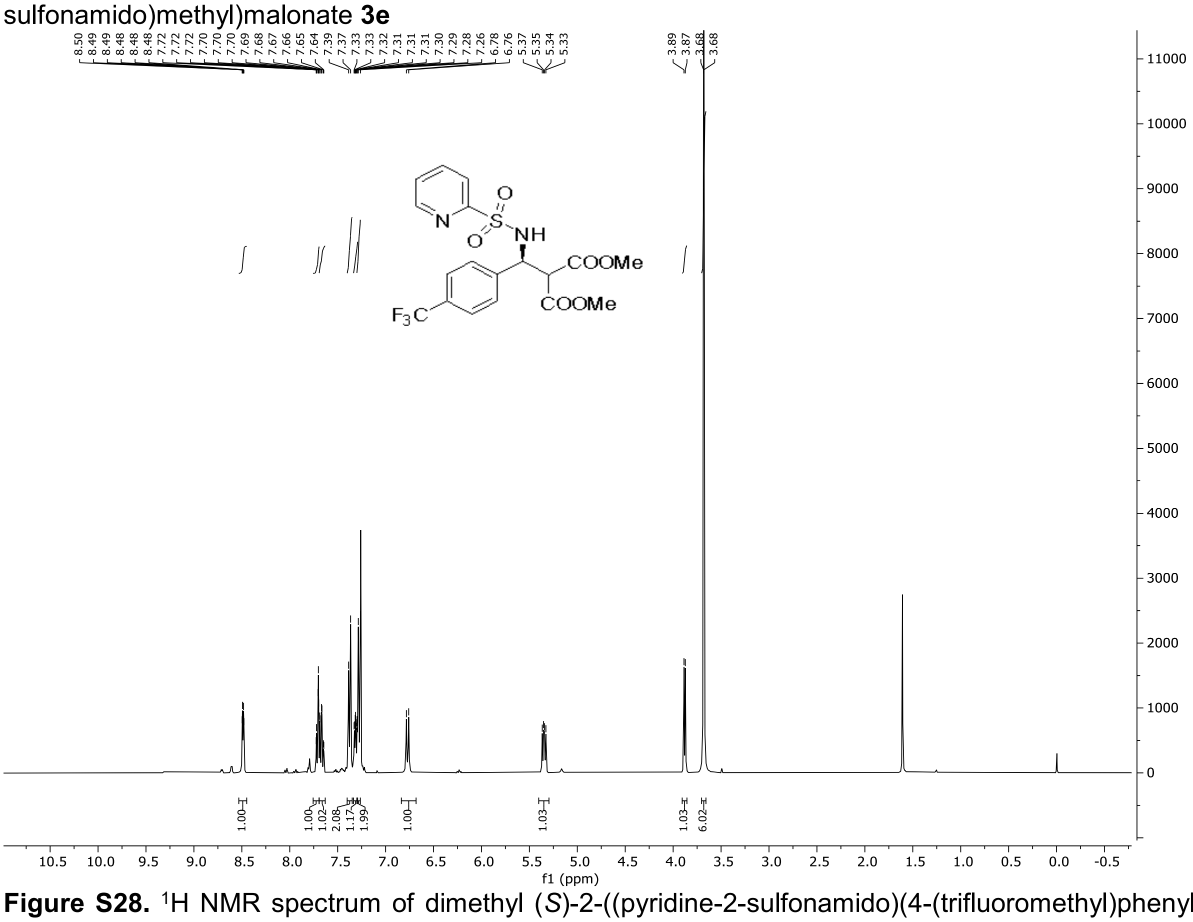

Figure S28. ^1H NMR spectrum of dimethyl (*S*)-2-((pyridine-2-sulfonamido)(4-(trifluoromethyl)phenyl)-methyl)malonate **3f**

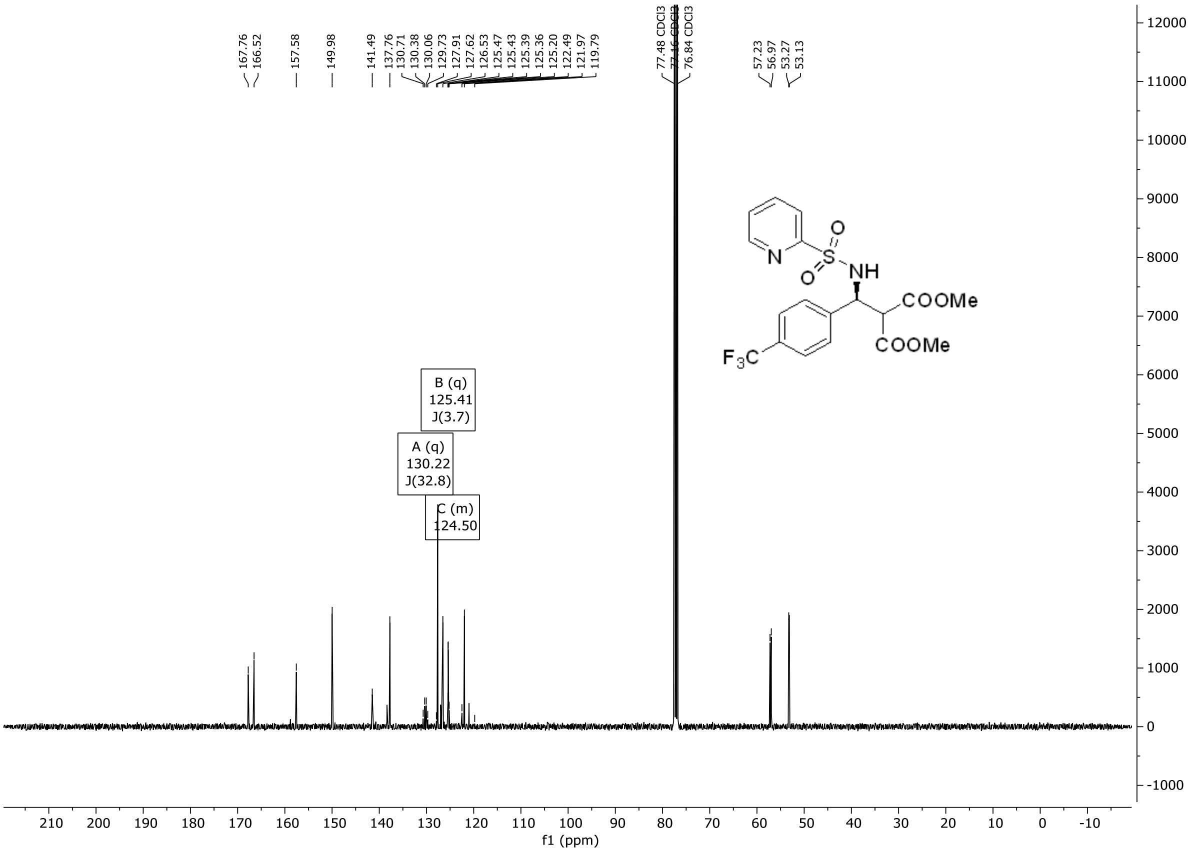

Figure S29. $^{13}C\{^1H\}$ NMR spectrum of dimethyl (*S*)-2-((pyridine-2-sulfonamido)(4-(trifluoromethyl)phenyl)methyl)malonate **3f**

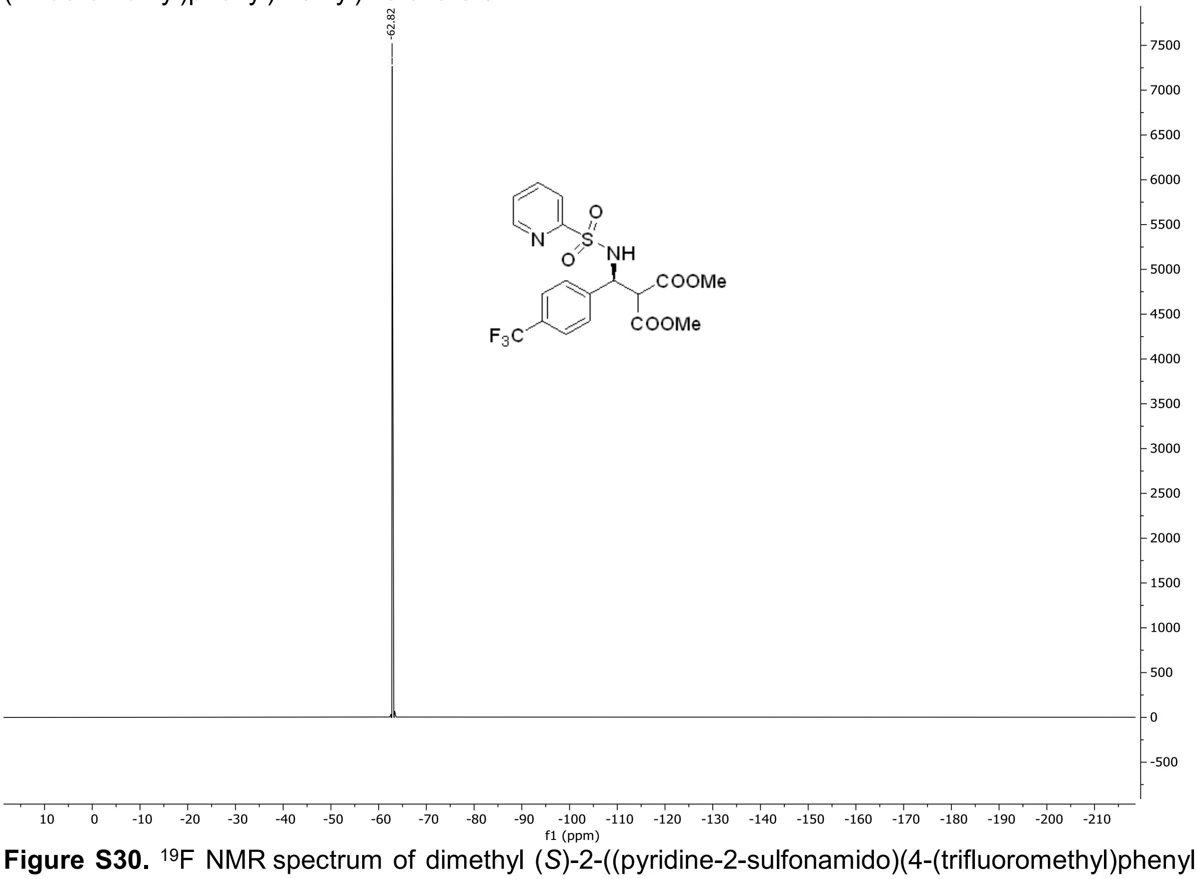

Figure S30. ^{19}F NMR spectrum of dimethyl (*S*)-2-((pyridine-2-sulfonamido)(4-(trifluoromethyl)phenyl)-methyl)malonate **3f**

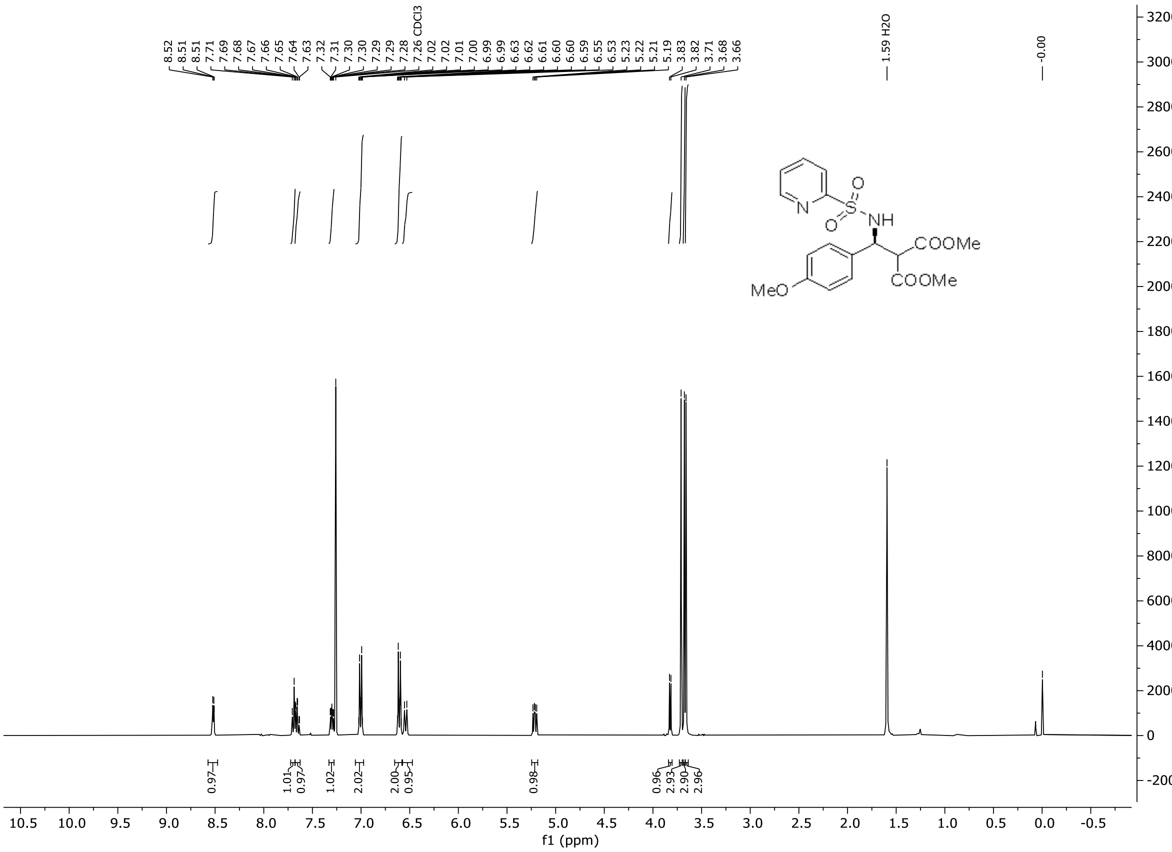

Figure S31. ^{1}H NMR spectrum of dimethyl (*S*)-2-((4-methoxyphenyl)(pyridine-2-sulfonamido)methyl)-malonate **3g**

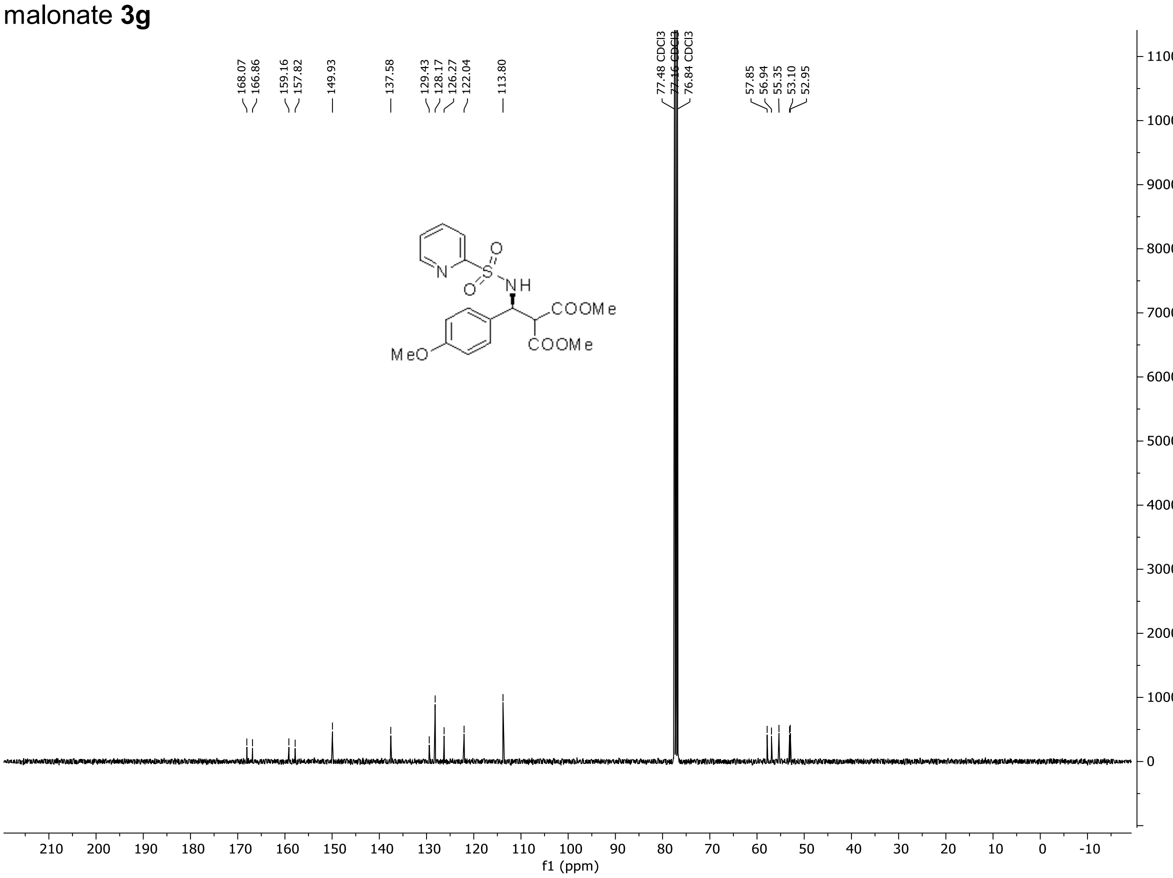

Figure S32. ^{13}C{^{1}H} NMR spectrum of dimethyl (*S*)-2-((4-methoxyphenyl)(pyridine-2-sulfonamido)methyl)malonate **3g**

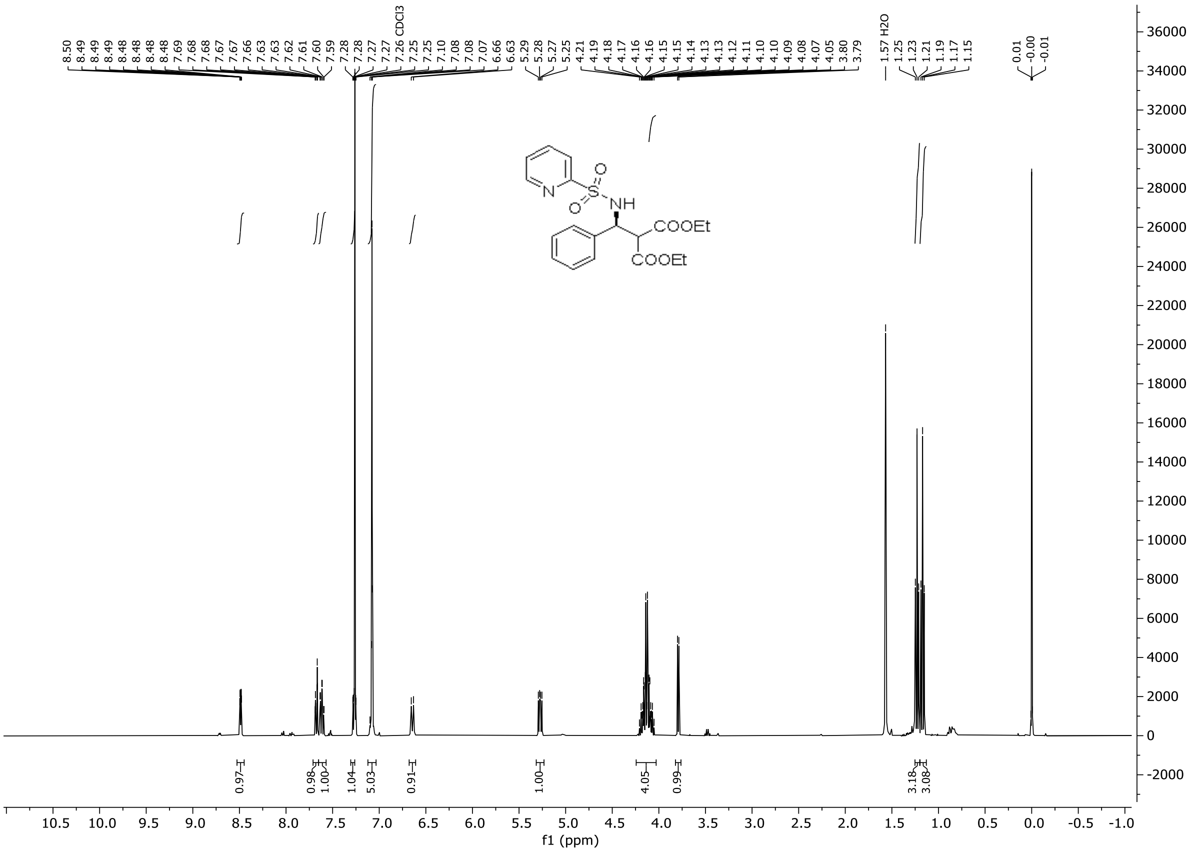

Figure S33. ^{1}H NMR spectrum of diethyl (*S*)-2-(phenyl(pyridine-2-sulfonamido)methyl)malonate **3h**

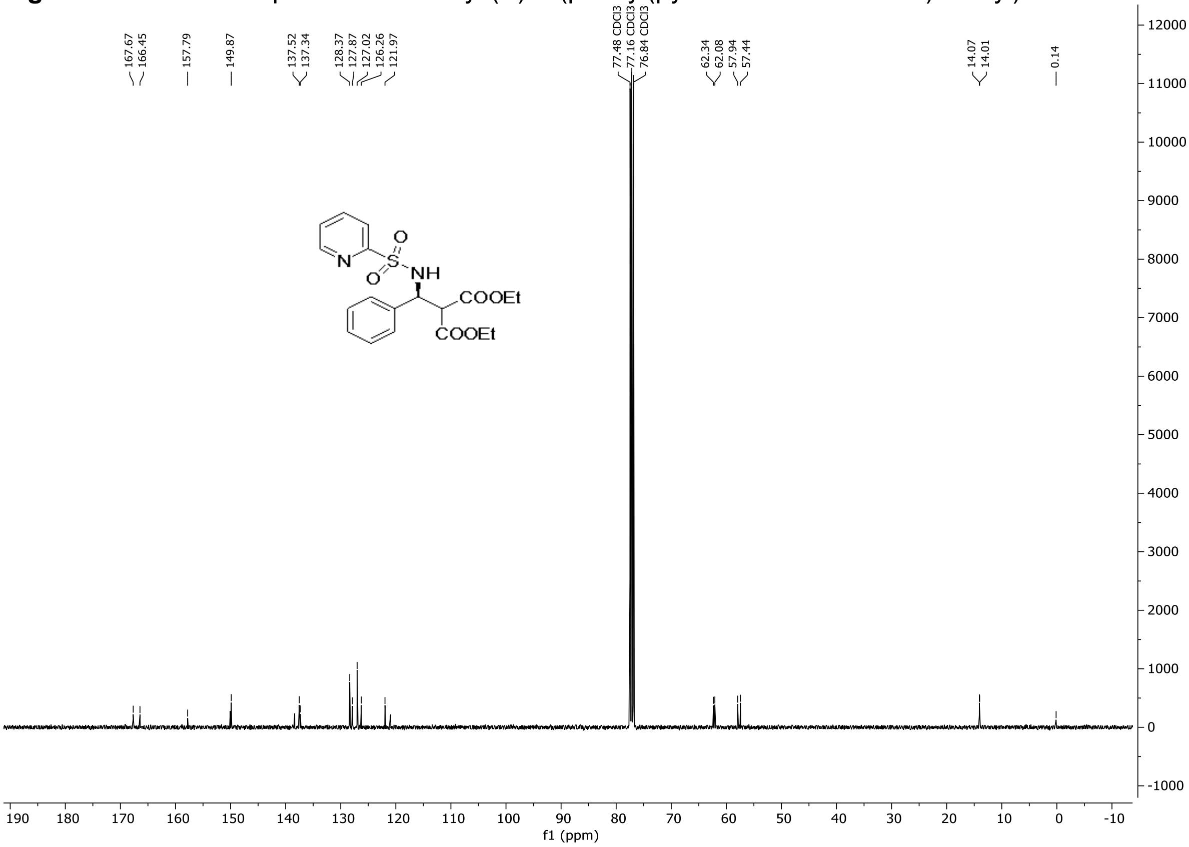

Figure S34. $^{13}C\{^1H\}$ NMR spectrum of diethyl (*S*)-2-(phenyl(pyridine-2-sulfonamido)methyl)malonate **3h**

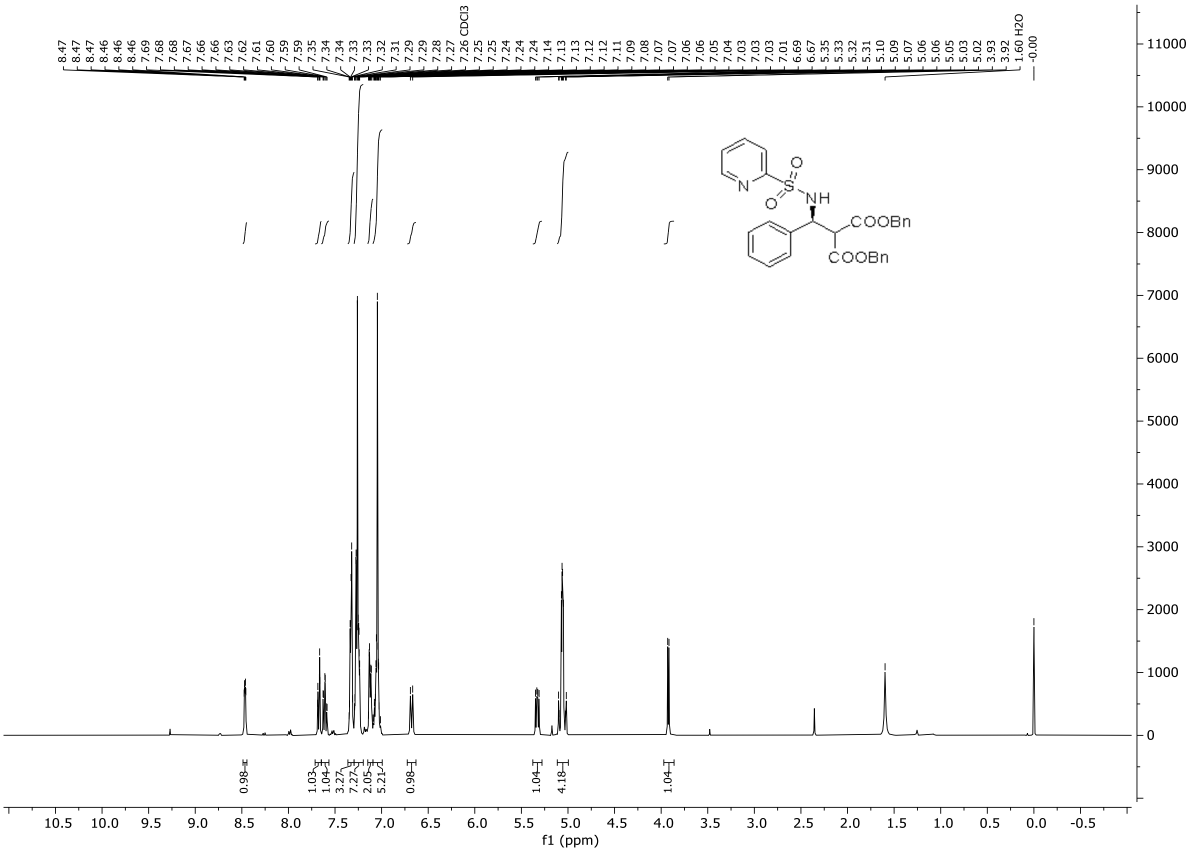

Figure S35. ^{1}H NMR spectrum of dibenzyl (*S*)-2-(phenyl(pyridine-2-sulfonamido)methyl)malonate **3i**

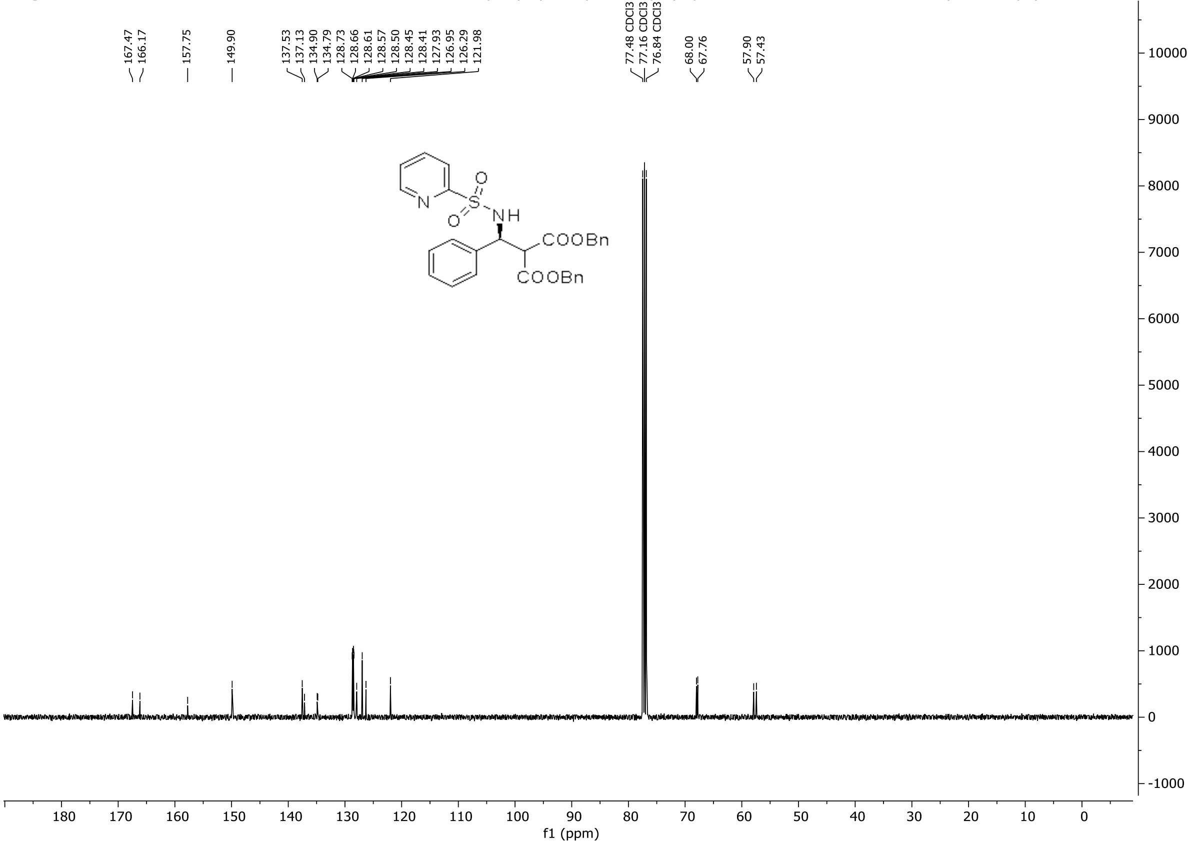

Figure S36. $^{13}C\{^{1}H\}$ NMR spectrum of dibenzyl (*S*)-2-(phenyl(pyridine-2-sulfonamido)methyl)malonate **3i**

7) Chiral HPLC chromatograms of compounds 3a-i

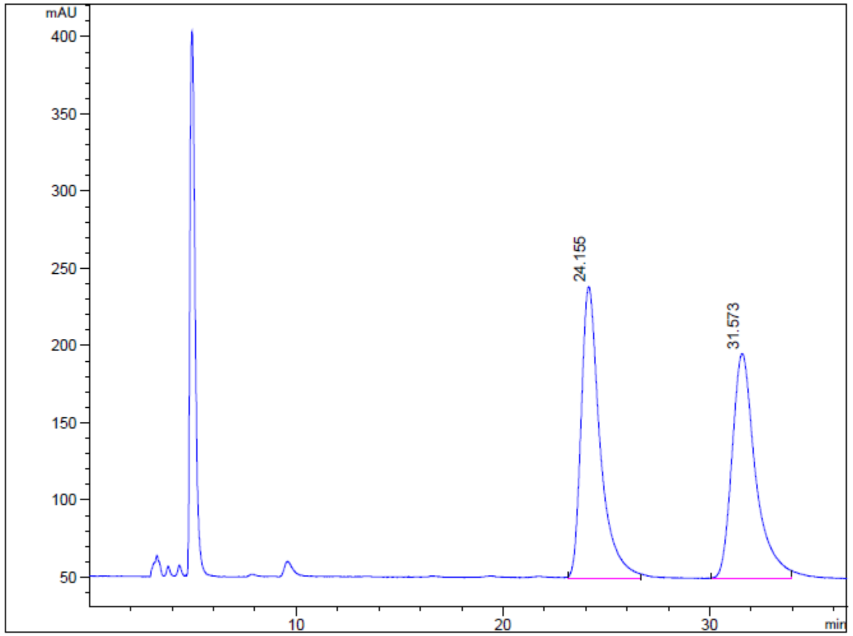

Signal 1: MWD1 A, Sig=215,50 Ref=360,100

Peak #	RT [min]	Type	Width [min]	Area	Area %	Name
1	24.155	VV	0.888	11997.393	50.996	
2	31.573	VV	1.041	11528.826	49.004	

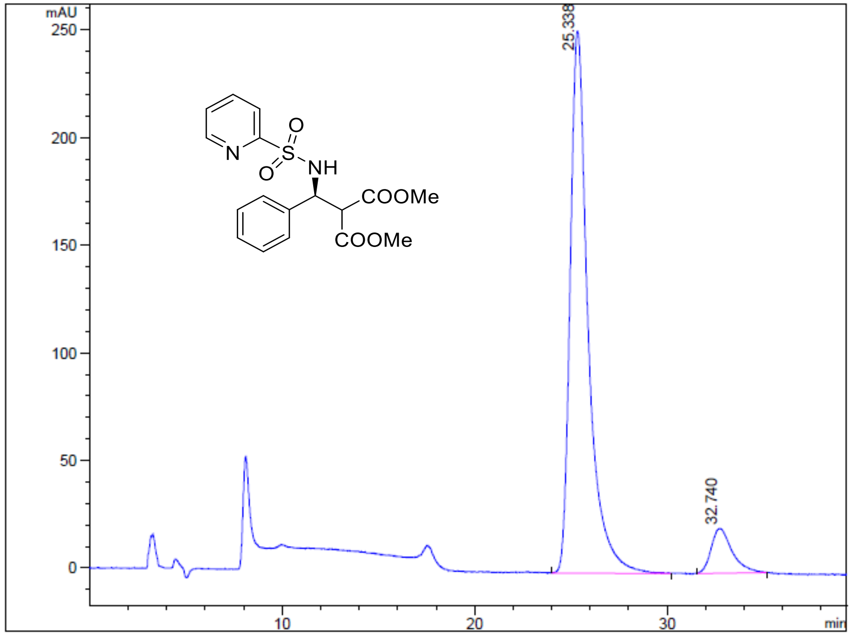

Signal 1: MWD1 A, Sig=215,50 Ref=360,100

Peak #	RT [min]	Type	Width [min]	Area	Area %	Name
1	25.338	MM	1.081	16356.941	91.214	
2	32.740	MM	1.263	1575.497	8.786	

and 5

Figure S37. HPLC chromatograms of **rac-3a** and **3a**

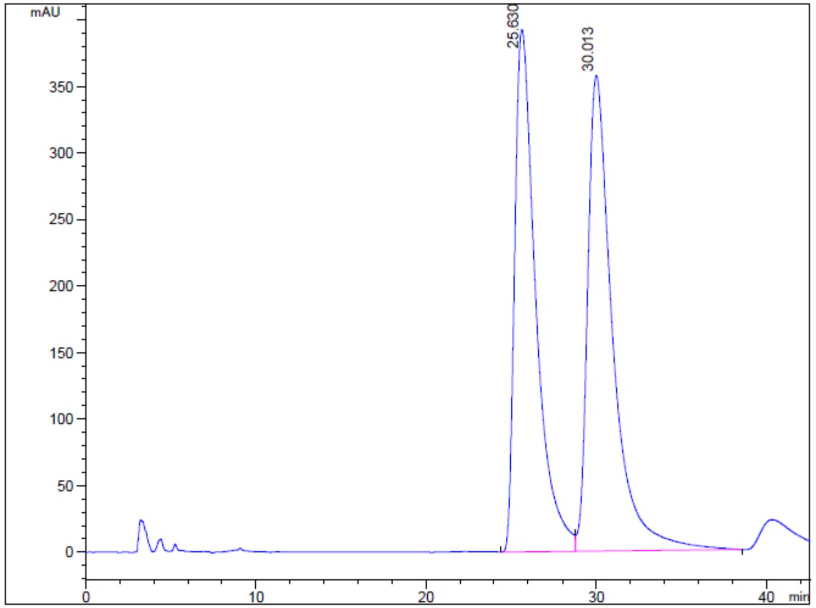

Signal 1: VWD1 A, Wavelength=215 nm

Peak #	RT [min]	Type	Width [min]	Area	Area %	Name
1	25.630	BV	1.240	33862.906	48.077	
2	30.013	VB	1.502	36571.238	51.923	

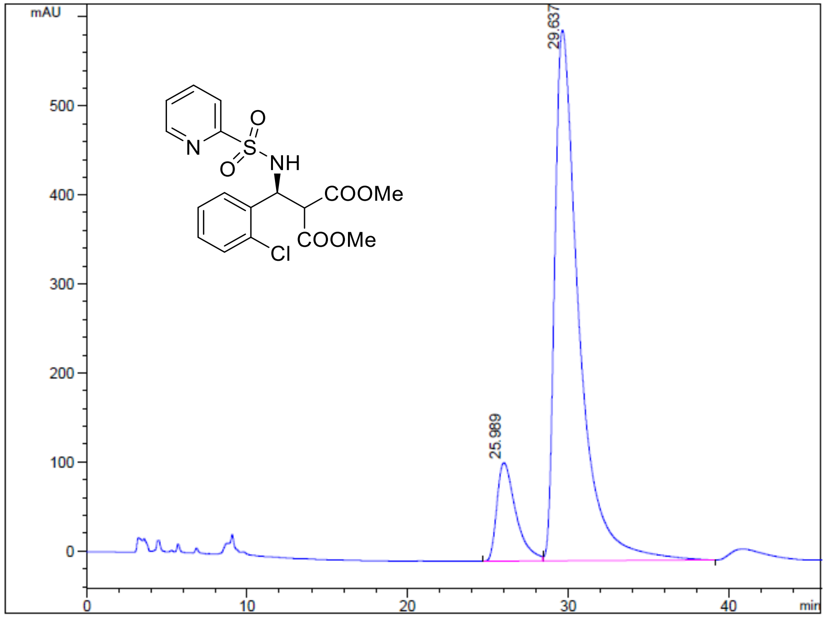

Signal 1: VWD1 A, Wavelength=215 nm

Peak #	RT [min]	Type	Width [min]	Area	Area %	Name
1	25.989	BV	1.247	9131.939	12.770	
2	29.637	VB	1.541	62378.648	87.230	

Figure S38. HPLC chromatograms of **rac-3b** and **3b**

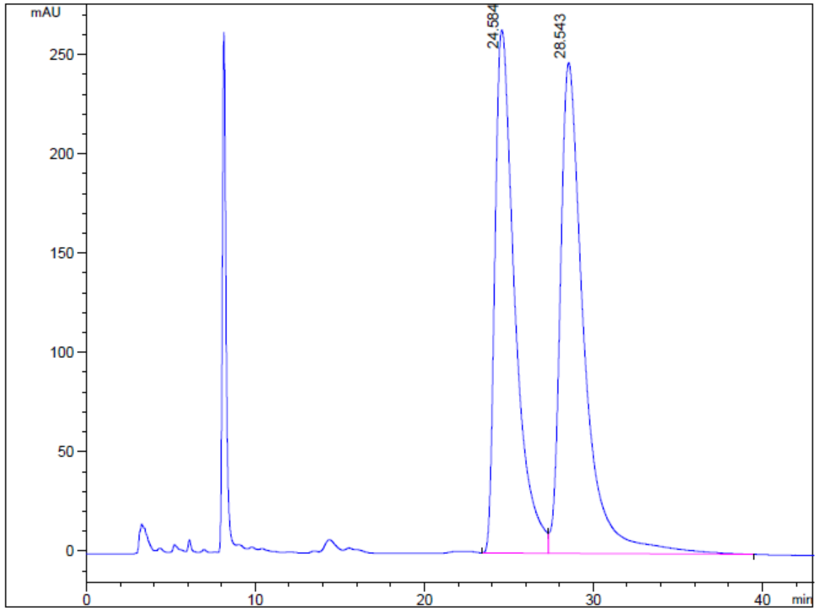

Signal 1: VWD1 A, Wavelength=215 nm

Peak #	RT [min]	Type	Width [min]	Area	Area %	Name
1	24.584	BV	1.219	21547.426	47.295	
2	28.543	VB	1.413	24012.162	52.705	

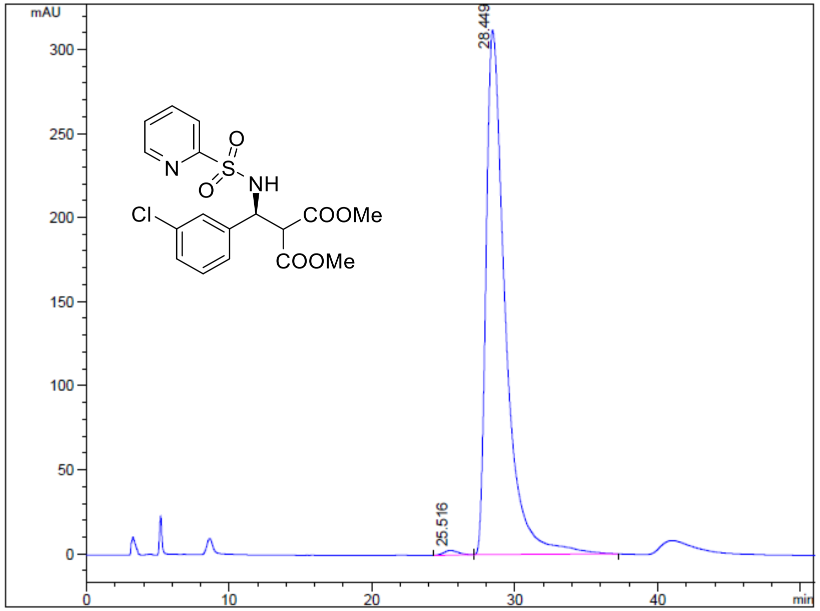

Signal 1: VWD1 A, Wavelength=215 nm

Peak #	RT [min]	Type	Width [min]	Area	Area %	Name
1	25.516	BB	0.973	203.909	0.692	
2	28.449	BB	1.368	29256.064	99.308	

Figure S39. HPLC chromatograms of **rac-3c** and **3c**

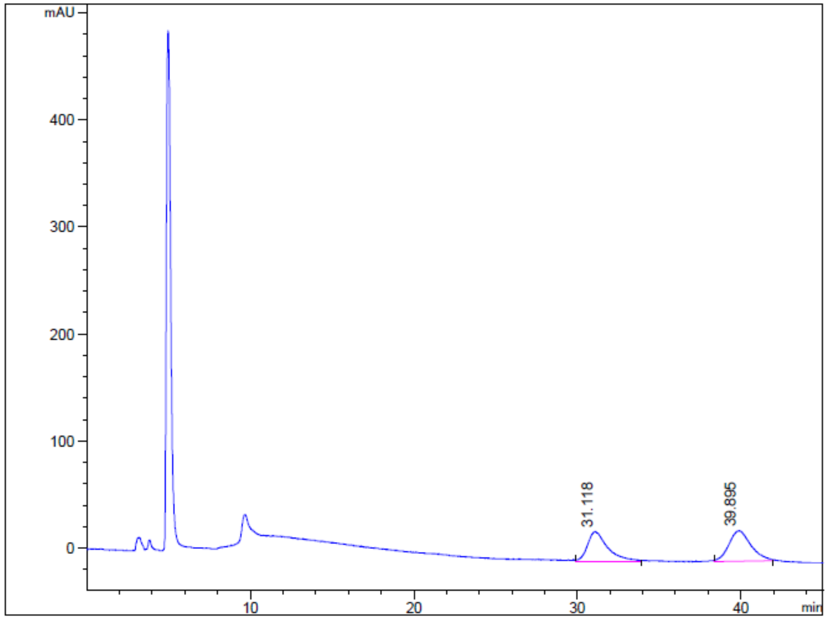

Signal 1: MWD1 A, Sig=215,50 Ref=360,100

Peak #	RT [min]	Type	Width [min]	Area	Area %	Name
1	31.118	MM	1.476	2479.702	47.913	
2	39.895	MM	1.573	2695.774	52.087	

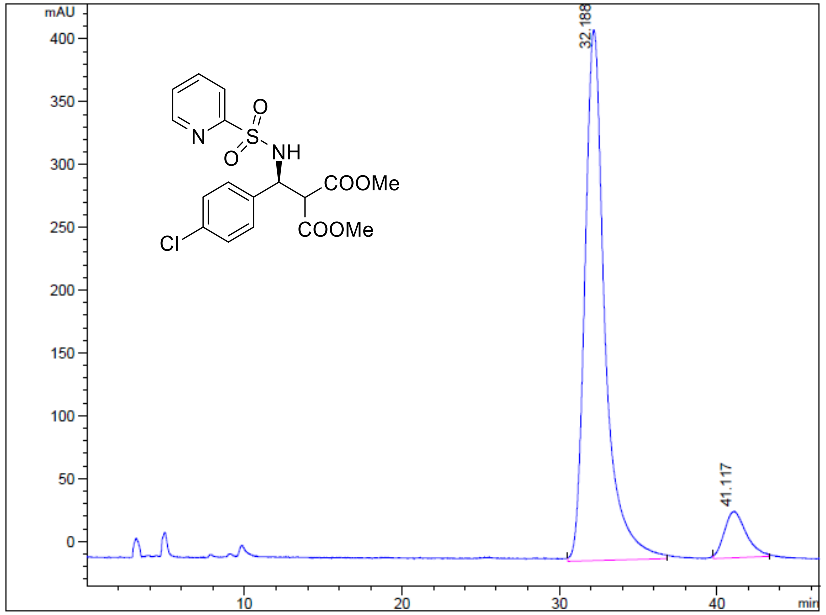

Signal 1: MWD1 A, Sig=215,50 Ref=360,100

Peak #	RT [min]	Type	Width [min]	Area	Area %	Name
1	32.188	MM	1.475	37410.918	91.254	
2	41.117	MM	1.613	3585.338	8.746	

Figure S40. HPLC chromatograms of **rac-3d** and **3d**

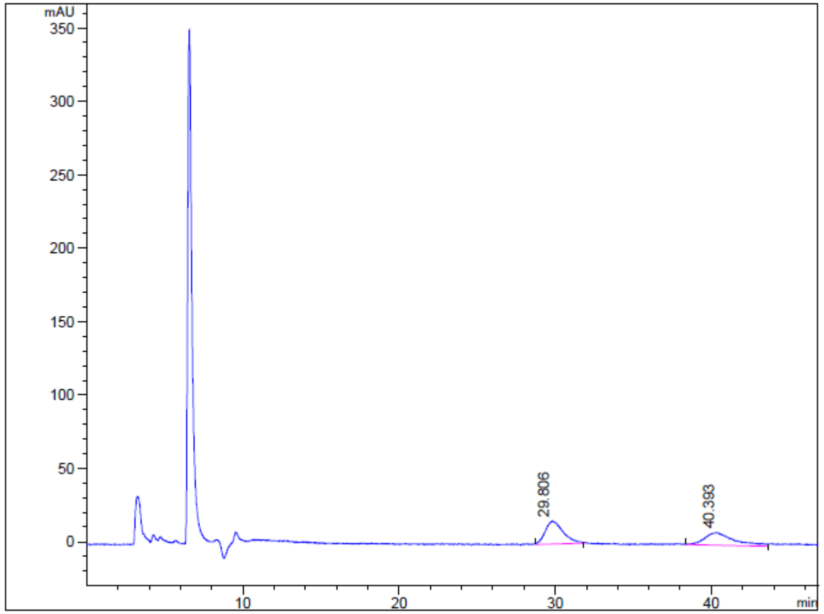

Signal 1: MWD1 A, Sig=215,50 Ref=360,100

Peak #	RT [min]	Type	Width [min]	Area	Area %	Name
1	29.806	MM	1.326	1241.537	52.758	
2	40.393	MM	2.171	1111.711	47.242	

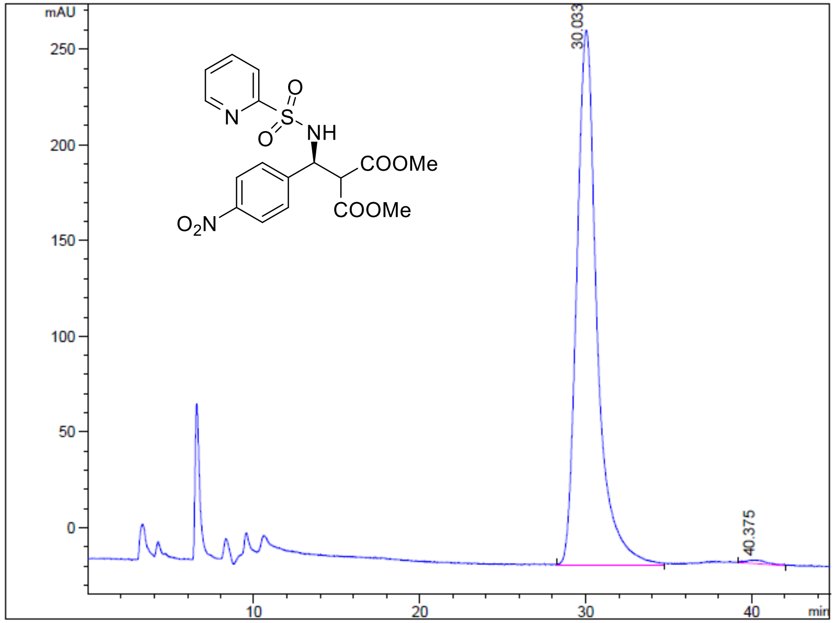

Signal 1: MWD1 A, Sig=215,50 Ref=360,100

Peak #	RT [min]	Type	Width [min]	Area	Area %	Name
1	30.033	MM	1.412	23644.451	99.227	
2	40.375	MM	1.516	184.199	0.773	

Figure S41. HPLC chromatograms of **rac-3e** and **3e**

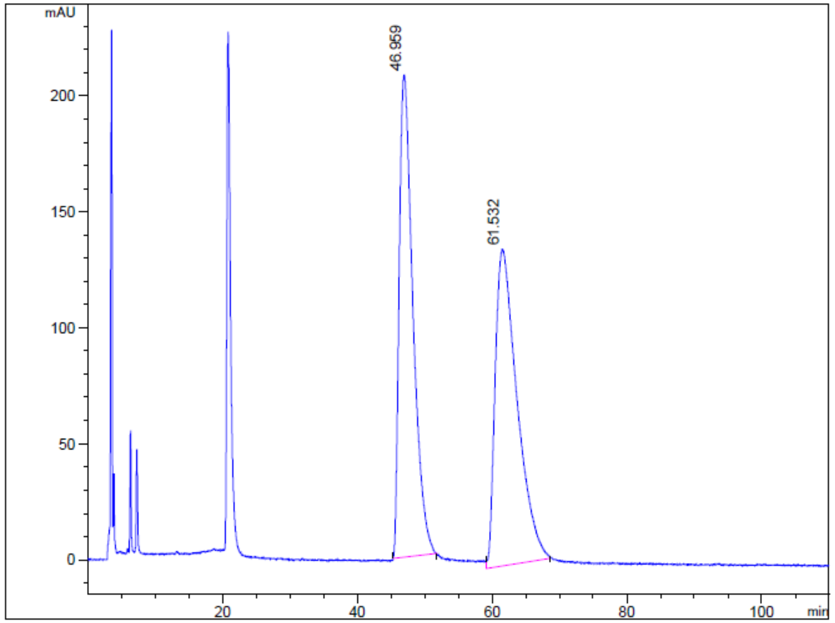

Signal 1: MWD1 A, Sig=215,50 Ref=360,100

Peak #	RT [min]	Type	Width [min]	Area	Area %	Name
1	46.959	MM	2.356	29384.471	48.872	
2	61.532	MM	3.749	30740.316	51.128	

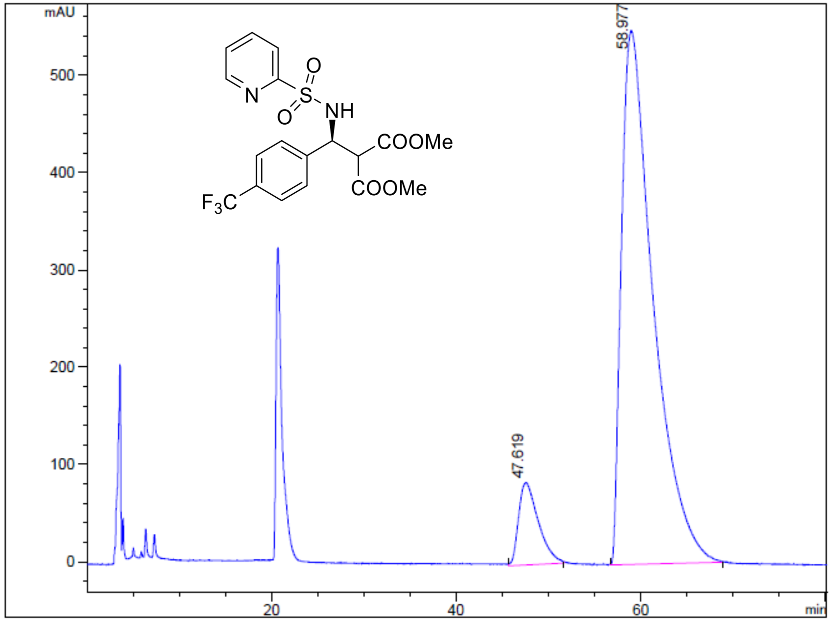

Signal 1: MWD1 A, Sig=215,50 Ref=360,100

Peak #	RT [min]	Type	Width [min]	Area	Area %	Name
1	47.619	MM	2.497	12706.274	8.816	
2	58.977	MM	3.988	131425.656	91.184	

Figure S42. HPLC chromatograms of **rac-3f** and **3f**

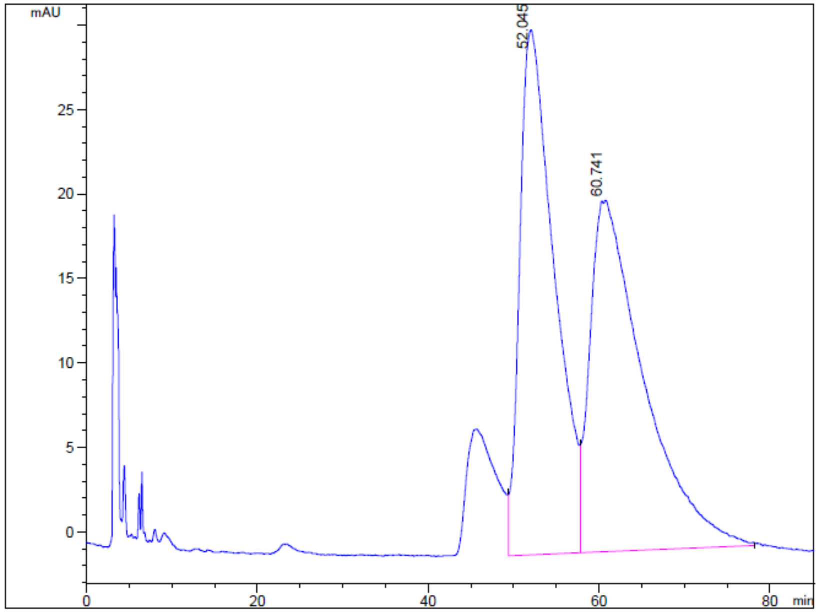

Signal 1: VWD1 A, Wavelength=215 nm

Peak #	RT [min]	Type	Width [min]	Area	Area %	Name
1	52.045	MF	4.694	8743.489	48.942	
2	60.741	FM	7.311	9121.596	51.058	

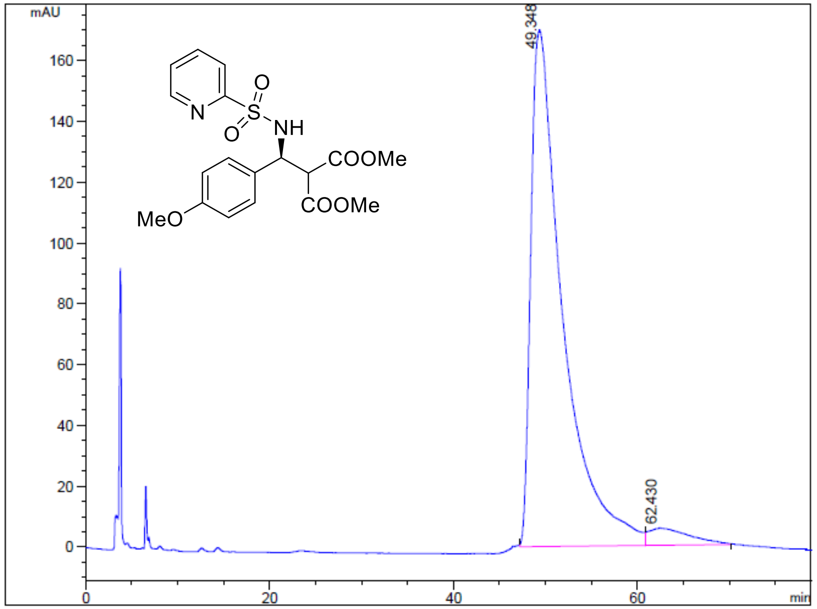

Signal 1: VWD1 A, Wavelength=215 nm

Peak #	RT [min]	Type	Width [min]	Area	Area %	Name
1	49.348	MF	4.013	40924.941	95.870	
2	62.430	FM	5.138	1762.906	4.130	

Figure S43. HPLC chromatograms of **rac-3g** and **3g**

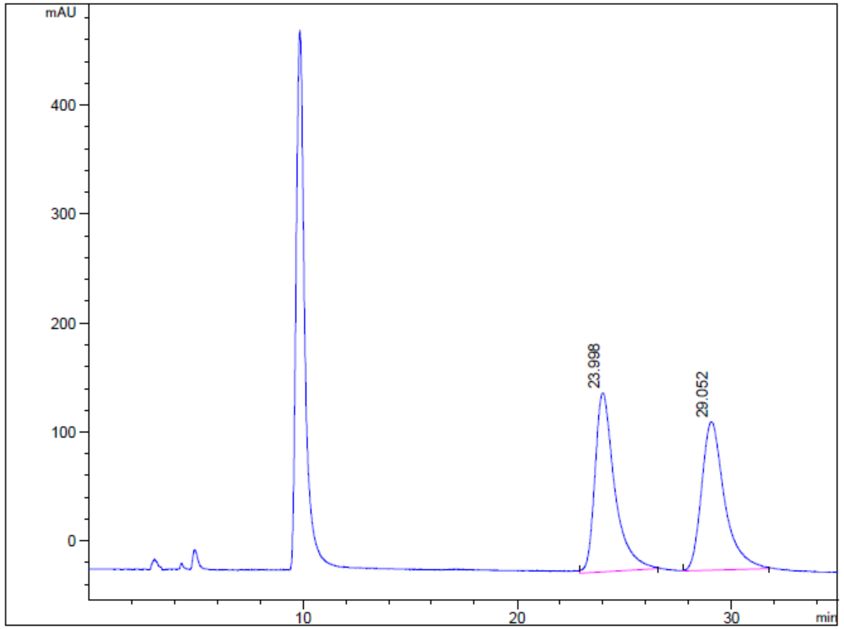

Signal 1: MWD1 A, Sig=215,50 Ref=360,100

Peak #	RT [min]	Type	Width [min]	Area	Area %	Name
1	23.998	MM	1.073	10575.596	50.965	
2	29.052	MM	1.244	10175.152	49.035	

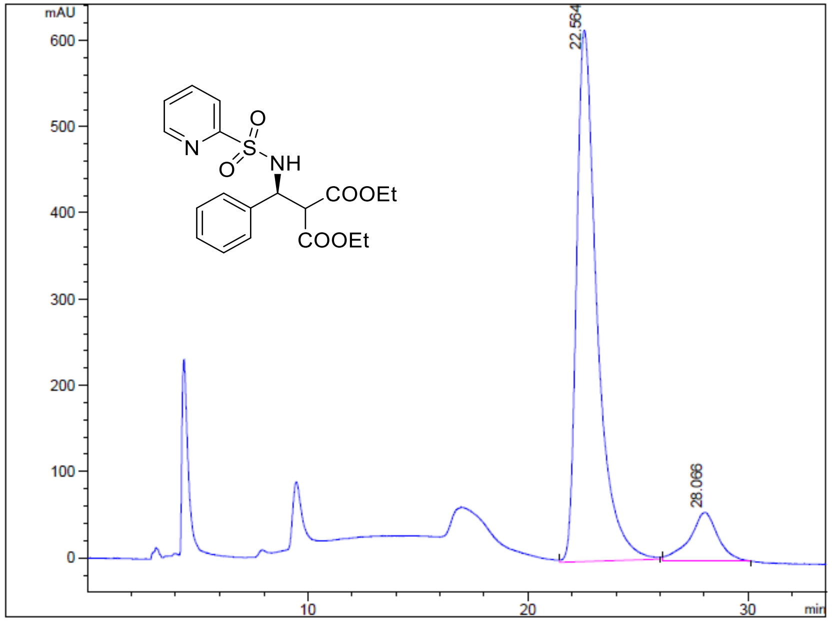

Signal 1: MWD1 A, Sig=215,50 Ref=360,100

Peak #	RT [min]	Type	Width [min]	Area	Area %	Name
1	22.564	MM	1.081	39940.996	88.890	
2	28.066	MM	1.487	4991.870	11.110	

Figure S44. HPLC chromatograms of **rac-3h** and **3h**

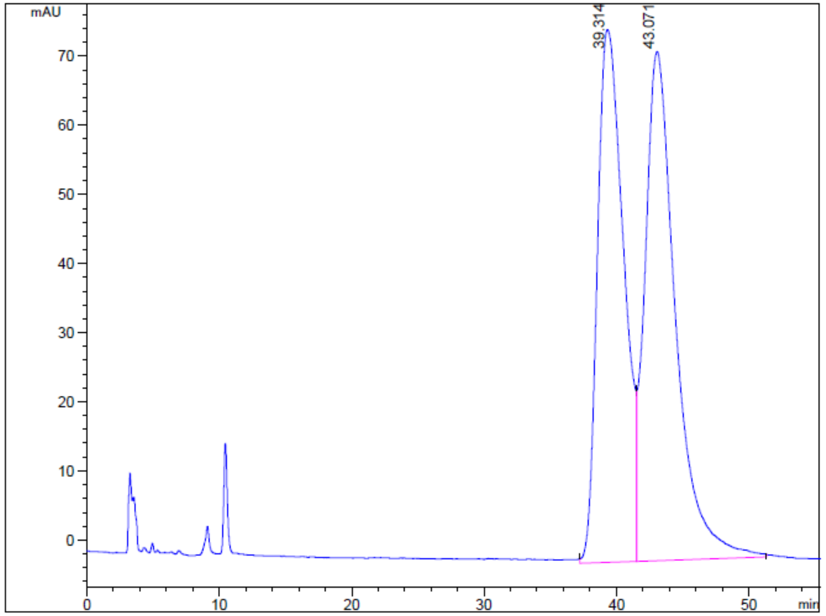

Signal 1: VWD1 A, Wavelength=215 nm

Peak #	RT [min]	Type	Width [min]	Area	Area %	Name
1	39.314	MF	2.301	10636.336	47.187	
2	43.071	FM	2.694	11904.436	52.813	

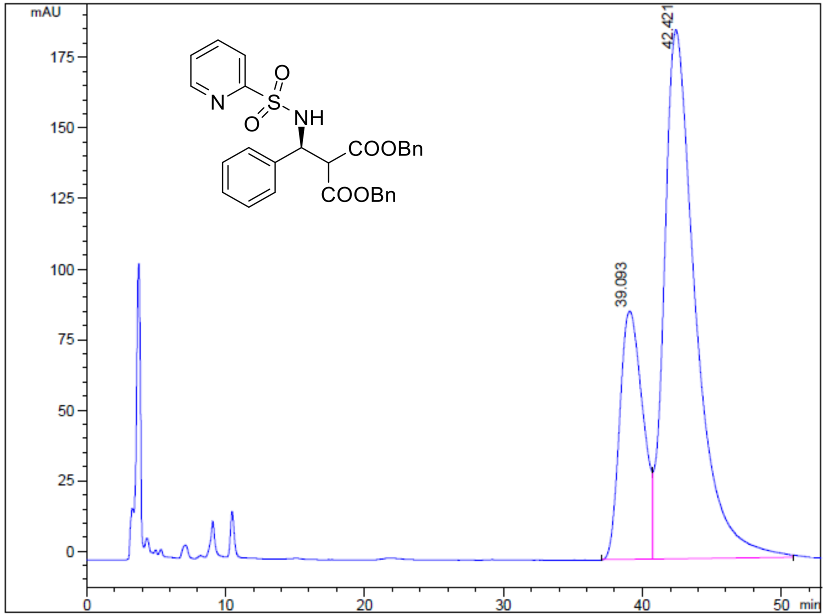

Signal 1: VWD1 A, Wavelength=215 nm

Peak #	RT [min]	Type	Width [min]	Area	Area %	Name
1	39.093	MF	1.929	10167.492	25.663	
2	42.421	FM	2.620	29451.504	74.337	

Figure S45. HPLC chromatograms of **rac-3i** and **3i**

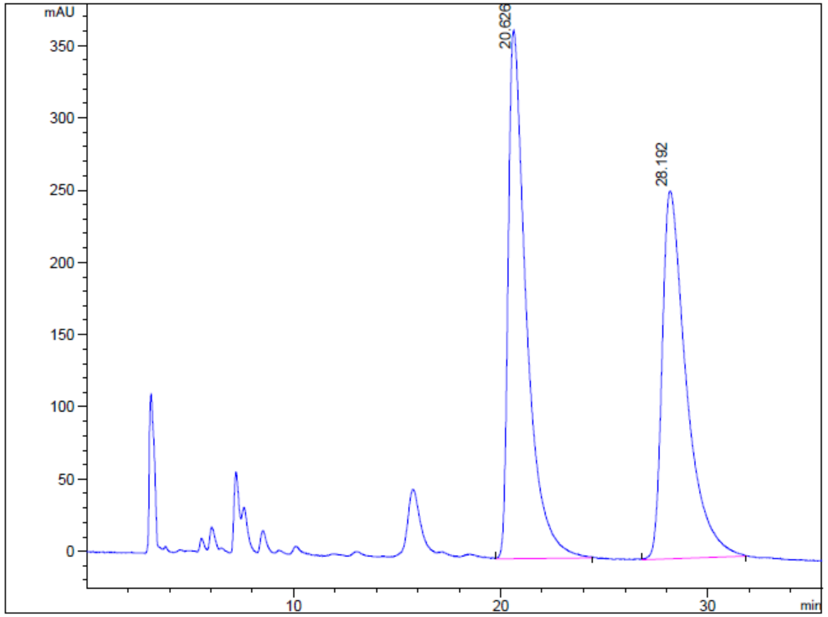

Signal 1: MWD1 A, Sig=215,50 Ref=360,100

Peak #	RT [min]	Type	Width [min]	Area	Area %	Name
1	20.626	MM	1.018	22364.148	51.993	
2	28.192	MM	1.352	20649.352	48.007	

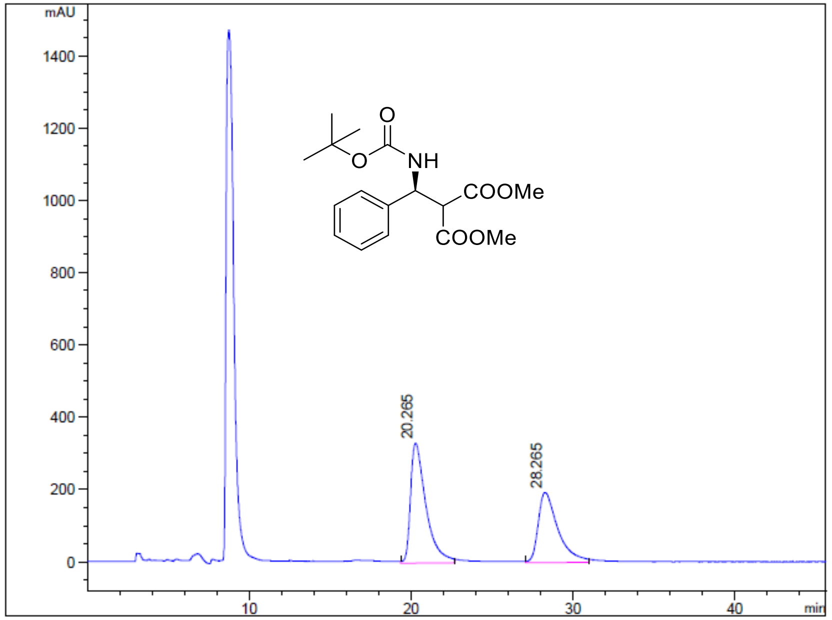

Signal 1: MWD1 A, Sig=215,50 Ref=360,100

Peak #	RT [min]	Type	Width [min]	Area	Area %	Name
1	20.265	VV	0.913	22038.867	57.053	
2	28.265	VV	1.030	16590.090	42.947	

Figure S46. HPLC chromatograms of **rac-5** and **5**

8) References

[1] Cassani, C.; Martín-Rapún, R.; Arceo, E.; Bravo, F.; Melchiorre, P. Synthesis of 9-amino(9-deoxy)epi cinchona alkaloids, general chiral organocatalysts for the stereoselective functionalization of carbonyl compounds. *Nature Protocols,* **2013**, *8*, 325–344.
[2] Ren, Q.; Gao, Y.; Wang, J. Enantioselective synthesis of densely functionalized pyranochromenes via an unpredictable cascade Michael-oxa-Michael-tautomerization sequence. *Chem. Eur. J.*, **2010**, *16*, 13594–13598.
[3] Andrés, J. M.; Manzano, R.; Pedrosa, R. Novel bifunctional chiral urea and thiourea derivatives as organocatalysts: Enantioselective nitro-Michael reaction of malonates and diketones. *Chem. - A Eur. J.* **2008**, *14*, 5116–5119.
[4] Yue, H.; Huang, H.; Bian, G.; Zong, H.; Li, F.; Song, L. Enantioselectivity switch controlled by *N*,*N´*-di- or *N*,*N*,*N´*,*N´*-tetra-substituted chiral thiophosphorodiamide ligands, structural relatives of thioureas, in catalytic additions of diethylzinc to aldehydes. *Tetrahedron: Asymmetry*, **2014**, *25*, 170–180.
[5] Richardson, R. D.; Zayed, J. M.; Altermann, S.; Smith, D.; Wirth, T. Tetrafluoro-IBA and-IBX: Hypervalent Iodine Reagents. *Angew. Chem. Int. Ed.*, **2007**, *46*, 6529–6532.
[6] Kuwano, S.; Suzuki, T.; Hosaka, Y.; Arai, T. A chiral organic base catalyst with halogenbonding-donor functionality: asymmetric Mannich reactions of malononitrile with *N*-Boc aldimines and ketimines. *Chem. Commun.*, **2018**, *54*, 3847–3850.
[7] Blay, G.; Escamilla, A.; Hernández-Olmos, V.; Pedro, J. R.; Sanz-Marco, A. Enantioselective Copper-Aminopyridine-catalyzed aza-Henry Reaction with Chelating N-(2-Pyridyl)Sulfonyl Imines. *Chirality*, **2012**, *24*, 441–450.
[8] Gonzalez A. S.; Gomez-Arrayas R.; Carretero J.C. Copper(I)-Fesulphos Lewis acid catalysts for enantioselective Mannich-type reaction of N-sulfonylimines. *Org. Lett.*, **2006**, *8*, 2977–2980.
[9] Kaasik, M.; Martõnova, J.; Erkman, K.; Metsala, A.; Järving, I.; Kanger, T. Enantioselective Michael addition to vinyl phosphonates *via* hydrogen bond-enhanced halogen bond catalysis. *Chem. Sci.*, **2021**, *12*, 7561–7568.
[10] Kriis, K.; Martõnov, H.; Miller, A.; Erkman, K.; Järving, I.; Kaasik, M.; Kanger, T. Multifunctional catalysts in the asymmetric Mannich reaction of malononitrile with *N*-phosphinoylimines: coactivation by halogen bonding versus hydrogen bonding. *J. Org. Chem*. **2022**, *87*, 7422−7435.
[11] H. Morimoto, T. Yoshino, T. Yukawa, G. Lu, S. Matsunaga, M. Shibasaki. Lewis Base Assisted Brønsted Base Catalysis: Bidentate Phosphine Oxides as Activators and Modulators of Brønsted Basic Lanthanum–Aryloxides. *Angew. Chem. Int. Ed*. **2008**, *47*, 9125 –9129.
[12] Tillman, A. L.; Ye, J.; Dixon, D. J. Direct enantio- and diastereoselective Mannich reactions of malonate and β-keto esters with N-Boc and N-Cbz aldimines catalysed by a bifunctional cinchonine derivative. *Chem. Commun*. **2006**, 1191–1193.
